# Fossil Fuel CO_2_ Emission Signatures Over India Captured by OCO‐2 Satellite Measurements

**DOI:** 10.1029/2023EF004411

**Published:** 2024-11-22

**Authors:** Vigneshkumar Balamurugan, Jia Chen

**Affiliations:** ^1^ Environmental Sensing and Modeling Technical University of Munich (TUM) Munich Germany

**Keywords:** CO2 emission, point source, Gaussian plume model, cross‐sectional flux, inventory, missing sources

## Abstract

Monitoring greenhouse gas (GHG) emissions is crucial for developing effective mitigation strategies. Recent advances in satellite remote‐sensing measurements allow us to track greenhouse gas emissions globally. This study assessed ${\text{CO}}_{2}$ emissions from various point or local sources, particularly power plants in India, using 8 years of concurrent high‐spatial resolution OCO‐2 satellite measurements. A Gaussian plume (GP) model was used to evaluate the power plant emissions reported in the Carbon Brief (CB) database. In total (39 cases), 42 different power plant ${\text{CO}}_{2}$ emissions were assessed, with 26 of them being assessed more than once. The estimated power plant ${\text{CO}}_{2}$ emissions were within ± 25% of the emissions reported in the CB database in 11 out of 39 cases and within ± 50% in 18 cases. To evaluate the EDGAR and ODIAC ${\text{CO}}_{2}$ emission inventories in terms of missing and highly underestimated sources, we estimated the cross‐sectional (CS) ${\text{CO}}_{2}$ emission flux for 45 cases. We identified the possible omission of power plant emissions in three cases for both inventories. Furthermore, we also showed 17 cases in which ${\text{CO}}_{2}$ emissions from unknown (non‐power plant) sources were highly underestimated in the EDGAR and ODIAC ${\text{CO}}_{2}$ emission inventories. Due to the simplicity of the employed approaches and their lower computational requirements compared to other methods, they can be applied to large data sets over extended time periods. This enables the acquisition of initial emission estimates for various sources, including those that are unknown and underestimated.

## Key Points

- CO_2_ emissions from power plants in India were assessed using 8 years of OCO‐2 satellite measurements
- Identified highly underestimated and missing emission sources within global emission inventories, suggesting the need for re‐evaluation
- Satellite‐based emission assessments show great promise and will improve with upcoming high‐resolution satellite missions

## 1 Introduction

Carbon dioxide (${\text{CO}}_{2}$) is a greenhouse gas (GHG) that is known to be a crucial contributor to global warming due to its high heat‐trapping ability (Pachauri et al., 2014). The current global averaged ${\text{CO}}_{2}$ concentration in the atmosphere has increased by 47% since pre‐industrial levels (WMO, 2019, 2020). Human activities such as rapid urbanization and industrialization are obvious causes of rising ${\text{CO}}_{2}$ concentrations in the atmosphere. Climate change is strongly linked to global warming, and it has an impact on ecosystem health as well as global economics. Monitoring and evaluating greenhouse gas emissions from already known and unknown emission sources is hampered due to a lack of ground‐based measurements (Boden et al., 2009; Chen et al., 2016, 2020; Dietrich et al., 2021; Fiehn et al., 2020; Forstmaier et al., 2022; Jongaramrungruang et al., 2019; Kuhlmann et al., 2021; Lan et al., 2020; Ohyama et al., 2023; Zhao et al., 2019). The bottom‐up approach has been used as a conventional emission estimation method, in which emissions were calculated by applying emission factors to known point and diffuse sources (Boden et al., 2009; Le Quéré et al., 2018). However, emission estimates from bottom‐up approaches differ significantly at different spatial scales when compared to top‐down approaches that derive emissions from real‐time atmospheric measurements (Gately & Hutyra, 2017; Gurney et al., 2019; Hutchins et al., 2017; Jones et al., 2021; Klausner et al., 2020; Z. Liu et al., 2015; Marland, 2012; Miller et al., 2013; Saunois et al., 2020; Shekhar et al., 2020; Solazzo et al., 2021; R. Wang et al., 2013).

Space‐based remote sensing measurements are becoming increasingly capable of monitoring heterogeneous emission sources at a suitable scale (Beirle et al., 2011; Brunner et al., 2023; Bovensmann et al., 2010; Ehret et al., 2022; Heymann et al., 2017; Jacob et al., 2022; Rißmann et al., 2022; Kiel et al., 2021; Kuhlmann et al., 2019; F. Liu et al., 2020; MacDonald et al., 2023; Reuter et al., 2014; Rey‐Pommier et al., 2023; Sadavarte et al., 2021; S. Wang et al., 2018; Varon et al., 2019; Zhou et al., 2022). The column‐averaged dry‐air mole fraction of ${\text{CO}}_{2}$ (${\text{XCO}}_{2}$) retrievals from previous satellite measurements, such as SCIAMACHY and GOSAT, have been shown to be useful in localizing ${\text{CO}}_{2}$ enhancements from potential emission sources (Kort et al., 2012; Schneising et al., 2008; Shim et al., 2019). The ${\text{XCO}}_{2}$ retrievals from the Orbiting Carbon Observatory‐2 (OCO‐2) satellite measurements are high‐resolution (≈ 1.29 km × 2.25 km) and high‐precision (≈1 ppm) data (Wunch et al., 2017), when compared to previous satellite measurements. Despite the fact that the OCO‐2 mission was not designed to monitor anthropogenic ${\text{CO}}_{2}$ sources, studies have shown that OCO‐2 measurements can be used to localize ${\text{CO}}_{2}$ emissions at the subcontinental (Hakkarainen et al., 2016, 2019; Hwang & Um, 2016) and urban scales (Labzovskii et al., 2019; Lei et al., 2021; Reuter et al., 2019; Schwandner et al., 2017; Wu et al., 2020; Ye et al., 2017; B. Zheng et al., 2020). OCO‐2 measurements could also be used to estimate ${\text{CO}}_{2}$ emission rates from point sources such as power plants (Hakkarainen et al., 2023; Hu & Shi, 2021; Lin et al., 2023; Nassar et al., 2017, 2021, 2022, Zheng et al., 2019). In addition, OCO‐2 measurements can be used to detect wildfire emissions (Guo et al., 2019; Reuter et al., 2019), and volcano emissions (Johnson et al., 2020). The main limitation of OCO‐2 measurements is the small swath width of about 10 km (Bhattacharjee & Chen, 2020); thus, most of the time, the OCO‐2 satellite does not overpass over the desired study region, such as an urban core or power plant. However, under certain conditions, the cross‐sectional downwind plume of ${\text{CO}}_{2}$ emissions from the desired study region could be captured by OCO‐2 when wind conditions are favorable (Reuter et al., 2019).

The goal of this study was to identify ${\text{XCO}}_{2}$ anomalies while also assessing ${\text{CO}}_{2}$ emissions at the local scale over India using high resolution OCO‐2 satellite measurements. India is the world's third largest ${\text{CO}}_{2}$ emitting country, with ${\text{CO}}_{2}$ emissions reported to have increased 3.4 times in 2018 compared to 1990 due to rapid urbanization and industrialization (Crippa et al., 2019). Coal‐consumption accounts for nearly 60% of total fossil fuel consumption in India. This highlights the importance of real‐time ${\text{CO}}_{2}$ emission monitoring in coal‐processing sectors. Therefore, we primarily focused on power plant emissions in this study. We analyzed the OCO‐2 measurements for the period from September 2014 to December 2022. To the best of our knowledge, no study has used long‐term OCO‐2 satellite measurements to report ${\text{XCO}}_{2}$ anomalies and emissions caused by different anthropogenic ${\text{CO}}_{2}$ sources over India, which has been done in this study. Studies, such as Nassar et al. (2017, 2022) have already focused on estimating emissions from point sources such as power plants using Gaussian plume model. We employed similar methods for estimating emissions, in addition, cross‐section emission flux method was employed to verify the results. This study also discusses the advantages of combining both methods. Furthermore, the emission inventories were used to interpret the emission estimates.

In addition, we aimed to evaluate global ${\text{CO}}_{2}$ emission inventories at a local or point scale, with a focus on identifying missing and significantly underestimated sources. To the best of the author's knowledge, no previous studies have focused on this aspect over India.

## 2 Data Sets Used in This Study

In this study, we used bias‐corrected ${\text{XCO}}_{2}$ retrievals from OCO‐2 satellite measurements (level‐2 & version‐11r) from 6 September 2014 to 31 December 2022. When we conducted this study, the most recent version available was OCO‐2 V11r. We noted that a new version of the data (version‐11.1r) was released during the publication of this manuscript. However, this newer version is likely to impact ${\text{XCO}}_{2}$ retrievals primarily over high latitudes (>60°N). The OCO‐2 satellite overpass occurs approximately at 13.30 local time. The spatial resolution of ${\text{XCO}}_{2}$ retrievals is ≈ 1.29 km × 2.25 km, with a ground‐track repeat time of 16 days. The ${\text{XCO}}_{2}$ retrievals product from the OCO‐2 satellite measurements consists of eight parallelogram‐shaped footprints across track, with a swath width of about 10 km (Crisp et al., 2008). This product also includes total column vapor and surface pressure, which we used to convert the modeled ${\text{CO}}_{2}$ vertical column in grams per square meter (g m^−2^) to parts per million (ppm) (Equation 3). We applied quality filtering (qa = 0; recommended by Payne et al. (2022)) to the bias‐corrected ${\text{XCO}}_{2}$ retrievals before use.

Emissions Database for Global Atmospheric Research, version: v7.0 (EDGAR) ${\text{CO}}_{2}$ emission inventory (variable: ${CO}_{2}$ *excl short‐cycle org C*) was used in this study (Crippa et al., 2019). This includes emissions from fossil sources such as fossil fuel combustion, non‐metallic mineral processes such as cement production, metal production processes, urea production, agricultural liming and solvent use. The EDGAR ${\text{CO}}_{2}$ emission inventory provides ${\text{CO}}_{2}$ emissions (kg m^−2^ s^−1^) at 0.1°× 0.1° spatial resolution for each year. The Open‐source Data Inventory for Anthropogenic ${\text{CO}}_{2}$, version: 2022 (ODIAC) data set was also used in this study (Oda et al., 2018). The ODIAC emission inventory provides ${\text{CO}}_{2}$ emissions in terms of tons of carbon per km^2^ per month. ODIAC estimates fossil fuel ${\text{CO}}_{2}$ emissions using satellite night‐time data and individual power plant emission profiles, and provides data at 1 km ×1 km spatial resolution. The EDGAR and ODIAC inventories are available only until 2021. Therefore, for the year 2022, we used data from the most recent year (2021).

We used the “ERA‐5 hourly data on pressure levels” data set for wind speed and wind direction (Hersbach et al., 2023). This data set contains wind data at 47 pressure levels with a spatial resolution of 0.25°× 0.25° and a temporal resolution of one hour. Additionally, we used wind information from the Modern‐Era Retrospective Analysis for Research and Applications, version 2 (MERRA‐2) data set (Molod et al., 2015), which has a spatial resolution of 0.5°× 0.625° and a temporal resolution of 3 hr, including 42 pressure levels.

The coordinates (geo‐location) of power plants were obtained from the Global Energy Observatory (GEO) database (GEO, 2018) and the Global Energy Monitor (GEM) wiki (GEM, 2023). Power plant ${\text{CO}}_{2}$ emissions were obtained from the Carbon Brief (CB) database (CarbonBrief, 2020). As a limitation, the CB database only provides ${\text{CO}}_{2}$ emission as annual ${\text{CO}}_{2}$ emission (Mt year^−1^), not adjusted for different years or months or days.

${CO}_{2}$ emissions from biomass burning and vegetation fires were collected from the CAMS data set (CAMS, 2023). This data set was derived from two Moderate Resolution Imaging Spectroradiometer (MODIS) instruments, with a spatial resolution of 0.1°× 0.1° for each day. This data set was utilized to analyze whether emissions from biomass burning have an influence on the estimated emissions, as the CB database and emission inventories solely encompass anthropogenic emissions.

## 3 Methods

### 3.1 Identification of ${\text{XCO}}_{2}$ Anomalies

A 0.25‐degree moving window mean was calculated along each track of OCO‐2. Anomalies in ${\text{XCO}}_{2}$ were identified when the 0.25‐degree window mean exceeded the previous and next window mean by 1 ppm, followed by a visual comparison. The identified anomalies were also compared with the albedo values given in the OCO‐2 product. If the albedo has a strong correlation with ${\text{XCO}}_{2}$ enhancements, the identified ${\text{XCO}}_{2}$ anomalies were not considered in our study as they might be related to surface‐related bias in OCO‐2 retrievals.

To identify the possible sources of identified ${\text{XCO}}_{2}$ anomalies, we looked for power plants in the upwind direction, in conjunction with ERA‐5 wind information (e.g., Figure 3a). Power plant emissions were determined using both the GP model and the cross‐sectional emission flux method, if conditions discussed below were met. The majority of power plants in India are located far from densely populated areas (e.g., urban core). Therefore, power plants located in the upwind direction could be the sole source of identified ${\text{XCO}}_{2}$ anomalies. EDGAR and ODIAC ${\text{CO}}_{2}$ emission inventories were also used to determine whether emission sources other than power plants had an influence on observed ${\text{XCO}}_{2}$ anomalies. In cases where no power plants were seen in the upwind direction, only the cross‐sectional emission flux method was used to estimate emissions, and the results were compared with EDGAR and ODIAC.

### 3.2 Gaussian Plume Model

We simulated the expected ${\text{CO}}_{2}$ enhancement for the corresponding ${\text{CO}}_{2}$ emission reported in the CB database for each power plant located in the upwind direction of the identified ${\text{XCO}}_{2}$ anomaly using a GP model, described in Bovensmann et al. (2010), as follows:

(1) $$V\left(x,y\right)=\frac{F}{\sqrt{2\pi }\cdot \sigma_{y}\left(x\right)\cdot U}\cdot e^{\frac{-1}{2}{\left(\frac{y}{\sigma_{y}\left(x\right)}\right)}^{2}},$$

(2) $$\sigma_{y}\left(x\right)=a\cdot x^{0.894},$$

where *V* is the ${\text{CO}}_{2}$ vertical column (g m^−2^), *F* is the emission rate (g s^−1^), $\sigma_{y}\left(x\right)$ is the standard deviation in the *y* direction, which depends on atmospheric stability parameter *a* in Equation 2. The atmospheric stability parameter (a) was determined via the Pasquill‐Gifford stability class, which depends on surface wind, cloud cover, and time of day (Hanna et al., 1982; Martin, 1976). It was calculated based on linear interpolation instead of stepwise classification as followed in Nassar et al. (2021). Because OCO‐2 measurements were filtered for clear‐sky days, we considered the clear‐sky category (strong insolation) to calculate the atmospheric stability parameter. Surface wind information was obtained from ERA‐5. *x* and *y* refer to the along‐wind distance and across‐wind distance. In Equation 2, *x* is specified in kilometer (km) to calculate the standard deviation in the across‐wind direction $\sigma_{y}\left(x\right)$. *U* represents the wind speed (m s^−1^) at the height of plume mid line (smokestack height + plume rise). We linearly interpolated wind information from ERA‐5 that corresponds to the OCO‐2 overpass time and plume mid line. Because information about the power plant smokestack height was unavailable, we assumed it to be 250 m (Nassar et al., 2017). The plume rise was taken as 250 m, following Brunner et al. (2019). According to Nassar et al. (2017), we manually adjusted the wind direction to match the influence of upwind sources with identified anomaly. This was done by iteratively comparing modeled enhancements for different wind directions with observed enhancements. Wind direction was chosen based on the higher correlation coefficient (R) between observed and modeled enhancements, followed by a visual comparison. Wind direction rotation was allowed within ± 60 degrees of the ERA‐5 values. This is due to the fact that we employed a reanalysis data set for wind information, which may be biased. Modeled ${\text{CO}}_{2}$ vertical column enhancement (*V*) in g m^−2^ was converted to ppm using the below Equation 3.

(3) $$XCO_{2}=V\cdot \frac{M_{\mathrm{air}}}{M_{CO_{2}}}\cdot \frac{g}{P_{\mathrm{surf}}-W\cdot g}\cdot 1000,$$

where *M* is the molecular weight (kg mol^−1^), *g* is the gravitational acceleration (m s^−2^), $P_{\mathrm{surf}}$ is the surface pressure (Pa) and *W* is the total column vapor (kg m^−2^). $P_{\mathrm{surf}}$ and *W* values were obtained from the OCO‐2 product.

${CO}_{2}$ emission rate was estimated by weighted linear least square fitting between the modeled ${\text{XCO}}_{2}$ enhancements (sum of all upwind power plants) and the observed ${\text{XCO}}_{2}$ enhancements from OCO‐2. The reciprocal of uncertainty of ${\text{XCO}}_{2}$ retrievals was used as weight. When performing the fit, we only considered the emission plume. The geographical locations of the emission plume were defined by a cutoff of at least 1% of modeled enhancements, as described in Nassar et al. (2017). The emission rate was estimated by scaling the emission reported in the CB database by a scaling factor determined from a least squares fit. This approach was used in studies, such as Hu and Shi (2021), Nassar et al. (2021, 2017), to compute the emissions from a single power plant. However, we found that other upwind power plants had a significant influence on observed enhancements in several cases. Therefore, when there were multiple power plants in an upwind direction that influence the observations, we considered them as a power plant cluster and scale their emissions together (Chen et al., 2020).

### 3.3 Cross‐Sectional Emission Flux

**Figure B1:**
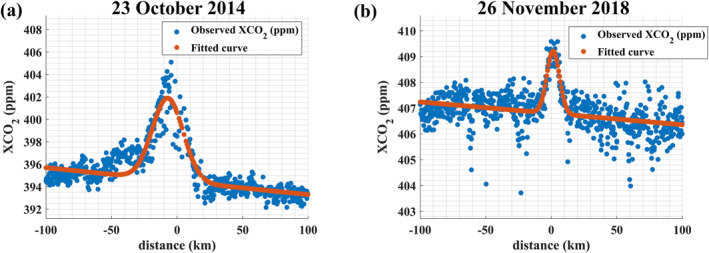
(a) The case, referred to Figure 3, where Gaussian plume (GP) with single peak is not satisfied. (b) The case, referred to Figure 5e, where GP with single peak is satisfied. Fitted curve points are based on the Equation 4.

**Figure B2:**
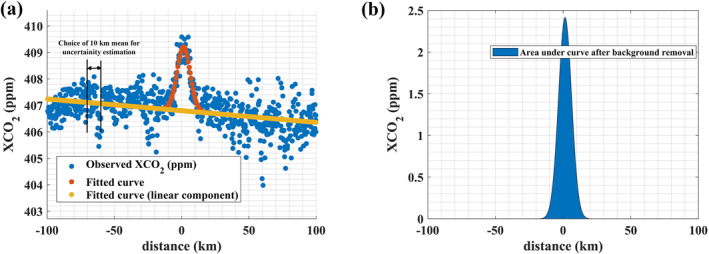
(a) The case referred to in Figure 5e is where the Gaussian plume with a single peak is satisfied (blue), and the fitted curve points are based on Equation 4 (red), along with the linear component of the fitted curve based on Equation 4 (yellow). (b) The fitted curve area after removing the background by assuming that the background is linear along the OCO‐2 track.

To verify the results of emissions estimated using GP model, we estimated the ${\text{CO}}_{2}$ emission using another method called cross‐sectional (CS) emission flux. In addition, CS emission flux method was used to assess the EDGAR and ODIAC ${\text{CO}}_{2}$ emission inventories in terms of missing and highly underestimated sources. As emissions are represented as area sources in the EDGAR and ODIAC emission inventories, GP model cannot be applied. However, the following CS emission flux method can only be applied to the identified ${\text{XCO}}_{2}$ anomalies with an isolated and single downwind plume peak (e.g., Figure B1). The following equation was fitted to the ${\text{XCO}}_{2}$ anomalies to estimate the CS ${\text{CO}}_{2}$ emission flux.

(4) $$y=m\cdot x+b+\frac{F}{\sigma \cdot \sqrt{2\pi }}\cdot e^{\frac{-{\left(x-\mu \right)}^{2}}{2{\left(\sigma \right)}^{2}}},$$

where *y* is ${\text{XCO}}_{2}$ (ppm), *F*, μ and σ are the unknown parameters (scaling constant, shift and standard deviation, respectively) that define a GP with a single peak, determined from nonlinear curve‐fitting. The part of equation *m* ⋅ *x + b* describes the linear change in background, where *x* is the distance along the OCO‐2 track. The cross‐sectional ${\text{CO}}_{2}$ emission flux (g s^−1^) was estimated by multiplying the area (g m^−1^) under the fitted curve after subtracting the background with wind speed normal to the OCO‐2 track (e.g., Figure B2); please refer to Reuter et al. (2019) and B. Zheng et al. (2020) for the detailed description of the method. In power plant cases, the wind speed value that corresponds to the plume midline was taken. For non‐power plant cases, the average wind speed below 500 m (effective wind speed) was used (B. Zheng et al., 2020). We also rejected the case if R value between observed enhancements and fitted curve is less than 0.5. Given that our objective was to identify missing or highly underestimated sources, the estimated cross‐sectional ${\text{CO}}_{2}$ emission flux was then compared with the inventory ${\text{CO}}_{2}$ emissions within a 50‐km upwind range. The 50‐km upwind range was chosen based on the previous study (B. Zheng et al., 2020), which demonstrated that relating the CS emission flux with emission sources in the 50‐km upwind range is reasonable. Due to the unavailability of temporal (diurnal/weekly/seasonal) changes of ${\text{CO}}_{2}$ emissions, we directly extrapolated this instantaneous CS emission flux (g s^−1^) to annual mean emissions (Mt year^−1^).

### 3.4 Background Selection

To calculate the observed ${\text{XCO}}_{2}$ enhancements, the background was removed from ${\text{XCO}}_{2}$ retrievals by assuming that the background is linear along the OCO‐2 track (Reuter et al., 2019; B. Zheng et al., 2020). This was done by fitting Equation 4 to the observed ${\text{XCO}}_{2}$ measurements, and then subtracting the linear component *m* ⋅ *x + b* from the observed ${\text{XCO}}_{2}$ measurements (e.g., Figure B2).

### 3.5 Uncertainty Estimation

The uncertainty in the background concentration is the major uncertainty in calculating the observed enhancement, whereas the uncertainty in the wind speed leads to the major uncertainty in modeling the enhancement. In addition, emission uncertainties related to plume rise were also considered. The emission uncertainty was calculated as follows (Nassar et al., 2022),

(5) $$\epsilon =\sqrt{\epsilon_{w}^{2}+\epsilon_{b}^{2}+\epsilon_{pr}^{2}},$$

where $\epsilon_{w}$ represents the uncertainty due to the wind speed, calculated as the difference in emission estimate for wind speed from ERA‐5 values and MERRA‐2. The term $\epsilon_{b}$ represents the uncertainty due to the background, calculated as the standard deviation in emission estimates for the four different background choices instead of a linear fit. The four different choices of background were chosen at random by taking the 10 km mean outside of the emission plume (e.g., Figure B2). Emission uncertainties related to plume rise are represented as $\epsilon_{pr}$. This value was calculated as the standard deviation in emission estimates for the plume rise values of an ensemble of emission estimates, assuming plume rise values of 100, 200, 250, 300, and 400 m (Nassar et al., 2021). For non‐power plant cases, $\epsilon_{pr}$ was not considered.

## 4 Results

### 4.1 Mean Spatio‐Temporal Variation of ${\text{XCO}}_{2}$ and ${\text{XCO}}_{2}$ Anomalies Over India

**Figure 1:**
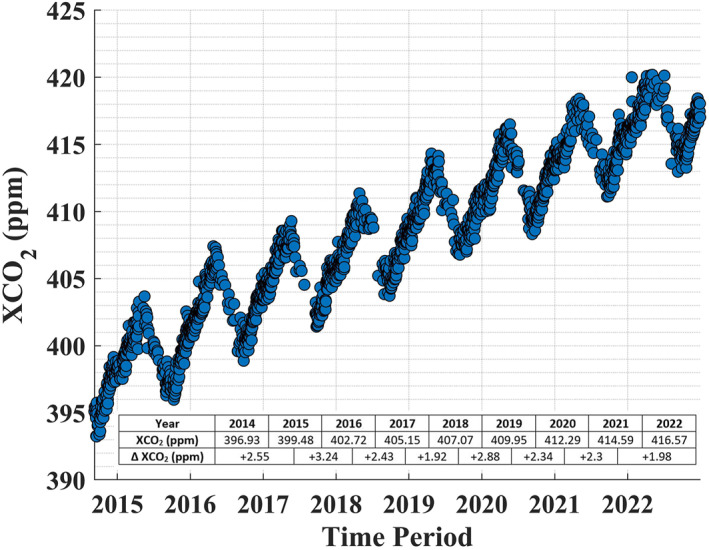
Inter and intra‐annual ${\text{XCO}}_{2}$ variability over India (8–36.5°N, 67.5–98°E). The daily mean of ${\text{XCO}}_{2}$ retrievals from OCO‐2 measurements is shown for the period from 6 September 2014 to 31 December 2022. The table inset show the yearly mean ${\text{XCO}}_{2}$ (row 2), and a year‐to‐year increase of ${\text{XCO}}_{2}$ compared to the previous year (row 3).

**Figure 2:**
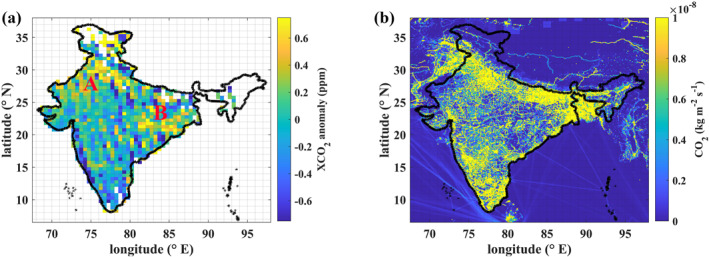
(a) Mean spatial variability of ${\text{XCO}}_{2}$ anomalies for the period from 6 September 2014 to 31 December 2022 at 0.5‐degree grid (only with at least 5 measurements over each grid are shown). A refers to the Indian states of Punjab and Haryana. B refers to West Bengal, Jharkhand, Odisha, and Chhattisgarh. (b) EDGAR ${\text{CO}}_{2}$ emission inventory for 2021‐year.

First, we examined the mean spatio‐temporal variation of ${\text{XCO}}_{2}$ and ${\text{XCO}}_{2}$ anomalies over India (Figures 1 and 2). The temporal variation in mean ${\text{XCO}}_{2}$ provides insight into the variation in background ${\text{CO}}_{2}$ concentration, whereas the spatial variability of ${\text{XCO}}_{2}$ anomalies provides insight into the presence of potential large scale sources (Hakkarainen et al., 2016, 2019). We considered all days between 6 September 2014 and 31 December 2022 with at least 500 OCO‐2 measurements on a single day to create the mean spatio‐temporal variability of ${\text{XCO}}_{2}$ and ${\text{XCO}}_{2}$ anomalies over India. The daily mean of ${\text{XCO}}_{2}$ retrievals from OCO‐2 measurements over India is shown in Figure 1. The intra‐annual variability of ${\text{XCO}}_{2}$ retrievals from OCO‐2 measurements follows an expected seasonal cycle, with a steady increase from October to April and a steady decrease from May to September (Singh et al., 2022). This is primarily driven by the plants through the processes of photosynthesis (sink of ${\text{CO}}_{2}$) in the spring and summer, and respiration (source of ${\text{CO}}_{2}$) in the fall and winter. Furthermore, higher fossil‐fuel consumption in the winter due to heating purposes contributes to higher ${\text{CO}}_{2}$ concentrations in the winter. It is important to note that the number of available OCO‐2 measurements was lower in the summer due to the presence of clouds in the monsoon season (Sen Roy et al., 2015). As reported (WMO, 2019, 2020), ${\text{CO}}_{2}$ levels have been rising; the average year‐to‐year increase rate of ${\text{XCO}}_{2}$ from OCO‐2 over India was about 2.45 ppm over the study period. WMO (2020) reported a global increase of 2.37 ppm per year over the last decade.

To obtain the mean spatial variability of ${\text{XCO}}_{2}$ enhancements (${\text{XCO}}_{2}$ anomalies) caused by the potential emission sources over India (Figure 2a), we subtracted the background concentration from the daily ${\text{XCO}}_{2}$ retrievals. For each specific overpass, we chose the median value of ${\text{XCO}}_{2}$ over India as the background concentration (Hakkarainen et al., 2016). The estimated ${\text{XCO}}_{2}$ anomalies were then spatially averaged over the study period. Positive ${\text{XCO}}_{2}$ anomalies were most noticeable over Northern India. The main two hotspots (A and B in Figure 2a) were identified over India; A refers to the Indian states of Punjab and Haryana, while B refers to West Bengal, Jharkhand, Odisha, and Chhattisgarh. Both of these hotspot regions have a high density of coal‐fired power plants (refer to https://vedas.sac.gov.in/energymap/view/powergis.jsp). Hotspot (A) also coincides with the location of extensive crop residue burning, which is further corroborated by MODIS satellite measurements (T. Liu et al., 2021). Although hotspots A and B coincide with higher emissions as given in EDGAR (Figure 2b), it can be seen that ${\text{XCO}}_{2}$ enhancements in southern India did not show higher emissions as depicted in EDGAR. One possible reason is that southern India has very few coal power plants; therefore, OCO‐2 is not able to detect enhancements from diffuse area sources effectively.

### 4.2 Assessment of the ${\text{CO}}_{2}$ Emission Rate

According to the method described in Section 3.1 (identification of ${\text{XCO}}_{2}$ anomalies), we examined 955 days of available OCO‐2 measurements over India, from 6 September 2014, to 31 December 2022, to identify ${\text{XCO}}_{2}$ anomalies at each overpass. In total, 39 ${\text{XCO}}_{2}$ anomalies (cases) were considered to estimate emissions from power plants using the GP model. These 39 cases were chosen based on four conditions: 1. Power plants were located within 50 km of the observed plume. 2. Wind direction was not parallel to the OCO‐2 track. This is because the assumption of a linear background along the OCO‐2 track is no longer valid when the wind flows along the OCO‐2 track. 3. Wind speed at plume mid line was more than 1 m/s because in lower wind speed conditions, wind information are expected to be more uncertain. 4. The correlation coefficient between modeled and observed enhancements was more than 0.5.

From these 39 cases, we identified emission signals from 42 different power plants, 26 of which were assessed multiple times (refer to Table C1). These 42 power plants were grouped into 12 clusters, and three single power plant cases (refer to Table C2). In the main text, we focused on three clusters (Cluster 1, 2, and 11). Cluster 1 and 2 were selected as prime examples to showcase OCO‐2's capability in detecting emission changes resulting from the addition of new units or new power plants. Cluster 11 was also discussed in the main text, demonstrating that estimated emissions using the GP model were affected by sources other than power plants. Remaining clusters and single power plant cases were discussed in the Appendix A.

#### 4.2.1 Cluster 1

**Figure 3:**
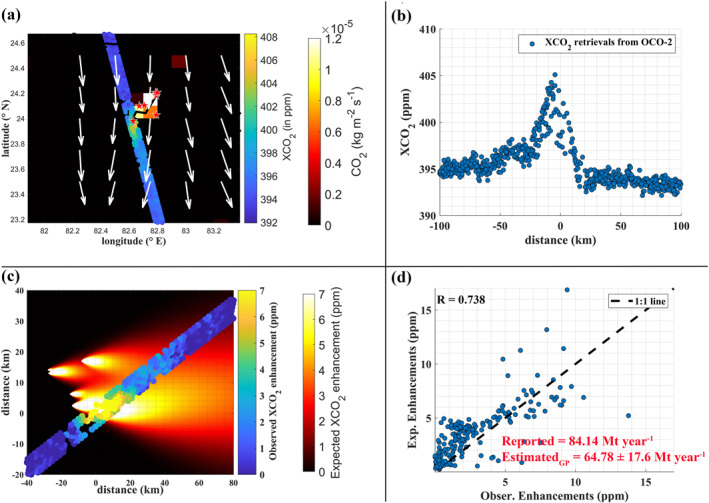
(a) ${\text{XCO}}_{2}$ retrievals from OCO‐2 measurements on 23 October 2014 were overlaid on the EDGAR ${\text{CO}}_{2}$ emission inventory. White arrow represents wind data from ERA‐5 at the OCO‐2 overpass. Power plant locations were represented by red stars. (b) ${\text{XCO}}_{2}$ retrievals were plotted against the distance between the peak of observed ${\text{XCO}}_{2}$ and OCO‐2 measurements. (c) Observed ${\text{XCO}}_{2}$ enhancements were overlaid on the modeled ${\text{XCO}}_{2}$ enhancements (sum of all upwind power plant signals). (d) Comparison between modeled and observed ${\text{XCO}}_{2}$ enhancements.

On 23 October 2014, the OCO‐2 satellite passed over the Sasan Ultra Mega Coal Power Plant in Madhya Pradesh state (Figure 3a). The observed ${\text{XCO}}_{2}$ enhancements reached up to 14 ppm, with most of the enhancements being within 5 ppm (Figure 3d). However, in the upwind direction, we noticed a group of power plants, which may had an impact on the observed ${\text{XCO}}_{2}$ enhancements. The influence of upwind power plant emissions on the observed ${\text{XCO}}_{2}$ enhancements depends on the emission strength and location. We used the GP model to simulate the expected ${\text{XCO}}_{2}$ enhancements using previously reported ${\text{CO}}_{2}$ emissions in the CB database for each power plant. The reported ${\text{CO}}_{2}$ emissions in the CB database for the Sasan Ultra Mega Power Plant, Vindhyachal STPS Coal Power Plant, Singrauli Super Coal Power Plant, Rihand Coal Power Plant, Renusagar Power Station, Anpara Coal Power Plant and Anpara‐C Power Station were 16.45, 23.76, 11.42, 14.17, 4.32, 8.94, and 5.08 Mt year^−1^, respectively. Sasan Ultra Mega Power Plant, Vindhyachal STPS Coal Power Plant and Singrauli Super Coal Power Plant were the main contributors for the observed plume. Despite being 18 and 30 km away from the peak ${\text{XCO}}_{2}$ enhancement, the Rihand, Renusagar and Anpara power plants collectively exert an influence of up to 2–2.5 ppm on OCO‐2 measurement locations. Therefore, the observed plume from OCO‐2 was influenced by all seven power plants. The model simulations captured these enhancements well as shown in Figure 3d. The correlation coefficient (R) between modeled and observed plume ${\text{XCO}}_{2}$ enhancements was 0.738. The estimated ${\text{CO}}_{2}$ emission rate (64.78 ± 17.6 Mt year^−1^) using the GP model was lower than that in the CB database (84.14 Mt year^−1^). Both the Sasan Ultra Mega Power Plant (Unit‐6) and the Vindhyachal STPS Coal Power Plant (Unit‐13) commissioned new units in 2015 (refer to https://www.gem.wiki/Sasan_Ultra_Mega_Power_Project and https://www.gem.wiki/Vindhyachal_power_station). The emission rate provided for these power plants in the CB database includes these new units, which were not present during the 2014 measurements. Therefore, the estimated emission rate on 23 October 2014, is lower than that in the CB database.

**Figure 4:**
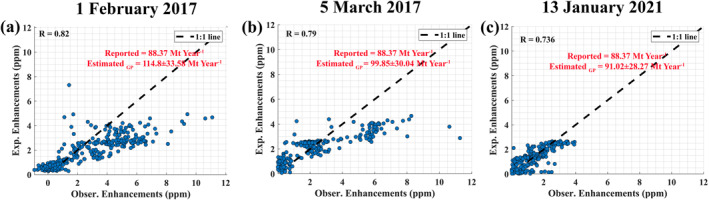
Modeled and observed ${\text{XCO}}_{2}$ enhancements for the case referred in Figure 3, but for different days. In addition to the power plants considered in Figure 3, Anpara‐D Power Station emission was included in the model for these three cases as it was commissioned after 2015.

We also found three more ${\text{XCO}}_{2}$ anomalies that were influenced by all seven power plants at different time periods (1 February 2017, 5 March 2017 and 13 January 2021). Apart from the new units in Sasan Ultra Mega Power Plant (Unit‐6) and Vindhyachal STPS Coal Power Plant (Unit‐13), a new power plant, Anpara‐D Power Station, was commissioned in 2015. Which was commissioned in 2015, with 4.32 Mt year^−1^ emission rate reported in the CB database. For these three cases, which occurred after 2015, we included the Anpara‐D Power Station into our model. The estimated ${\text{CO}}_{2}$ emission rates using GP model were 114.8 ± 33.58 Mt year^−1^, 99.85 ± 30.04 Mt year^−1^ and 91.02 ± 28.27 Mt year^−1^, for 1 February 2017, 5 March 2017 and 13 January 2021 cases (Figure 4). The emission estimates of these days were higher compared to those on 23 October 2014. This can be attributed to the emissions from new units and a new power plant. The estimated emission rates from the 7 power plants were also slightly higher than in the CB database (88.37 Mt year^−1^) for these three cases. The uncertainties associated with these estimates were 29.25%, 30.01%, and 31% respectively. For the above discussed cases, uncertainties from different components, according to Equation 5 given in Table B1. It can be seen that the uncertainty from the choice of background dominates the total uncertainty, followed by the uncertainty due to wind speed.

Only the 13 January 2021 case allowed us to estimate the CS ${\text{CO}}_{2}$ emission flux (75.32 ± 27.4 Mt year^−1^). The other three cases, did not satisfy the conditions for applying the CS method (e.g., Figure B1a). It is important to note that these results were also influenced by the fact that some power plants emitted higher/lower emission than reported in the CB database. Power plant emissions vary depending on energy demand, as well as the type of coal used. These information are not publicly available, and are not adjusted in emissions provided by CB database.

#### 4.2.2 Cluster 2

**Figure 5:**
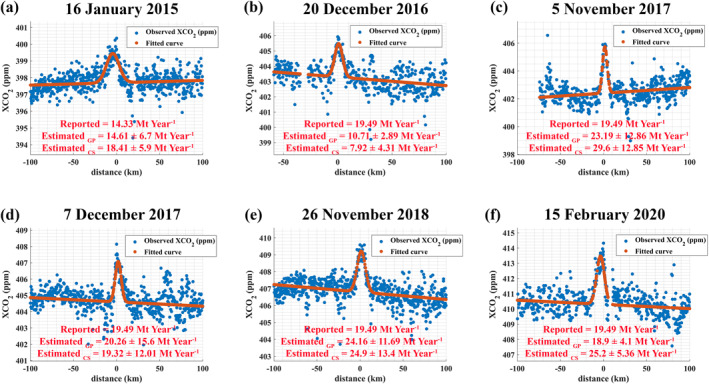
Observed ${\text{XCO}}_{2}$ measurements from OCO‐2 (blue points) and fitted curve points (red points) based on the Equation 4 for the case of Ramagundam STPS Coal Power Station, Ramagundam B (RTS‐B) Coal Power Station and Pegadapalli (Jaipur Mandal) Power Station. The Pegadapalli (Jaipur Mandal) Power Station was not commissioned in 2015, therefore its emissions were not included in the model for the 1 January 2015 case.

We found six cases in Telangana state that allowed us to estimate the ${\text{CO}}_{2}$ emissions over different time periods (Figure 5). We considered the Ramagundam Power Station and the Ramagundam B (RTS‐B) Coal Power Station for the overpass on 16 January 2015. Additionally, we included the Pegadapalli Power Station for the remaining five cases since it was commissioned in 2016. For all cases, the estimated emissions using the GP model were within ± 50% of the reported emission: the estimated scaling factor ranges between 0.55 and 1.24. The cross‐sectional ${\text{CO}}_{2}$ emission flux for these cases can also be estimated because OCO‐2 captures the downwind plume with a single and isolated peak. The estimated CS ${\text{CO}}_{2}$ emission fluxes for these six cases were 18.41 ± 5.99 Mt year^−1^, 7.92 ± 4.31 Mt year^−1^, 29.6 ± 12.85 Mt year^−1^, 19.32 ± 12.01 Mt year^−1^, 24.9 ± 13.4 Mt year^−1^ and 25.2 ± 5.36 Mt year^−1^, respectively. These CS emission estimates were comparable to the emission estimated using GP model. For the 20 December 2016 case, the estimated emission using the GP model was significantly lower than the reported emission, a result further supported by the CS emission flux method. Data from the GEM and CB databases indicate that the Pegadapalli Power Station began operating in 2016. On 20 December 2016, the lower estimated emissions could indicate that either the Pegadapalli Power Station had not yet started operating or that it was shut down in Ramagundam or Pegadapalli Power Station.

#### 4.2.3 Cluster 8

We observed three emission plumes corresponding to a cluster of four plants in West Bengal state (Durgapur Steel City Power Station, Durgapur SAIL Power Station, Durgapur Projects Limited Power Station and Mejia Power Station) on 19 November 2014, 16 March 2017, and 29 December 2017. The estimated emissions for these three cases were significantly higher (56.77 ± 12.98, 71.33 ± 36.71, and 55.38 ± 19.3 Mt year^−1^, respectively) than the reported emissions in the CB database (20.98 Mt year^−1^). In fact, the estimated emissions were about 2–3 times higher than the reported values. Based on the information from GEM, no new units or power plants were commissioned/planned. In the EDGAR emission inventory, the emissions provided within a radius of approximately 50 km in the upwind direction of the observed plume were 53.84, 76.01, and 61.86 Mt year^−1^, on 19 November 2014, 16 March 2017, and 29 December 2017, respectively. Since the estimated emissions were high, the higher EDGAR emissions might also suggest the existence of other emission sources besides the considered power plants. However, the emissions provided in the ODIAC were 19.67, 25.08, and 23.07 Mt year^−1^, on 19 November 2014, 16 March 2017, and 29 December 2017, respectively, which is approximately equal to the emissions provided in the CB database. This suggests that possibly the ODIAC did not take into account the other emission sources around the power plants. Furthermore, we examined emissions from biomass within a radius of approximately 50 km in the upwind direction of the observed plume. However, the emissions from biomass were found to be very low. The emissions from biomass on these 3 days were 0, 0, and 0.12 Mt year^−1^, respectively.

#### 4.2.4 Summary

**Figure 6:**
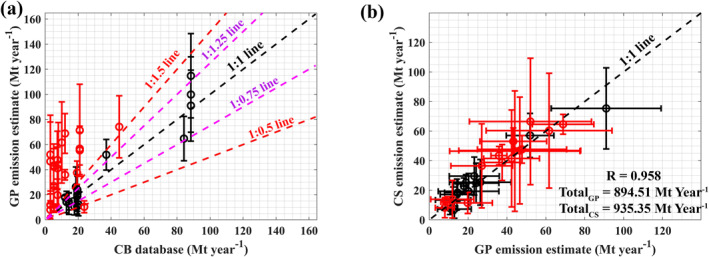
(a) Comparison between ${\text{CO}}_{2}$ emissions estimated using the Gaussian plume (GP) model and emission reported in the Carbon Brief (CB) database for power plants (39 cases). (b) Comparison between ${\text{CO}}_{2}$ emissions estimated using Gaussian plume (GP) model and cross‐sectional (CS) emission flux method (28 cases). The cases in which the estimated ${\text{CO}}_{2}$ emissions from the GP model higher or lower than (±) 50% of the reported emission in the CB database are marked with red markers in (a) and (b).

In Table S1 in Supporting Information S1, the dates and locations of identified anomalies, reported emissions in the CB database, estimated emissions using the GP model and CS flux method, and emissions reported in EDGAR, ODIAC and CAMS biomass data are provided for power plant cases. Figures similar to Figure 3 for all analyzed cases are provided in the Supporting Information S1. Through analyzing all 39 cases, it was observed that OCO‐2 measurements were able to detect small to large ${\text{CO}}_{2}$ enhancements (≈1–14 ppm) caused by various sources. The estimated ${\text{CO}}_{2}$ emissions from these sources range from 8.05 to 114.8 Mt year^−1^ in our study. This highlights its capability in detecting a wide range of sources. Out of 39 cases, 11 cases showed estimated emissions were within ±25% of the emissions reported in the CB database, while 18 cases were within ±50% (Figure 6). On the other hand, 17 cases exhibited very high emissions (above 2 times the reported emissions), potentially influenced by other emission sources alongside power plants. The CS emission flux was also estimated for 28 cases out of the 39, and it demonstrated strong agreement with the emissions estimated from the GP model. In particular, the CS emission flux method confirmed cases with higher emission rates from the GP model compared to the CB database (Figure 6b). Though without uncertainty of wind information, in many cases, applied methods along with OCO‐2 measurements were able to detect the changes in emissions due to the addition of new units or new power plants (e.g., cluster 1, 2, and 3).

**Figure 7:**
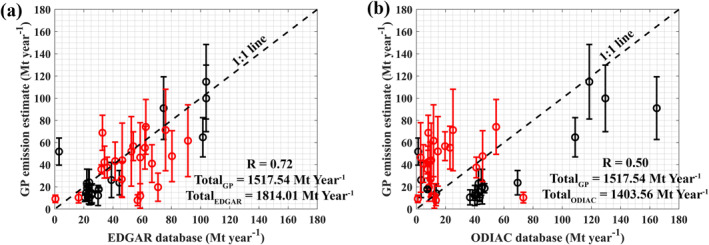
Comparison between ${\text{CO}}_{2}$ emissions estimated using the Gaussian plume (GP) model and the emission reported in EDGAR (a) and ODIAC (b) emission inventories within 50‐km upwind range (Power plant cases: 39 cases). The cases in which the estimated ${\text{CO}}_{2}$ emissions using the Gaussian plume (GP) model higher or lower than (±) 50% of the reported emission in the Carbon Brief database are marked with red markers.

For power plant cases, the EDGAR emission inventory agreed well when compared to GP estimates, especially in cases influenced by additional emission sources other than power plants (Figure 7). On the other hand, ODIAC showed poor agreement, notably indicating lower emissions in cases with high estimated emissions from the GP model. This implies that ODIAC might not have included the emission sources surrounding the power plants. It should be noted that both EDGAR and ODIAC did not include or highly underestimated the emissions from Tata Mundra Ultra Mega Power Project, Mundra Thermal Power Project, and Kawai Thermal Power Project. Additionally, Dongamahua Captive Power Plant and Tamnar Power Station might not be included or highly underestimated in the ODIAC emission inventories.

## 5 Missing and Highly Underestimated Sources in EDGAR and ODIAC Emission Inventories

**Figure 8:**
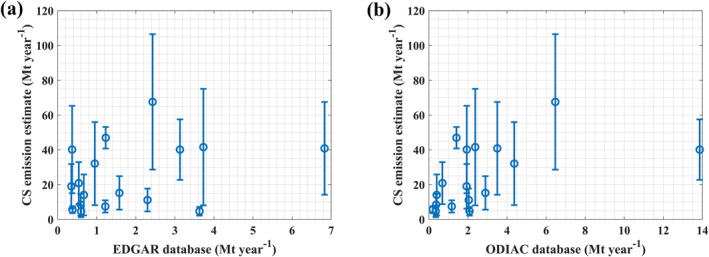
Comparison between ${\text{CO}}_{2}$ emissions estimated using cross‐sectional (CS) flux method and the emission reported in EDGAR and ODIAC emission inventories within 50‐km upwind range (non power plant cases: 17 cases).

In total, the CS emission flux was estimated for 45 cases with single and isolated peaks, 28 of which corresponded to the power plant emission cases discussed above. The missing and highly underestimated power plant emissions in EDGAR and ODIAC inventories were discussed in the previous section. In this section, we compared the CS emission flux of non‐power plant cases (17 cases) with EDGAR and ODIAC (Figure 8).

**Figure 9:**
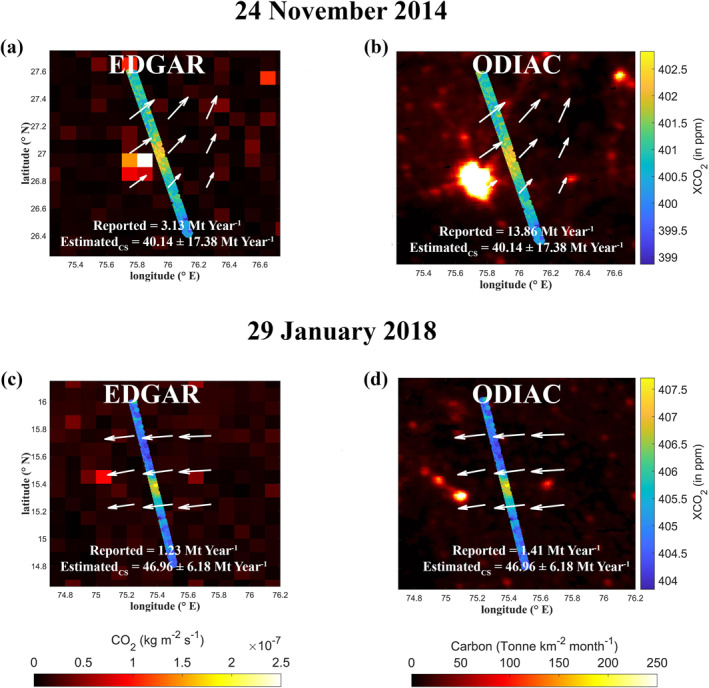
The example two cases where CS emission estimates from OCO‐2 were significantly higher than emissions provided in the EDGAR and ODIAC inventories.

The estimated CS emission flux ranged between 4.68 and 67.58 Mt year^−1^. These emissions were 1.3–108.6 times higher than the emissions provided in the EDGAR inventory, whereas they were 2.26–35.25 times higher than the emissions provided in the ODIAC inventory. In these cases, it can be seen that there is a presence of a source over the upwind region of the observed plume (e.g., Figure 9). This implies a high underestimation of emissions over these regions. In further studies, the type of sources will be investigated. For non‐power plant cases, in Table S2 and Figure S1 in Supporting Information S1, the dates and locations of identified anomalies are provided, along with the estimated emissions using the CS flux method, reported emissions in EDGAR, ODIAC, and CAMS biomass data. It can also be noted that biomass events have a very small impact on these identified anomalies.

The discrepancies between estimated emissions and those provided in inventories are not surprising, considering that studies such as Gately and Hutyra (2017) and Gurney et al. (2019) have revealed that global inventories typically exhibit high uncertainty at the local scale. EDGAR and ODIAC use different approaches to estimate emissions: EDGAR uses activity data with spatial proxies such as population and road density, whereas ODIAC primarily uses space‐based nighttime light data, which may underestimate ${\text{CO}}_{2}$ emissions (Gately & Hutyra, 2017).

## 6 Conclusions

This study assessed the anthropogenic ${\text{CO}}_{2}$ emissions, particularly from power plants, using concurrent high‐resolution OCO‐2 measurements over India. We examined the data from September 2014 to December 2022, a period of more than 8 years. We considered 39 ${\text{XCO}}_{2}$ cases that were influenced by power plants. The GP model was used to estimate the power plants emission. In 11 out of 39 cases, we found that the estimated ${\text{CO}}_{2}$ emissions for power plants using GP model were within ± 25% of the ${\text{CO}}_{2}$ emission reported in the CB database, whereas 18 cases within ± 50%. In total, 42 different power plants were considered in our study, with 26 of them being considered more than once. Our study also showed that the cases with high emissions were strongly influenced by emission sources other than power plants. Emission estimations based on the CS emission flux method, including knowledge from multiple sources such as emission inventories, may be used to conform to these cases. We also demonstrated the capability of OCO‐2 in detecting cases with changes in emissions due to the addition of new units or new power plants.

To evaluate the EDGAR and ODIAC ${\text{CO}}_{2}$ emission inventories, we selected 45 cases with isolated and single peak downwind plumes to estimate the cross‐section (CS) emission flux. When comparing our CS emission estimate for power plants, EDGAR showed better agreement than ODIAC. Besides the absence of power plant emissions in both the EDGAR and ODIAC inventories (3 cases), we also identified 17 highly underestimated and missing sources in the inventory. These sources will be studied in future research.

Although the GP model is commonly used to model point source emission, it can fail over longer distances because it assumes constant wind speed and wind direction. The main source of uncertainty in modeling the expected enhancements and CS emission estimates was wind data, that is, emission estimates are directly proportional to wind speed. The study relies on hourly ERA 5 reanalysis data, which is comprehensive; however, it would benefit from additional uncertainty information to improve the precision of emission estimation. Accurate wind measurements or extensive transport modeling work could reduce errors in calculating emissions. Because our method requires less computation than other methods, it can be used as a first step toward discovering the missing or underestimated emission source and its initial emission, from which more advanced methods, such as Bayesian inversion combined with extensive transport modeling, such as STILT and XSTILT, to estimate emission with less uncertainty can be conducted. The missing and highly underestimated sources in emission inventories and databases can then be routinely updated.

Given these findings, utilizing OCO‐2 data for Measuring, Reporting, and Verification (MRV) systems for ${\text{CO}}_{2}$ emissions presents a valuable addition as it can be used for a wide range of sources. However, the OCO‐2 satellite has a ground‐track repeat time of 16 days with a small swath width, which hinders the continuous monitoring of specific emission sources. In addition, its measurements are influenced by cloud cover and other atmospheric conditions, potentially affecting data quality and availability. These factors must be considered when integrating OCO‐2 data into MRV frameworks. The enhanced possibility of continuous monitoring of local‐scale ${\text{CO}}_{2}$ emission sources is achievable with high spatial and temporal resolution satellite measurements with a wide swath width. Recent and upcoming satellite missions, such as OCO‐3 (in SAM mode), Microcarb, Geocarb, CO2Image, Tansat‐2, and CO2M, aim to achieve this.

The detection of unknown and underestimated emission sources underscores the necessity for more comprehensive emission inventories. Policymakers can use these findings to implement mitigation strategies targeting non‐compliant emission sources, thereby aiding India's adherence to international climate commitments for emission reduction. Moreover, transitioning to cleaner technologies and implementing emission reduction strategies can lead to more sustainable energy production, directly benefiting socioeconomic conditions and human health.

## Supporting information

Supporting Information S1

## Data Availability

The manuscript (OCO‐2/OCO‐3, 2022) utilized ${\text{XCO}}_{2}$ retrievals from OCO‐2 satellite measurements, accessible upon registration. Hourly ERA5 wind information from the fifth generation ECMWF reanalysis is credited to Hersbach et al. (2023) and can be accessed with registration. Global emission inventory data, such as EDGAR (Alfredo et al., 2022) and ODIAC (Oda & Maksyutov, 2022), were employed and are accessible without registration.

## Appendix A Assessment of Emission Rate

The emission assessment for the remaining nine clusters and three individual power plants is discussed here.

### A1 Cluster 3

Chandrapur Thermal Power Station, Dhariwal Power Station and Ghugus Power Station were identified as the sources of the observed plume on 16 January 2015. The estimated emission using GP model (12.31 ± 9.08 Mt year^−1^) was significantly lower than in the CB database (20.19 Mt year^−1^). The cross‐sectional emission flux method also supported this finding, with an estimated emission of 13.4 ± 8.8 Mt year^−1^ for this particular case. The commissioning of new units took place at Chandrapur Thermal Power Station. Unit‐8 commenced operations after May 2015, and unit‐9 followed in March 2016. However, the emission data provided in the CB database for Chandrapur Thermal Power Station included all units, which explains the discrepancy in the estimates. On 22 December 2016, the Chandrapur Thermal Power Station was the only source of the observed plume, with all units expected to be operational. The estimated emission using the GP model was found to be 12.97 ± 8.65 Mt year^−1^, which is 0.9 times the emission provided in the CB database (14.42 Mt year^−1^). However, the CS emission flux yields a low emission (7.3 ± 6.2 Mt year^−1^).

### A2 Cluster 4

On 25 January 2017, the estimated emission using the GP model (23.83 ± 10.94 Mt year^−1^) for the case of Angul Power Station and Talcher Kaniha Super Thermal Power Station was 1.22 times the emission reported (19.54 Mt year^−1^) in the CB database. On 15 November 2019, there was an additional source to consider, Angul Steel Power Station, but its influence was relatively low (less than 0.5 ppm). The estimated emission using GP model (10.43 ± 4.7 Mt year^−1^) on 15 November 2019 was significantly lower than in the CB database (23.72 Mt year^−1^). The information on coal consumption/power production and maintenance might explain this discrepancy. It is also important to note that biomass burning (≈1.5 Mt year^−1^) also had a small influence on these two overpasses (Table S1 in Supporting Information S1).

### A3 Cluster 5

The emission rate of the Tata Mundra Ultra Mega Power Project and the Mundra Thermal Power Project (considered as a cluster of two power plants) was estimated on 3 October 2018. It was found to be 1.4 times the reported emission in the CB database, amounting to 51.89 ± 8.54 Mt year^−1^, whereas the CB database reported 37.07 Mt year^−1^. The emission rate was calculated using the CS emission flux method, resulting in an estimate of 57.04 ± 14.92 Mt year^−1^. It is important to emphasize that the emissions provided in the EDGAR and ODIAC inventories within 50 km radius of the observed plume in the upwind direction were 2.99 and 1.27 Mt year^−1^ respectively. This suggests that either these two power plants were not included, or they were significantly underestimated in both inventories.

### A4 Cluster 6

A cluster of two power plants (Dongamahua Captive Power Plant and Tamnar Power Station) identified as a source for the identified anomaly on 13 January 2021, with a scaling factor of 1.41 from the GP model (26.29 ± 15.85 Mt year^−1^). The CS emission flux was 25.44 ± 15.43 Mt year^−1^. It is important to note that the emissions provided in the ODIAC inventory within 50 km of the observed plume in the upwind direction was 3.28 Mt year^−1^, which is too low compared to the estimated emissions and reported emission in the CB database. This indicates that these power plants were either not included or highly underestimated in the ODIAC inventory. On other hand, the emissions provided in the EDGAR inventory within 50 km of the observed plume in the upwind direction was 38.88 Mt year^−1^.

### A5 Cluster 7

On 10 January 2018, the estimated emission using the GP model (10.65 ± 6.7 Mt year^−1^) for Khaperkheda Power Station and Koradi Thermal Power Station was lower than the reported emission in the CB database (17.76 Mt year^−1^). The reason for this discrepancy is unknown and requires further investigation. To clarify the situation and explain the lower emission, it is essential to cross‐check the data with coal consumption/power production and maintenance information for both power stations.

### A6 Cluster 9

Over Jharkhand state, we examined the emissions of Jojobera Power Plant, Jamshedpur Works Power Station, and Adityapur Works Power Station (as a cluster of three power plants) on 18 January 2017, 28 November 2020, and 31 October 2022. In addition to the aforementioned power plants, the Mahadev Prasad Super Thermal Power Plant also had an influence on the observed plume on 7 January 2018. The estimated emissions from the GP model were 8.32, 8.15, 5.2, and 5.52 times higher than in the CB database, respectively. The CS emission flux method also confirms this findings (Table S1 in Supporting Information S1). The EDGAR emission inventory indicated high emissions around the observed plume, potentially suggesting the existence of other emission sources. In contrast, ODIAC indicates no potential emission sources besides power plants.

### A7 Cluster 10

We have also identified another power plant cluster over the Jharkhand state, consisting of Bokaro Steel City Thermal Power Station and Chandrapura Power Station, with emission signatures on three different dates: 30 December 2014, 31 January 2015, and 18 January 2022. The estimated scaling factors from the GP model for these cases were 2.65, 5.5, and 6.39, respectively. Additionally, on 31 January 2021, the Santaldih Thermal Power Station contributed to the observed plume, with an estimated scaling factor of 6.1. Notably, the emission estimated using the GP model on 30 December 2014 was lower compared to the other overpasses, a finding that was supported by the CS emission flux method (Table S1 in Supporting Information S1). The higher emissions observed over this power plant cluster can be explained by EDGAR's report, which indicated the presence of other emission sources (Table S1 in Supporting Information S1). Similar to the latter discussed cases, ODIAC reported emissions approximately equal to the CB database, possibly indicating the exclusion of secondary emission sources apart from power plants.

### A8 Cluster 11

Similar to cluster 9 and 10, a case for the cluster of two power plants (Bellary Thermal Power Station and JSW Vijayanagar Toranagallu Power Station), different scaling factors (1.48, 5.7, and 2.97; 17.87 ± 1.38, 68.85 ± 15.82 and 35.87 ± 4.1 Mt year^−1^) were estimated on 4 March 2018, 18 January 2019, and 24 February 2021. The CS emission fluxes also show similar variation during these days. The emission in upwind direction of these observed anomalies was ≈30 Mt year^−1^ in the EDGAR and 8 Mt year^−1^ in the ODIAC inventories. This scenario is similar to clusters 9 and 10 in that EDGAR suggests the presence of secondary emission sources, whereas ODIAC does not.

### A9 Cluster 12

On 24 February 2015, the emission rate for a cluster of three power plants (Neyveli Thermal Power Station I, Neyveli Thermal Power Station II and Neyveli Zero power station) from the GP model were estimated (37.55 ± 9.29 Mt year^−1^), which is 1.94 times the reported emission in the CB database (19.96 Mt year^−1^). The CS emission flux yielded similar findings (38.8 ± 12.15 Mt year^−1^). Both EDGAR and ODIAC data also provides high emission (36 and 40 Mt year^−1^) in the upwind direction of observed plume within 50 km, suggesting the presence of possible other emission sources.

### A10 Sipat Power Station

The estimated emission for the Sipat Power Station on 1 March 2018, using the GP model (14.24 ± 8.54 Mt year^−1^) and the CS emission flux method (17.1 ± 10.46 Mt year^−1^), were comparable to the emission reported in the CB database (12.95 Mt year^−1^).

### A11 Kawai Thermal Power Project

On 30 January 2017, the emission rate for the Kawai Thermal Power Project was estimated using both the GP model and the CS emission flux method, resulting in estimates of 9.31 ± 3.34 Mt year^−1^ and 12.14 ± 3.61 Mt year^−1^, respectively. These estimates were found to be higher (1.7 and 2.2 times, respectively) than the emission rate provided in the CB database (5.48 Mt year^−1^). It should be noted that the emissions provided in EDGAR and ODIAC within 50 km of the observed plume in the upwind direction were 0.5 and 1 Mt year^−1^, respectively. This suggests that the Kawai Thermal Power Project was not included in either the EDGAR or ODIAC inventories, or its emissions may have been highly underestimated in the CB database.

### A12 Bhilai Steel Power Station

We have found different scaling factors (14.7, 2.54, 3.76, and 16.42) for the Bhilai Steel Power Station from the GP model on four different dates: 7 January 2017, 8 February 2017, 14 February 2019, and 10 February 2020. The CS emission flux aligned well with the estimates from the GP model. According to the EDGAR inventory, there is a significant emission source in the vicinity of observed plume in the upwind direction, amounting to ≈ 58 Mt year^−1^. On the other hand, ODIAC data suggests the presence of a source of around ≈15 Mt year^−1^. All these values were higher than the emission rate provided in the CB database for the Bhilai Steel Power Station, which was 3.17 Mt year^−1^.

## Appendix B Methodology

The example cases illustrate the methodology for selecting cases in the CS emission flux method, as seen in Figure B1, and demonstrate background estimation as depicted in Figure B2a. Additionally, Figure B2a represents background uncertainty, while Figure B2b illustrates CS emission flux estimation.

**Table B1:** Uncertainty From Different Components According to Equation 5 for Cluster 1

Date	Uncertainty (Mt year^−1^)	$\epsilon_{w}$ (Mt year^−1^)	$\epsilon_{b}$ (Mt year^−1^)	$\epsilon_{pr}$ (Mt year^−1^)
23 October 2014	17.66	7.14	14.80	6.45
1 February 2017	33.58	15	25.1	16.6
5 February 2017	30.04	16.1	24.9	5.1
13 January 2021	28.27	8.5	27	2

## Appendix C Power Plants

The power plants considered in this study are listed in Table C1, and they are clustered based on the identified anomalies, as shown in Table C2.

**Table C1:** List of Power Plants Were Analyzed in This Study

S No.	Power plants name (number of time emissions are assessed)
1.	Adityapur Works Power Station (4)
2.	Angul Power Station (2)
3.	Angul Steel Power Station (1)
4.	Anpara Power Station (5)
5.	Anpara‐C Power Station (5)
6.	Anpara‐D Power Station (5)
7.	Bellary Thermal Power Station (5)
8.	Bhilai Steel Power Station (4)
9.	Bokaro Steel City Thermal Power Station (4)
10.	Chandrapur Thermal Power Station (2)
11.	Chandrapura Power Station (4)
12.	Dhariwal Power Station (1)
13.	Dongamahua Captive Power Plant (1)
14.	Durgapur Projects Limited Power Station (3)
15.	Durgapur SAIL Power Station (3)
16.	Durgapur Steel City Power Station (3)
17.	Ghugus Power Station (1)
18.	Jamshedpur Works Power Station (4)
19.	Jojobera Power Plant (4)
20.	JSW Vijayanagar Toranagallu Power Station (3)
21.	Kawai Thermal Power Project (1)
22.	Khaperkheda Power Station (1)
23.	Koradi Thermal Power Station (1)
24.	Mahadev Prasad Super Thermal Power Plant (1)
25.	Mejia Power Station (3)
26.	Mundra Thermal Power Project (1)
27.	Neyveli Thermal Power Station I (1)
28.	Neyveli Thermal Power Station II (1)
29.	Neyveli Zero Power Station (1)
30.	Pegadapalli (Jaipur Mandal) Power Station (5)
31.	Ramagundam Power Station (6)
32.	Ramagundam‐B Power Station (6)
33.	Renusagar Power Station (5)
34.	Rihand Power Station (5)
35.	Santaldih Thermal Power Station (1)
36.	Sasan Ultra Mega Power Project (4)
37.	Singrauli Super Thermal Power Station (5)
38.	Sipat Power Station (1)
39.	Talcher Kaniha Super Thermal Power Station (2)
40.	Tamnar Power Station (1)
41.	Tata Mundra Ultra Mega Power Project (1)
42.	Vindhyachal Power Station (4)

**Table C2:** Power Plant Clusters and Their Corresponding List of Power Plants

Cluster no.	Power plants name
Cluster 1	Sasan Ultra Mega Coal Power Plant Vindhyachal STPS Coal Power Plant Singrauli Super Coal Power Plant Rihand Coal Power Plant Renusagar Power Station Anpara Coal Power Plant Anpara‐C Power Station Anpara‐D Power Station
Cluster 2	Ramagundam Power Station Ramagundam B (RTS‐B) Coal Power Station Pegadapalli Power Station
Cluster 3	Chandrapur Thermal Power Station Dhariwal Power Station Ghugus Power Station
Cluster 4	Angul Power Station Talcher Kaniha Super Thermal Power Station Angul Steel Power Station
Cluster 5	Tata Mundra Ultra Mega Power Project The Mundra Thermal Power Project
Cluster 6	Dongamahua Captive Power Plant Tamnar Power Station
Cluster 7	Khaperkheda Power Station and Koradi Thermal Power Station
Cluster 8	Durgapur Steel City Durgapur SAIL Power Station Durgapur Projects Limited Power Station Mejia Power Station
Cluster 9	Jojobera Power Plant Jamshedpur Works Power Station, Adityapur Works Power Station Mahadev Prasad Super Thermal Power Plant
Cluster 10	Bokaro Steel City Thermal Power Station Chandrapura Power Station Santaldih Thermal Power Station
Cluster 11	Bellary Thermal Power Station JSW Vijayanagar Toranagallu Power Station
Cluster 12	Neyveli Thermal Power Station I Neyveli Thermal Power Station II Neyveli Zero Power Station
