# Supplementary material for: Fossil Fuel CO2 Emission Signatures Over India Captured by OCO‐2 Satellite Measurements
Source: Earths Future. 2024 Nov 22;12(11):e2023EF004411. doi: 10.1029/2023EF004411 (PMC11583129; doi:10.1029/2023EF004411)

Supporting Information for “Fossil fuel CO_2 emission signatures over India captured by OCO-2 satellite measurements”

Vigneshkumar Balamurugan[1], Jia Chen[1]

[1]Environmental Sensing and Modeling, Technical University of Munich (TUM), Munich, Germany

Contents of this file:

Table S1

Table S2

Table S1: The dates and locations of identified anomalies, reported emissions in the CB database, estimated emissions using the GP model and CS flux method, and emissions reported in EDGAR, ODIAC and CAMS biomass data for power plant cases.

S No	Date (anomaly location: latitude, longitude)	Power Plants Name	Reported Emission in the CB database (in Mt Year^{-1})	Emission estimate using GP model (in Mt Year^{-1})	Emission estimate using c/s emission flux method (in Mt Year^{-1})	EDGAR emission within 50 km upwind (in Mt year^{-1})	ODIAC emission within 50 km upwind (in Mt year^{-1})	Biomass burning emission within 50 km upwind (in Mt year^{-1})
1.	23 October 2014<br><br>(23.95, 82.6)	Sasan Ultra Mega Power Project<br>Vindhyachal Power Station<br>Singrauli Super Thermal Power Station<br>Rihand Power Station<br>Anpara Power Station<br>Anpara-C Power Station<br>Renusagar Power Station	84.14	64.78±17.66	-	101.5616	108.8215	0
2.	1 February 2017<br><br>(23.96, 82.62)	Sasan Ultra Mega Power Project<br>Vindhyachal Power Station<br>Singrauli Super Thermal Power Station<br>Rihand Power Station<br>Anpara Power Station<br>Anpara-C Power Station<br>Anpara-D Power Station<br>Renusagar Power Station	88.37	114.8±33.58	-	103.8266	118.5322	0

3.	5 March 2017<br><br>(23.98, 82.62)	Sasan Ultra Mega Power Project<br>Vindhyachal Power Station<br>Singrauli Super Thermal Power Station<br>Rihand Power Station<br>Anpara Power Station<br>Anpara-C Power Station<br>Anpara-D Power Station<br>Renusagar Power Station	88.37	99.85±30.04	-	103.8266	129.5251	0
4.	13 January 2021<br><br>(23.92, 83.12)	Sasan Ultra Mega Power Project<br>Vindhyachal Power Station<br>Singrauli Super Thermal Power Station<br>Rihand Power Station<br>Anpara Power Station<br>Anpara-C Power Station<br>Anpara-D Power Station<br>Renusagar Power Station	88.37	91.02±28.27	75.32±27.4	74.6588	164.7294	0
5.	10 November 2014<br><br>(24, 82.71)	Singrauli Super Thermal Power Station<br>Rihand Power Station<br>Anpara Power Station<br>Anpara-C Power Station<br>Renusagar Power Station	44.93	74.13±24.71	-	62.4616	54.6805	0.1802
6.	16 January 2015<br><br>(18.6, 79.34)	Ramagundam-B Power Station<br>Ramagundam Power Station	14.33	14.61±6.7	18.41±5.9	21.5016	40.4657	0.0526
7.	20 December 2016<br><br>(18.6, 79.23)	Ramagundam-B Power Station<br>Ramagundam Power Station<br>Pegadapalli (Jaipur Mandal) Power Station	19.49	10.71±3.89	7.92±4.31	20.7014	43.4112	0.0212

8.	5 November 2017<br><br>(18.62, 79.24)	Ramagundam-B Power Station<br>Ramagundam Power Station<br>Pegadapalli (Jaipur Mandal) Power Station	19.49	23.19±12.86	29.6±12.85	22.0493	42.6319	0.0324
9.	7 December 2017<br><br>(18.65, 79.2)	Ramagundam-B Power Station<br>Ramagundam Power Station<br>Pegadapalli (Jaipur Mandal) Power Station	19.49	20.26±15.6	19.32±12.01	23.3059	45.2223	0
10.	26 November 2018<br><br>(18.65, 79.33)	Ramagundam-B Power Station<br>Ramagundam Power Station<br>Pegadapalli (Jaipur Mandal) Power Station	19.49	24.16±11.69	24.9±13.4	23.6754	44.5287	0.1235
11.	15 February 2020<br><br>(18.72, 79.33)	Ramagundam-B Power Station<br>Ramagundam Power Station<br>Pegadapalli (Jaipur Mandal) Power Station	19.49	18.9±4.1	25.2±5.36	22.2271	46.7727	0
12.	16 January 2015<br>(20, 79)	Chandrapur Thermal Power Station<br>Dhariwal power station<br>Ghugus power station	20.19	12.31±9.08	13.4±8.8	29.7422	40.0671	0
13.	22 December 2016<br>(19.93, 79.27)	Chandrapur Thermal Power Station	14.42	12.97±8.65	7.3±6.2	25.3036	41.7171	0
14.	25 January 2017<br>(21.05, 84.86)	Angul Power Station<br>Talcher Kaniha Super Thermal Power Station	19.54	23.83±10.94	-	44.3551	69.3914	1.4008

15.	15 November 2019 (20.8, 84.88)	Angul Power Station<br>Talcher Kaniha Super Thermal Power Station<br>Angul Steel Power Station	23.72	10.43±4.74	-	16.4265	73.3165	1.5494
16.	1 March 2018 (22.22, 82.48)	Sipat Power Station	12.95	14.24±8.54	17.13±10.46	25.6729	13.5886	0.2531
17.	3 October 2018 (22.82, 69.6)	Tata Mundra Ultra Mega Power Project<br>Mundra Thermal Power Project (Adani)	37.07	51.89±12.22	57.04±14.92	2.997	1.268	0
18.	13 January 2021 (22.16, 83.68)	Dongamahua Captive Power Plant<br>Tamnar Power Station	18.65	26.29±15.85	25.44±15.43	38.8885	3.2841	0.3622
19.	10 January 2018 (21.21, 79.22)	Khaperkheda power station<br>Koradi Thermal Power Station	17.76	10.65±6.74	-	23.9697	36.7688	0.1410
20.	19 November 2014 (23.3, 87.56	Durgapur Steel City Andal power station<br>Durgapur SAIL power station<br>Durgapur Projects Limited power station<br>Mejia power station	20.98	56.77±12.98	-	53.8409	19.6708	0
21.	16 March 2017 (23.52, 87.4)	Durgapur Steel City Andal power station<br>Durgapur SAIL power station<br>Durgapur Projects Limited power station<br>Mejia power station	20.98	71.33±36.71	-	76.0030	25.0810	0

22.	29 December 2017 (23.35, 87.44)	Durgapur Steel City Andal power station<br>Durgapur SAIL power station<br>Durgapur Projects Limited power station<br>Mejia power station	20.98	55.38±19.3	-	61.8673	23.0727	0.1210
23.	18 January 2017 (22.62, 86.04)	Jojobera Power Plant<br>Jamshedpur Works power station<br>Adityapur works power station	5.2	43.26±17.16	53.06±9.12	41.4552	9.4802	0.1245
24.	28 November 2020 (22.5, 86.14)	Jojobera Power Plant<br>Jamshedpur Works power station<br>Adityapur works power station	5.2	42.38±14.35	41.5±32.75	34.1113	8.0214	0.0979
25.	31 October 2022 (22.64, 86.11)	Jojobera Power Plant<br>Jamshedpur Works power station<br>Adityapur works power station	5.2	27.04±15.88	36.5±28.4	45.9448	9.7384	0
26.	7 January 2018 (22.74, 86.46)	Jojobera Power Plant<br>Jamshedpur Works power station<br>Adityapur works power station<br>Mahadev Prasad Super Thermal Power Plant	7.98	44.04±33.51	46.46±40.7	46.3038	10.1899	0.0715
27.	30 December 2014 (23.58, 86.05)	Bokaro Steel City Thermal Power Station<br>Chandrapura power station	7.47	19.79±12.69	11.49±7.11	70.8386	8.6428	0.7196
28.	31 January 2015 (23.7, 86.54)	Bokaro Steel City Thermal Power Station<br>Chandrapura power station	7.47	41.08±16.95	-	66.6614	4.8155	0.4131

29.	18 January 2022 (23.66, 86.44)	Bokaro Steel City Thermal Power Station<br>Chandrapura power station	7.47	47.73±22.93	47.67±9.32	80.3754	45.4088	0.7404
30.	31 January 2021 (23.57, 86.58)	Bokaro Steel City Thermal Power Station<br>Chandrapura power station<br>Santaldih Thermal Power Station	10.11	61.67±32.35	60.3±38.8	91.3430	11.7864	0.4334
31.	7 January 2017 (21.58, 81.66)	Bhilai Steel power station	3.17	46.59±31.35	46.89±36.1	58.6355	2.9587	0.0309
32.	8 February 2017 (21.26, 81.74)	Bhilai Steel power station	3.17	8.05±4.88	8.24±6.8	56.8281	13.4112	0.1603
33.	14 February 2019 (21.46, 81.62)	Bhilai Steel power station	3.17	11.91±11.09	13.93±12.7	58.6355	12.3757	0.0216
34.	10 February 2020 (21.15, 81.2)	Bhilai Steel power station	3.17	52.05±31.38	66.5±42.72	54.6881	14.7715	0.0387
35.	4 March 2018 (15.22, 76.54)	Bellary Thermal Power Station<br>JSW Vijayanagar Toranagallu power station	12.08	17.87±1.38	22.84±5.5	30.2110	7.4953	0.0440

36.	18 January 2019 (14.9, 76.21)	Bellary Thermal Power Station<br>JSW Vijayanagar Toranagallu power station	12.08	68.85±15.82	64.6±6.7	32.7618	8.2091	0
37.	24 February 2021 (15.19, 76.54)	Bellary Thermal Power Station<br>JSW Vijayanagar Toranagallu power station	12.08	35.87±4.1	43.45±6.2	32.0224	7.5785	0.0807 (0.0807, 0.0807)
38.	24 February 2015<br><br>(11.45, 79.3)	Neyveli Thermal Power Station I<br>Neyveli Thermal Power Station II<br>Neyveli Zero power station	19.36	37.55±9.29	38.8±12.15	36.0196	41.1005	0
39.	30 January 2017 (24.68, 76.94)	Kawai Thermal Power Project	5.48	9.31±3.3	12.14±3.61	0.5225	1.0355	0

Table S2: The dates and locations of identified anomalies, estimated emissions using the CS flux method, and emissions reported in EDGAR, ODIAC and CAMS biomass data for non-power plant cases.

S No	Date (anomaly location: latitude, longitude)	Emission estimate using CS emission flux method (in Mt Year^{-1})	EDGAR emission within 50 km upwind (in Mt year^{-1})	ODIAC emission within 50 km upwind (in Mt year^{-1})	Biomass burning emission within 50 km upwind (in Mt year^{-1})
1.	12 January 2016 (27, 75.98)	40.14±17.38	3.13	13.86	0
2.	25 November 2017 (21.36,81.72)	41.59±33.49	3.73	2.37	0.05
3.	19 January 2018 (21.76,83.21)	11.18±6.56	2.30	2.04	0.95
4.	29 January 2018 (15.35,75.36)	46.96 ± 6.18	1.23	1.41	0
5.	22 November 2018 (22.50,72.06)	20.88 ± 12.08	0.54	0.69	0.51
6.	04 January 2019 (19.12,77.96)	19.05 ± 12.82	0.35	1.93	0
7.	6 January 2019 (22.04,82.94)	15.27 ± 9.63	1.58	2.89	0.23

8.	28 February 2019 (28.22,76.92)	67.58 ± 38.91	2.43	6.47	0
9.	27 March 2019 (23.72,80.69)	7.50 ± 3.53	1.22	1.17	0
10.	24 November 2019 (21.05,83.38)	4.68 ± 3.39	0.60	0.35	0
11.	24 November 2019 (21.86,83.18)	4.72 ± 2.50	3.63	2.08	0
12.	10 February 2020 (24.48,80.30)	5.66 ± 1.96	0.38	0.20	0
13.	2 December 2020 (22.36,70.71)	32.10 ± 23.89	0.95	4.37	0
14.	10 December 2020 (21.00,83.36)	8.47 ± 6.25	0.59	0.37	0.02
15.	8 March 2021 (22.18,70.68)	40.18 ± 25.13	0.37	1.94	0
16.	29 November 2021 (21.05,83.46)	14.10 ± 11.75	0.67	0.40	0
17.	23 December 2022 (21.50,81.68)	40.86 ± 26.66	6.83	3.50	0.03

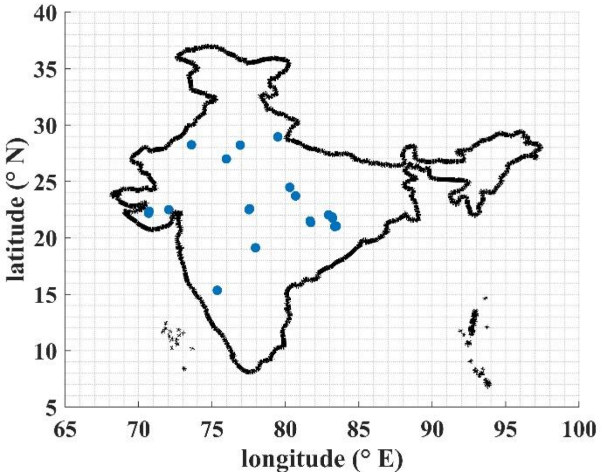

Figure S1. Locations of the CS flux method applied for non-power plant cases.

Figures similar to Figure 3 for all analyzed cases are given below.

Cluster-1 on “1 February 2017”

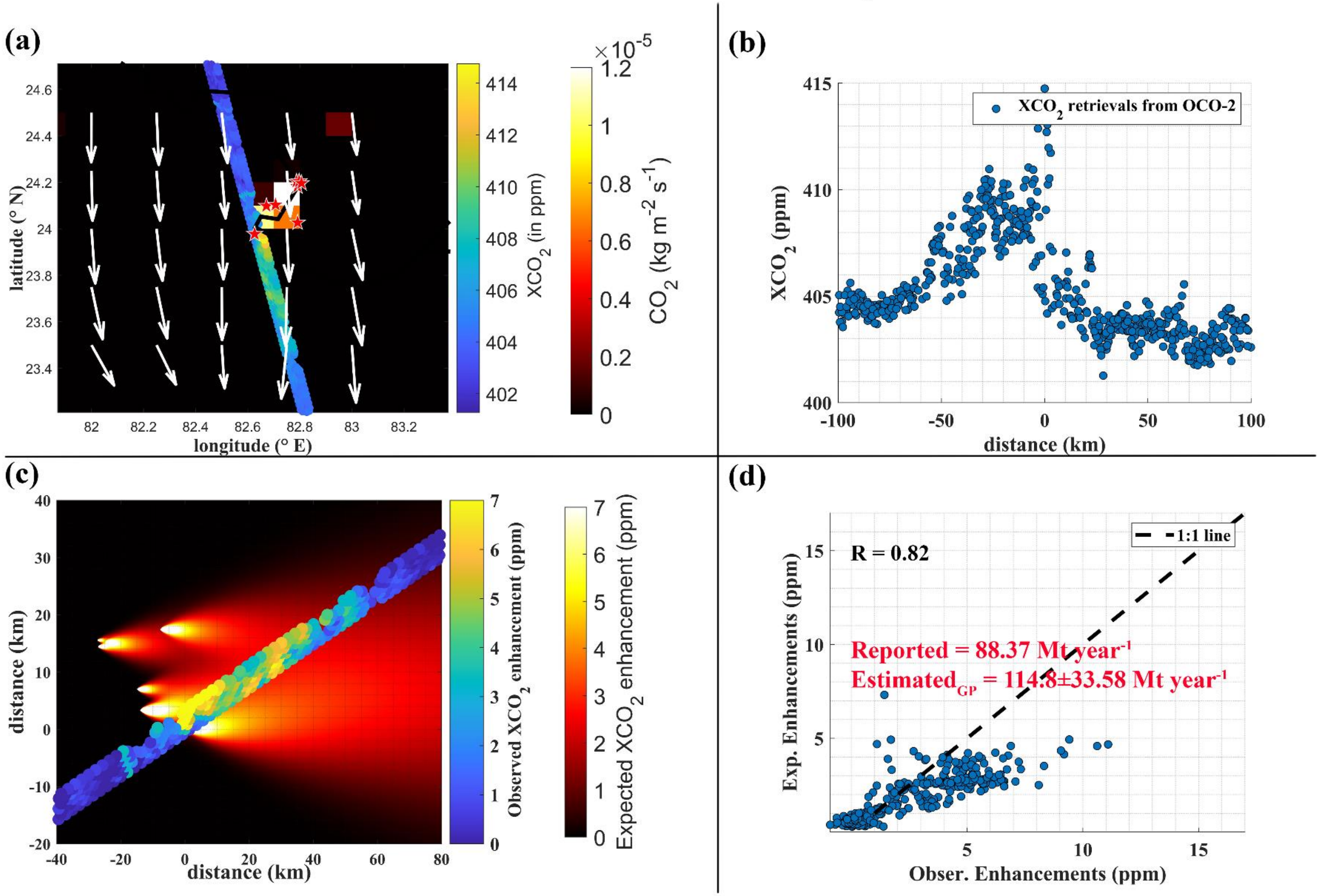

Cluster-1 on "5 March 2017"

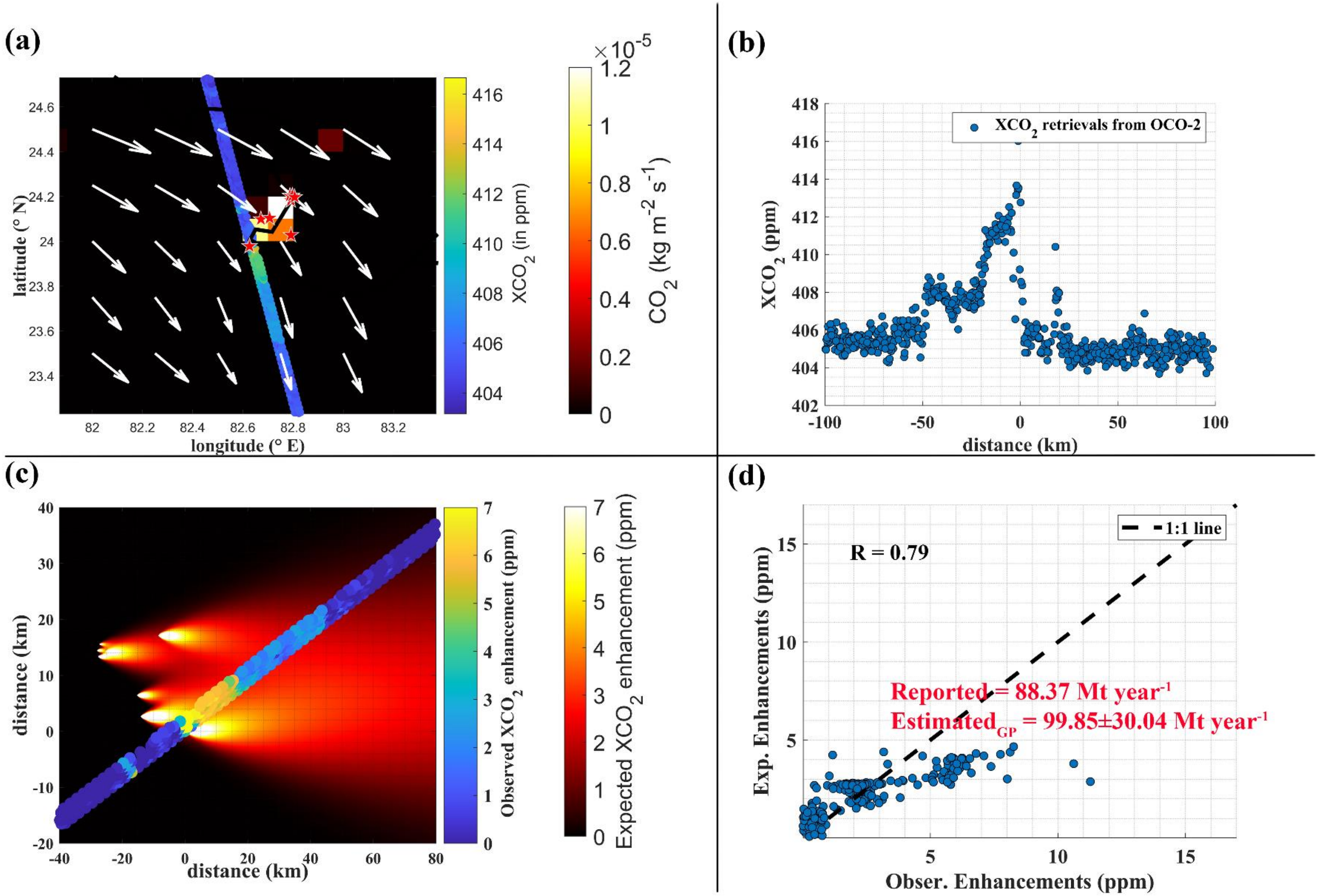

Cluster-1 on "13 January 2021"

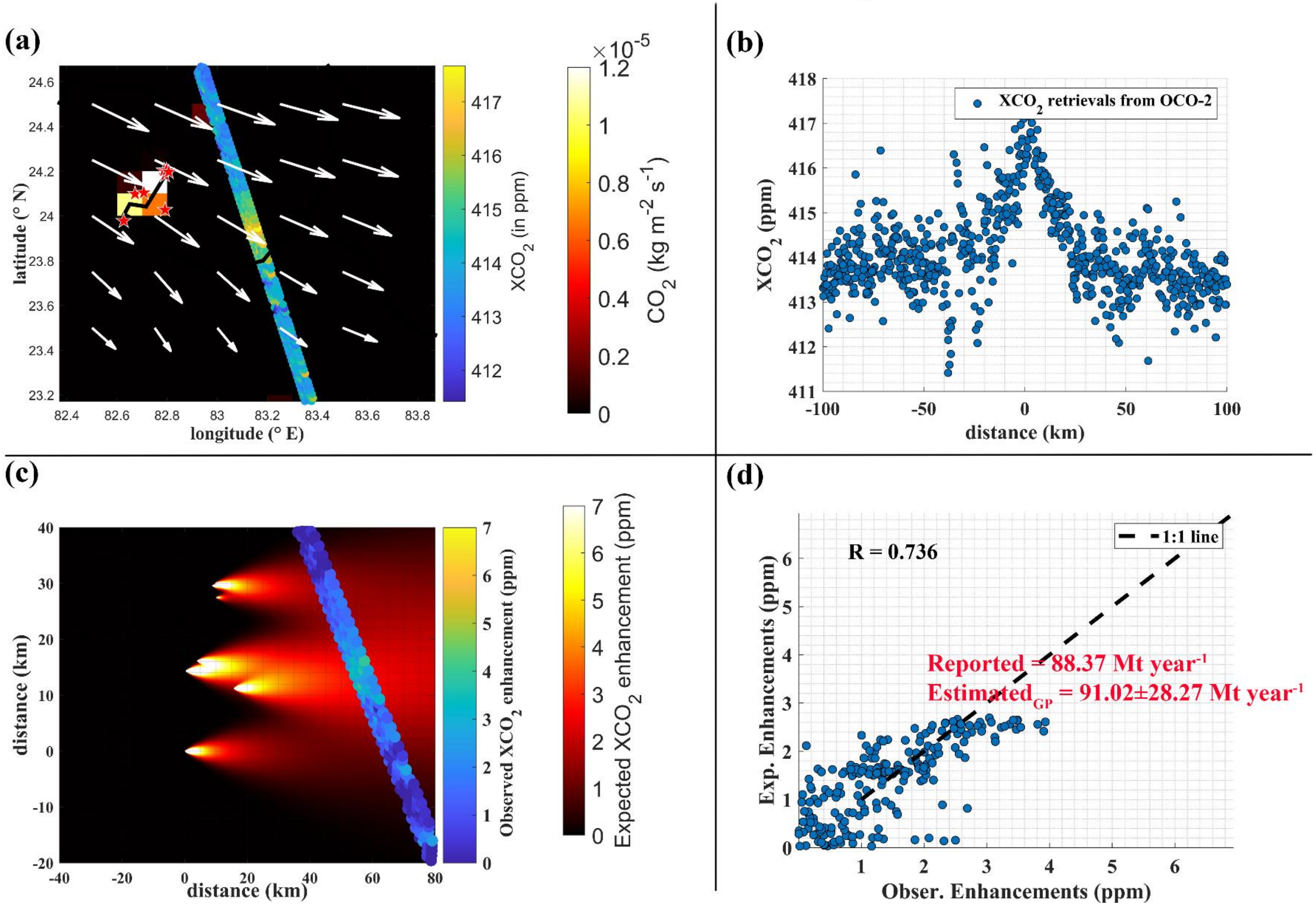

Cluster-1 on "11 November 2014"

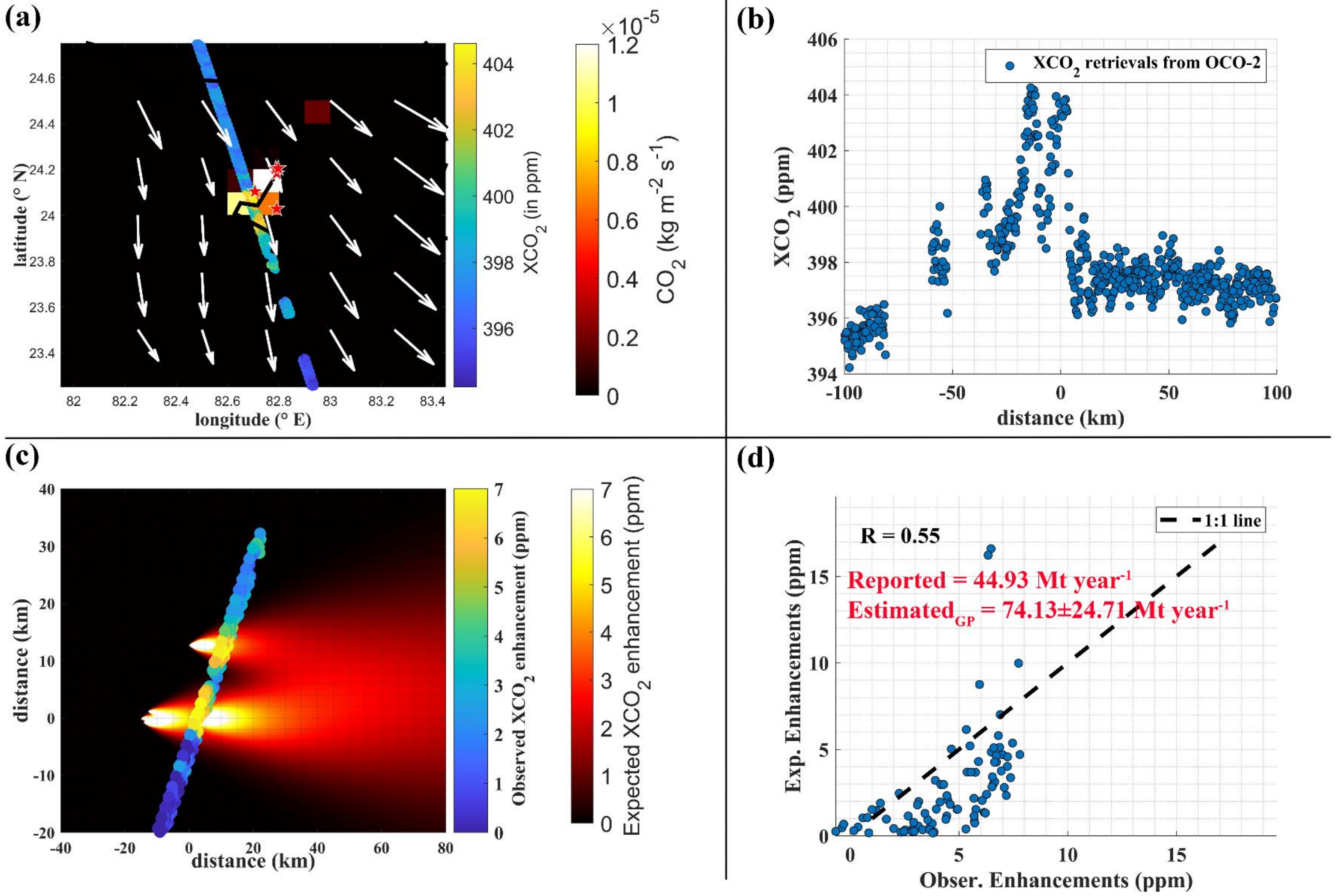

Cluster-2 on "16 January 2015"

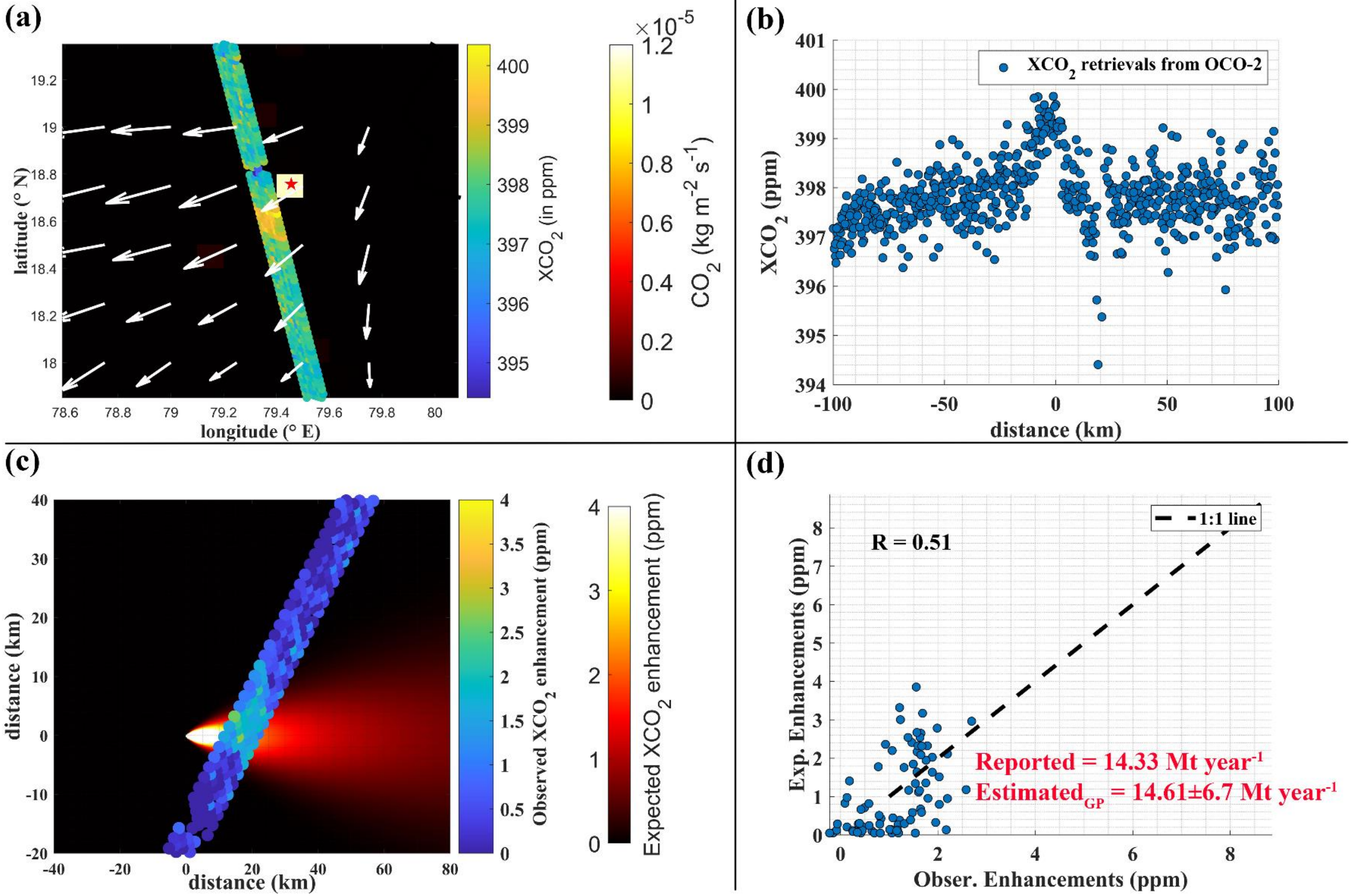

Cluster-2 on "20 December 2016"

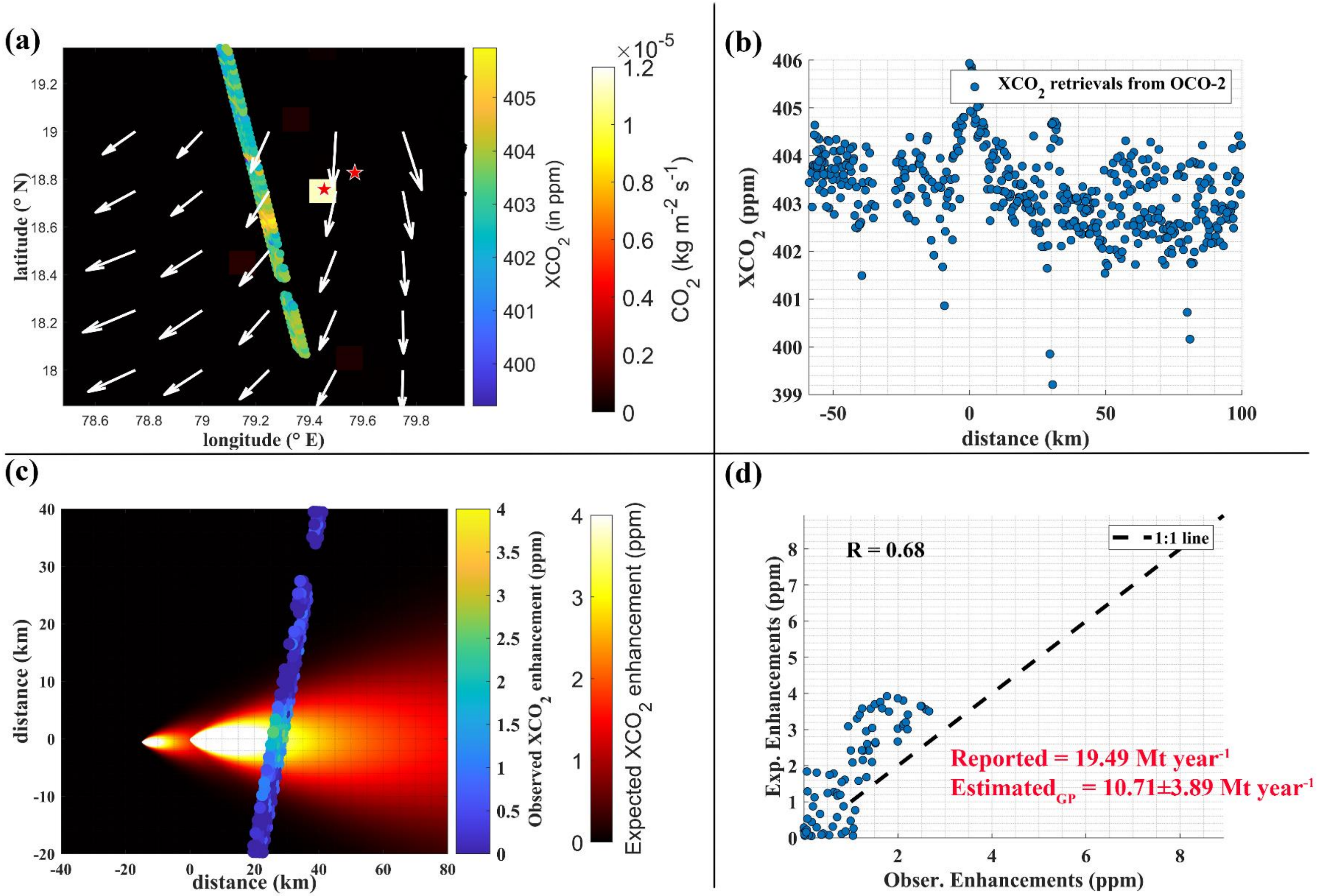

Cluster-2 on "5 November 2017"

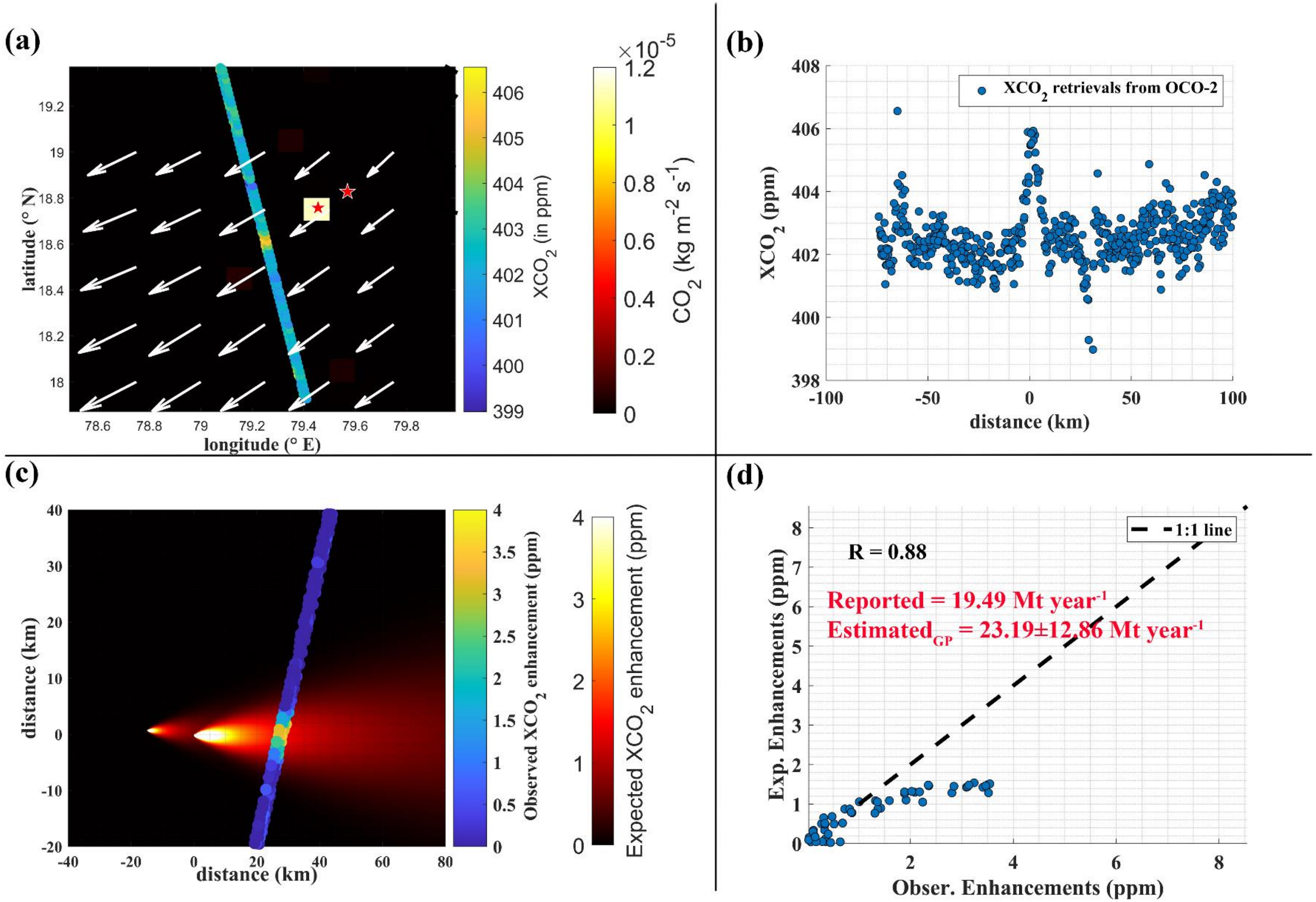

Cluster-2 on "7 December 2017"

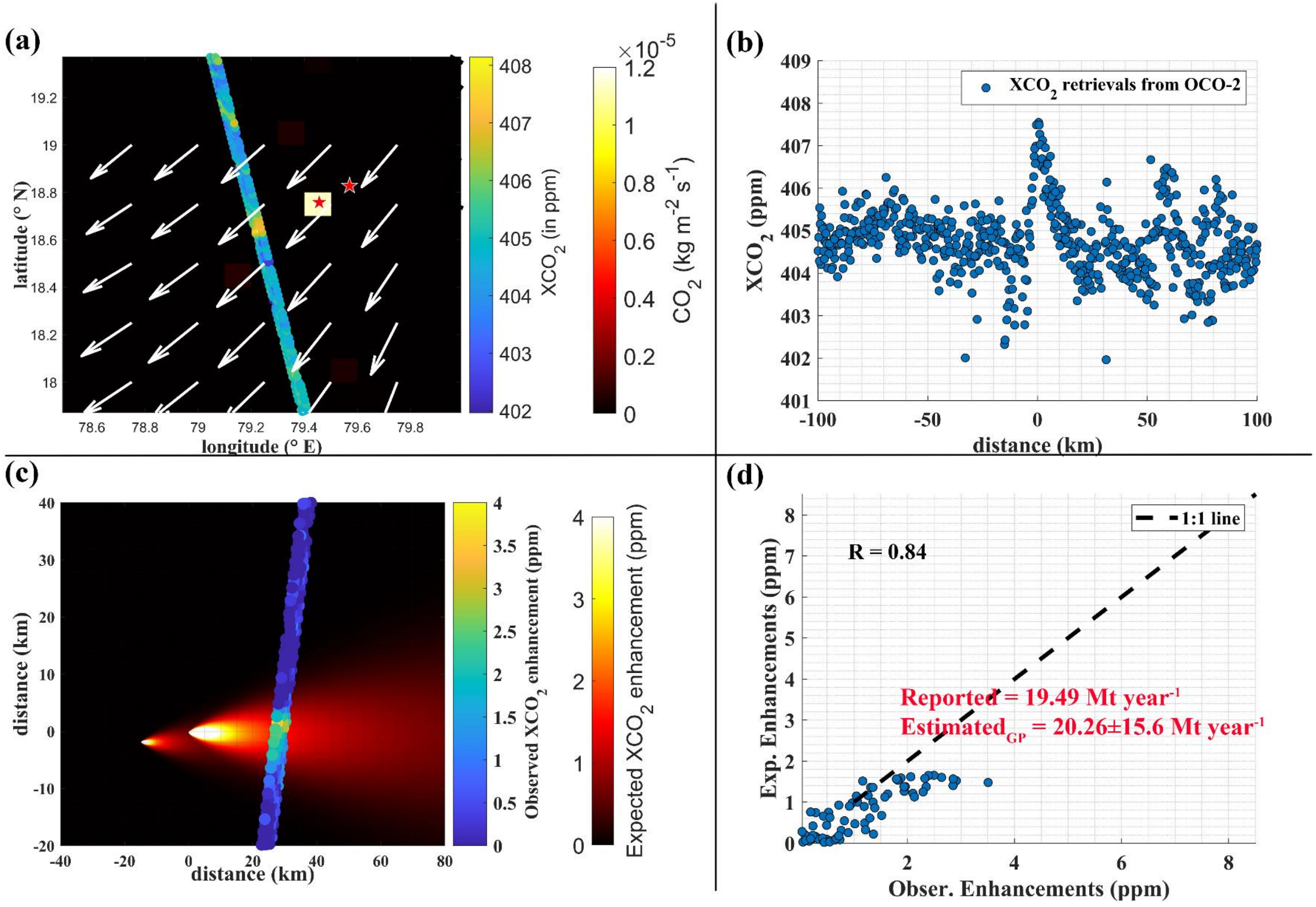

Cluster-2 on "26 November 2018"

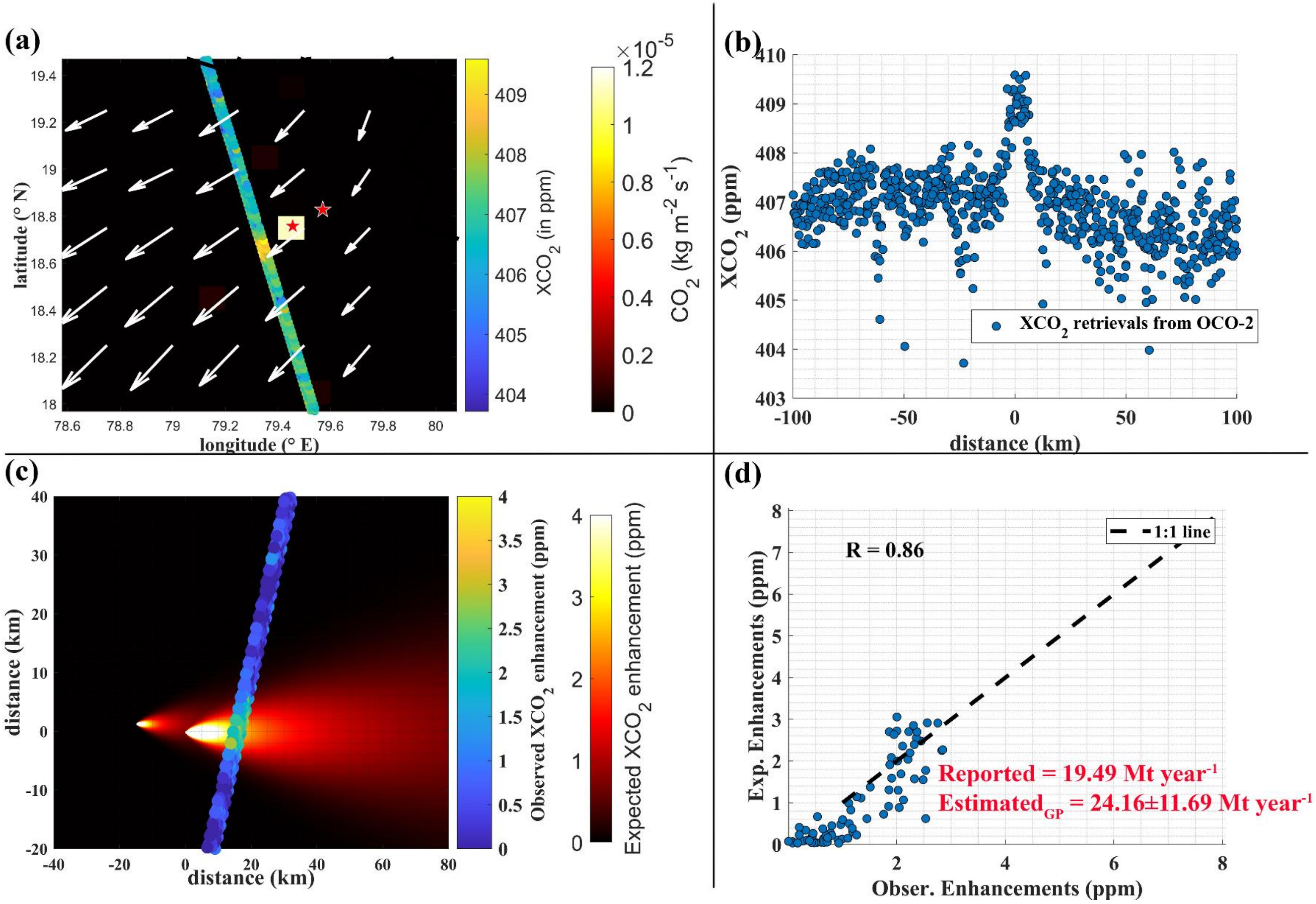

Cluster-2 on "15 February 2020"

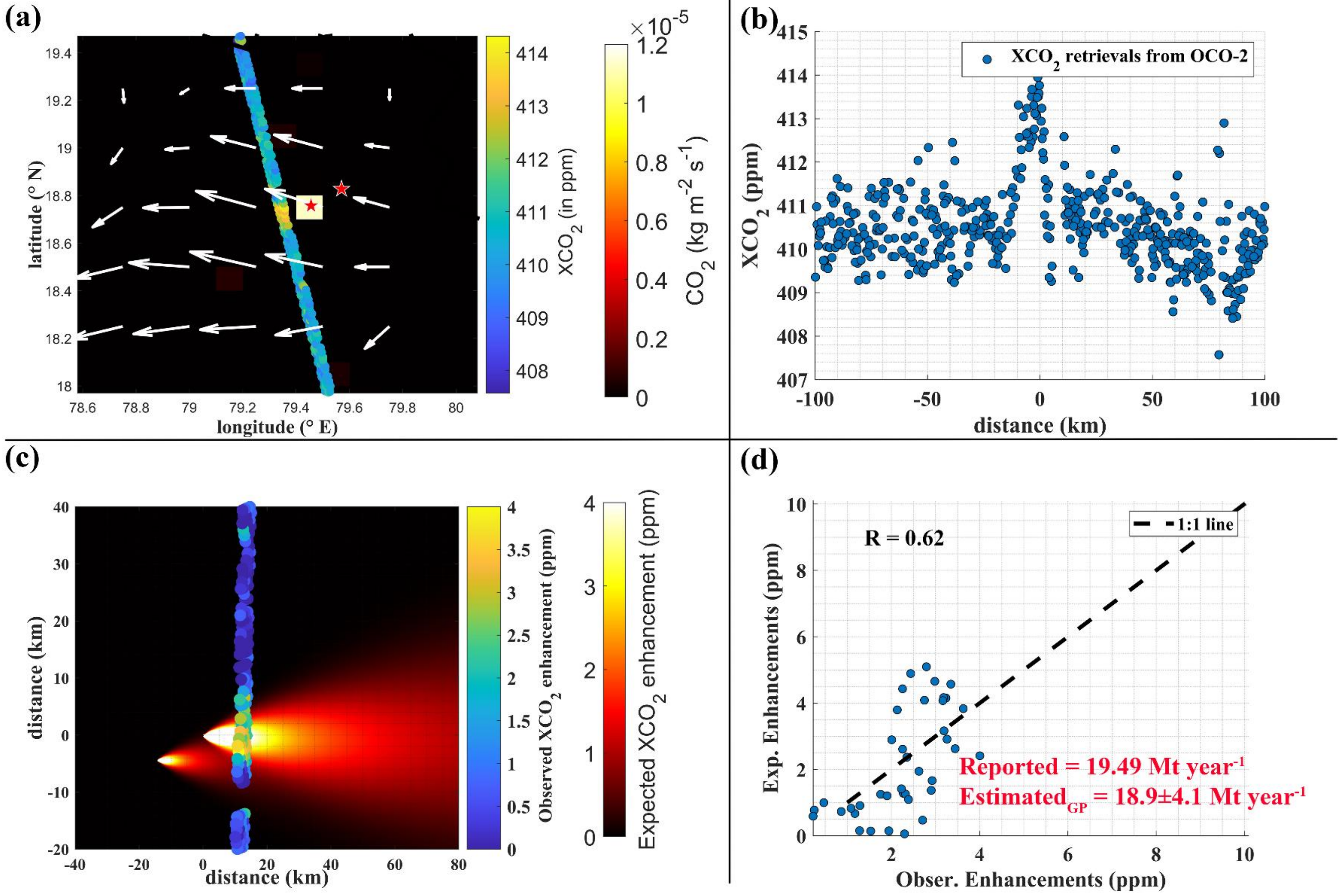

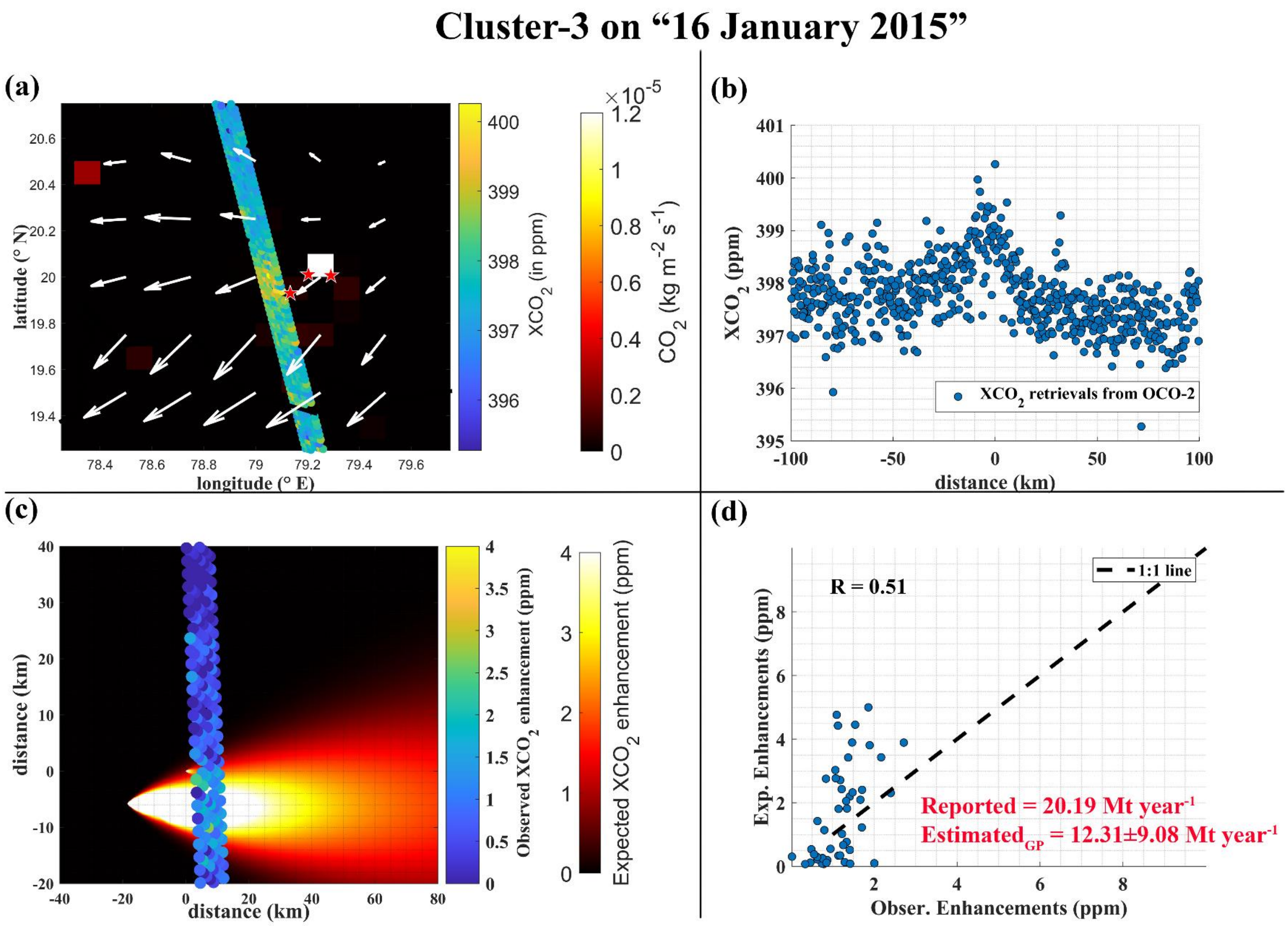

Cluster-3 on "16 January 2015"
(a)
latitude (° N)
longitude (° E)
XCO2 (in ppm)
CO2 (kg m-2 s-1)
×10-5
(b)
XCO2 (ppm)
distance (km)
XCO2 retrievals from OCO-2
(c)
distance (km)
Observed XCO2 enhancement (ppm)
Expected XCO2 enhancement (ppm)
(d)
R = 0.51
1:1 line
Exp. Enhancements (ppm)
Obser. Enhancements (ppm)
Reported = 20.19 Mt year-1
EstimatedGP = 12.31±9.08 Mt year-1

Cluster-3 on "22 December 2016"

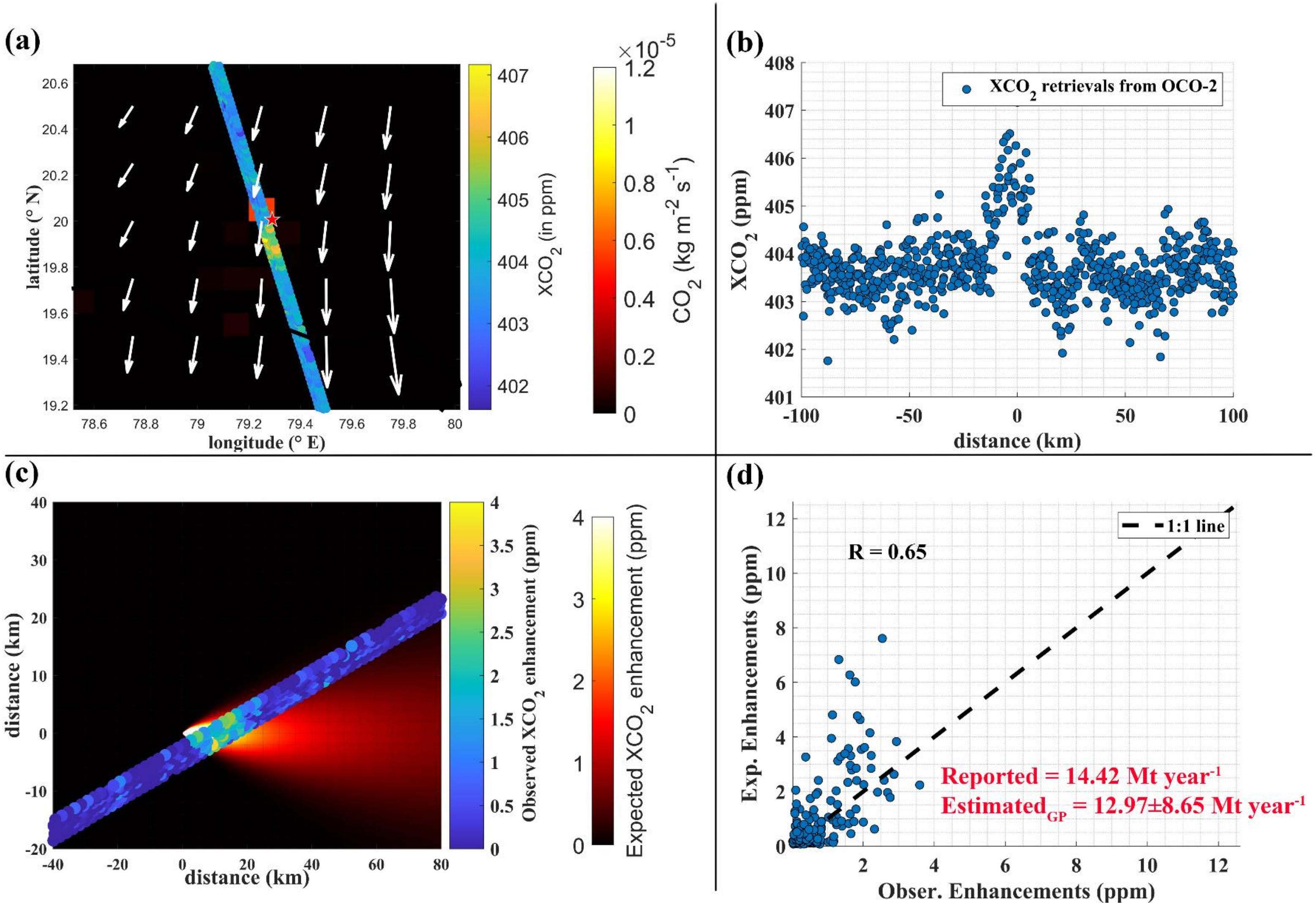

Cluster-4 on "25 January 2017"

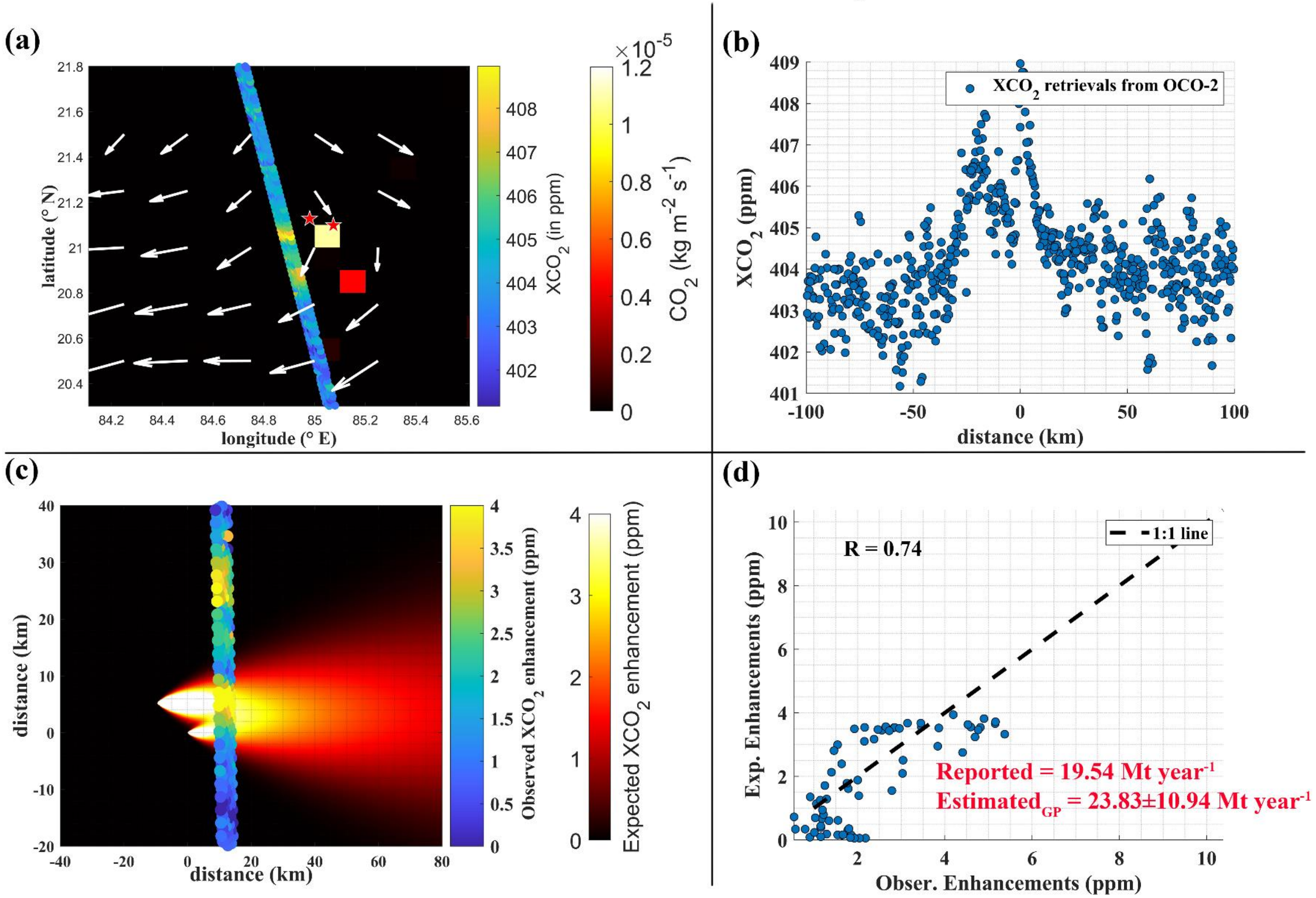

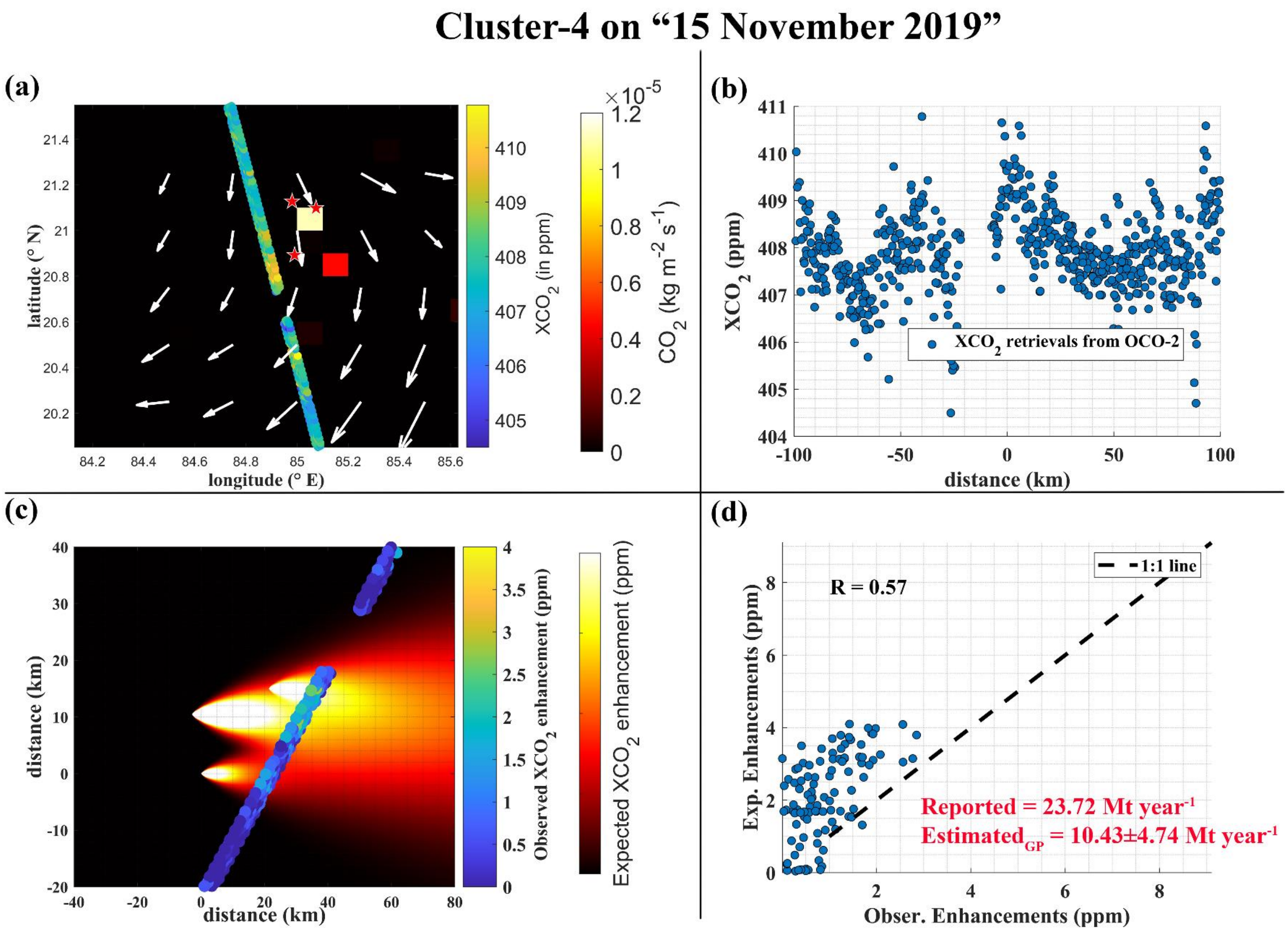
Cluster-4 on "15 November 2019"
(a)
latitude (° N)
longitude (° E)
XCO2 (in ppm)
CO2 (kg m-2 s-1)
×10-5
(b)
XCO2 (ppm)
distance (km)
XCO2 retrievals from OCO-2
(c)
distance (km)
distance (km)
Observed XCO2 enhancement (ppm)
Expected XCO2 enhancement (ppm)
(d)
R = 0.57
1:1 line
Exp. Enhancements (ppm)
Obser. Enhancements (ppm)
Reported = 23.72 Mt year-1
EstimatedGP = 10.43±4.74 Mt year-1

Sipat Power Station on "1 March 2018"

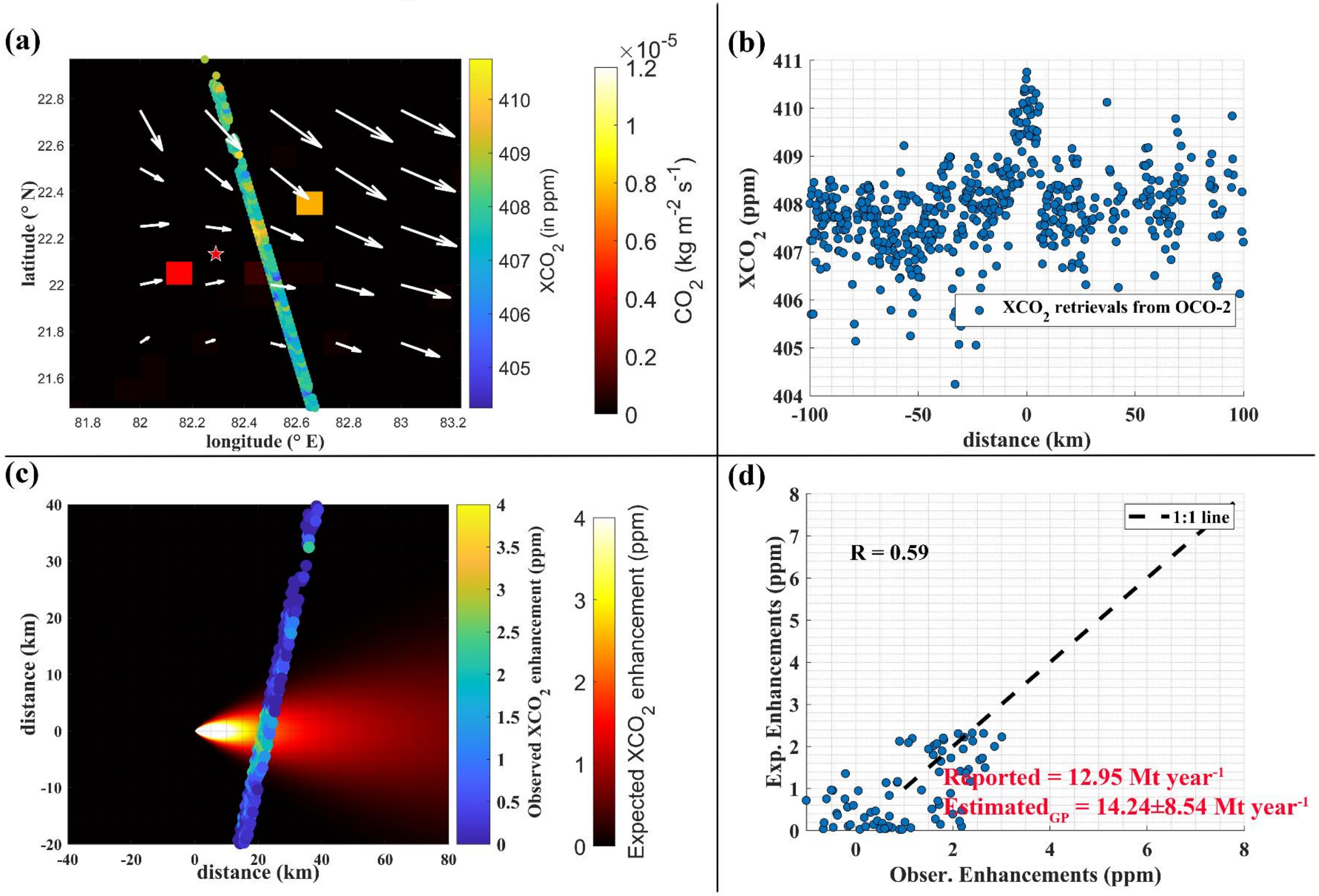

Cluster-6 on “13 January 2021”

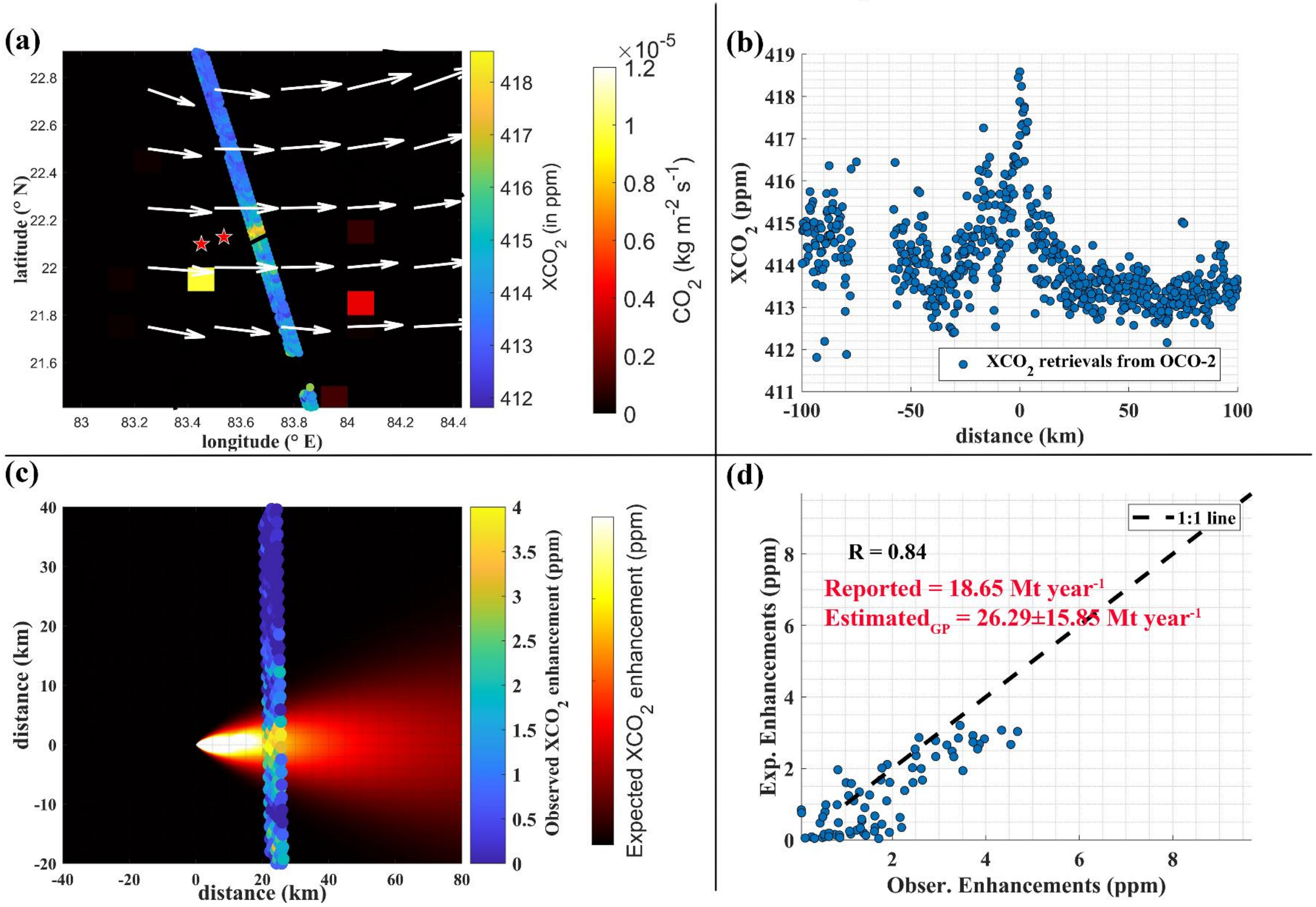

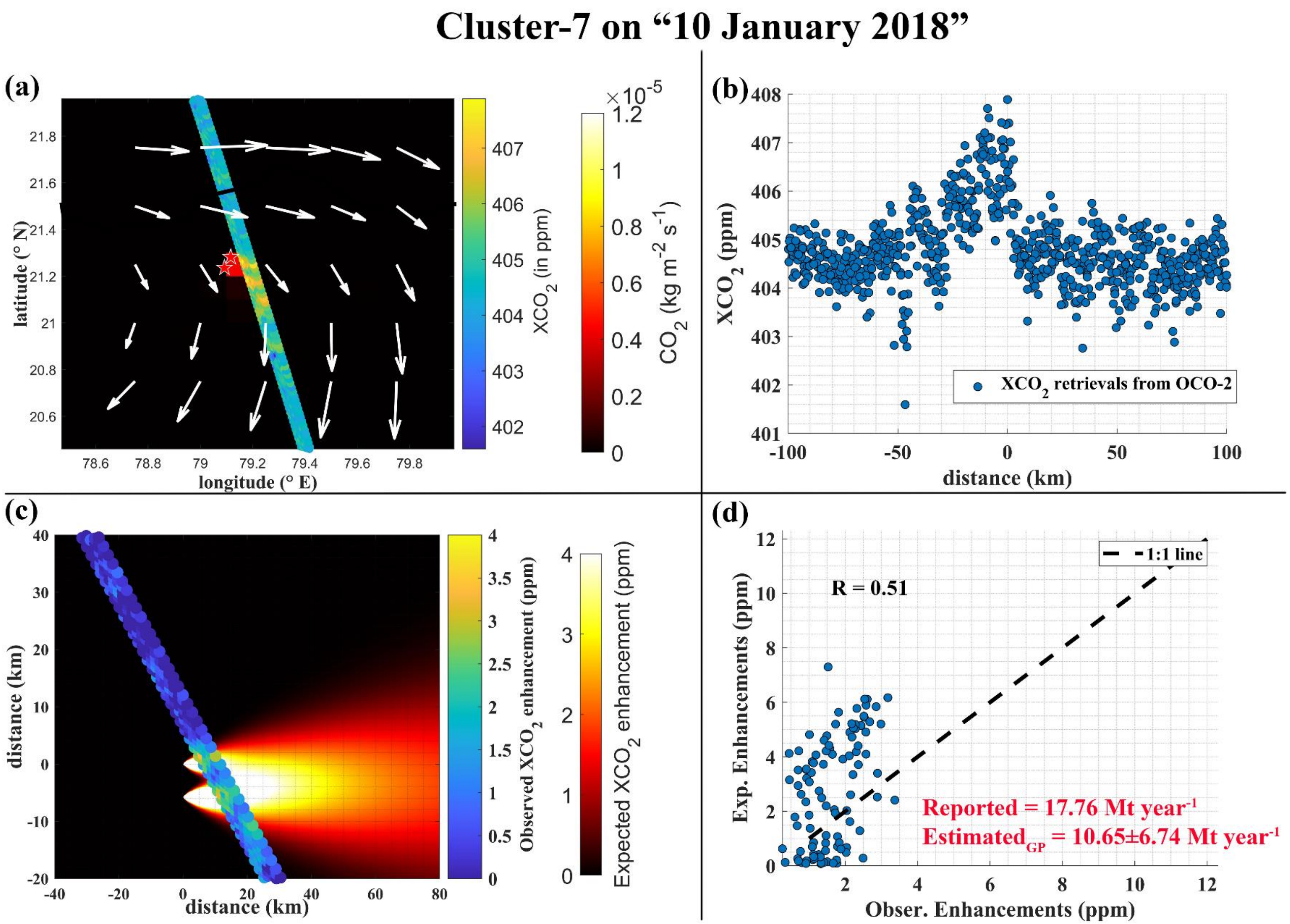
Cluster-7 on "10 January 2018"
(a)
latitude (° N)
longitude (° E)
XCO2 (in ppm)
CO2 (kg m-2 s-1)
×10-5
(b)
XCO2 (ppm)
distance (km)
XCO2 retrievals from OCO-2
(c)
distance (km)
Observed XCO2 enhancement (ppm)
Expected XCO2 enhancement (ppm)
(d)
Exp. Enhancements (ppm)
Obser. Enhancements (ppm)
1:1 line
R = 0.51
Reported = 17.76 Mt year-1
EstimatedGP = 10.65±6.74 Mt year-1

Cluster-8 on "19 November 2014"

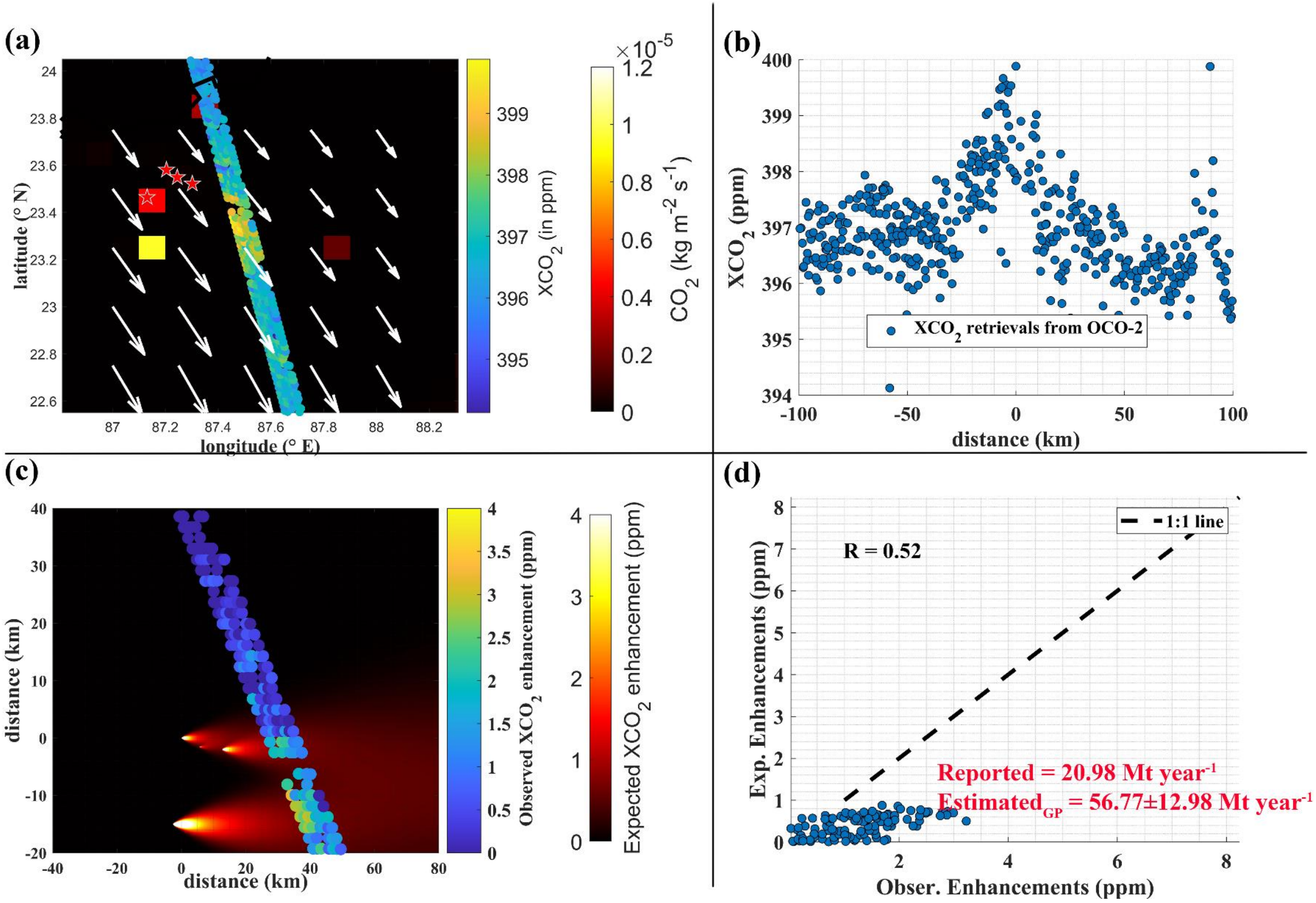

Cluster-8 on “16 March 2017”

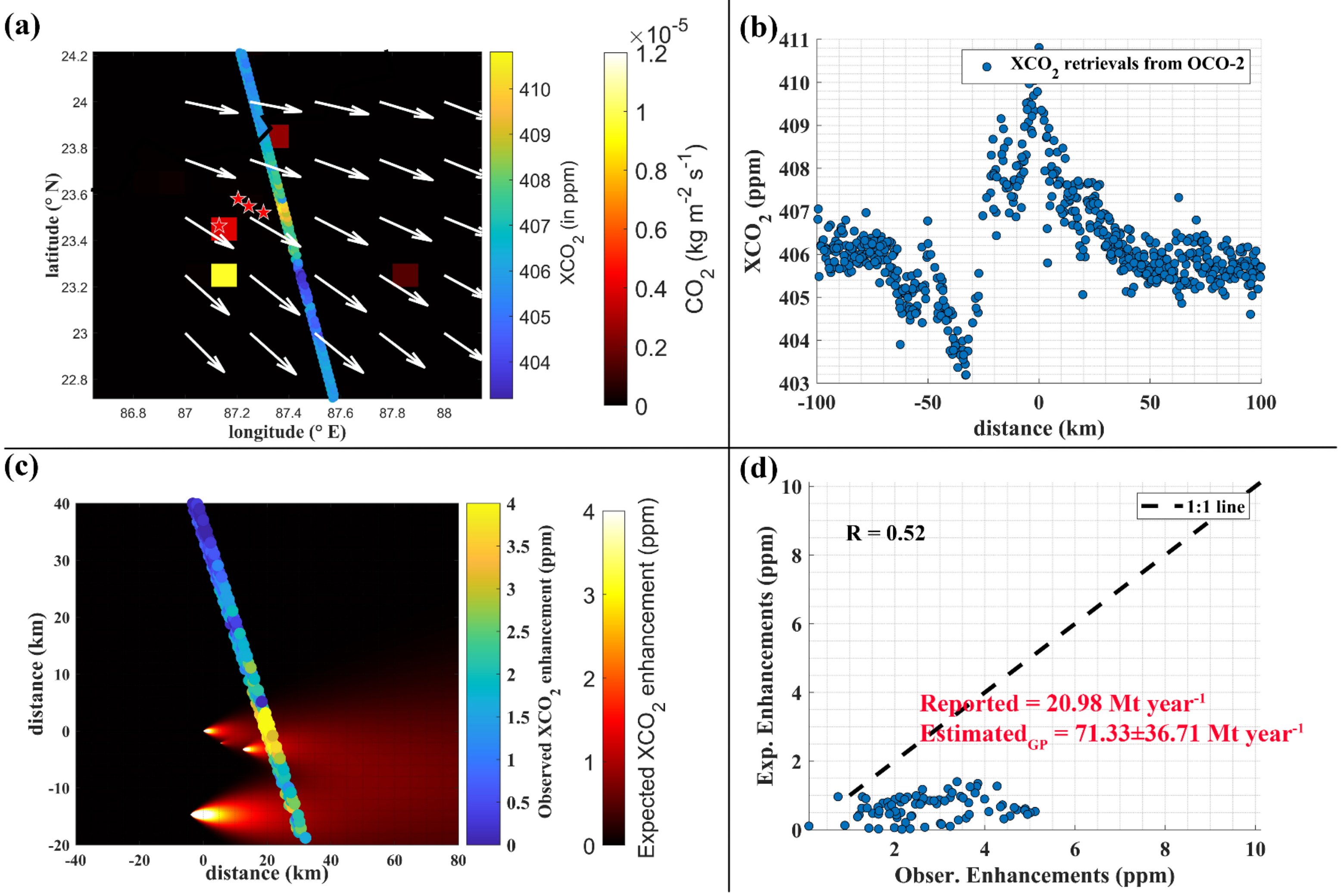

Cluster-8 on "29 December 2017"

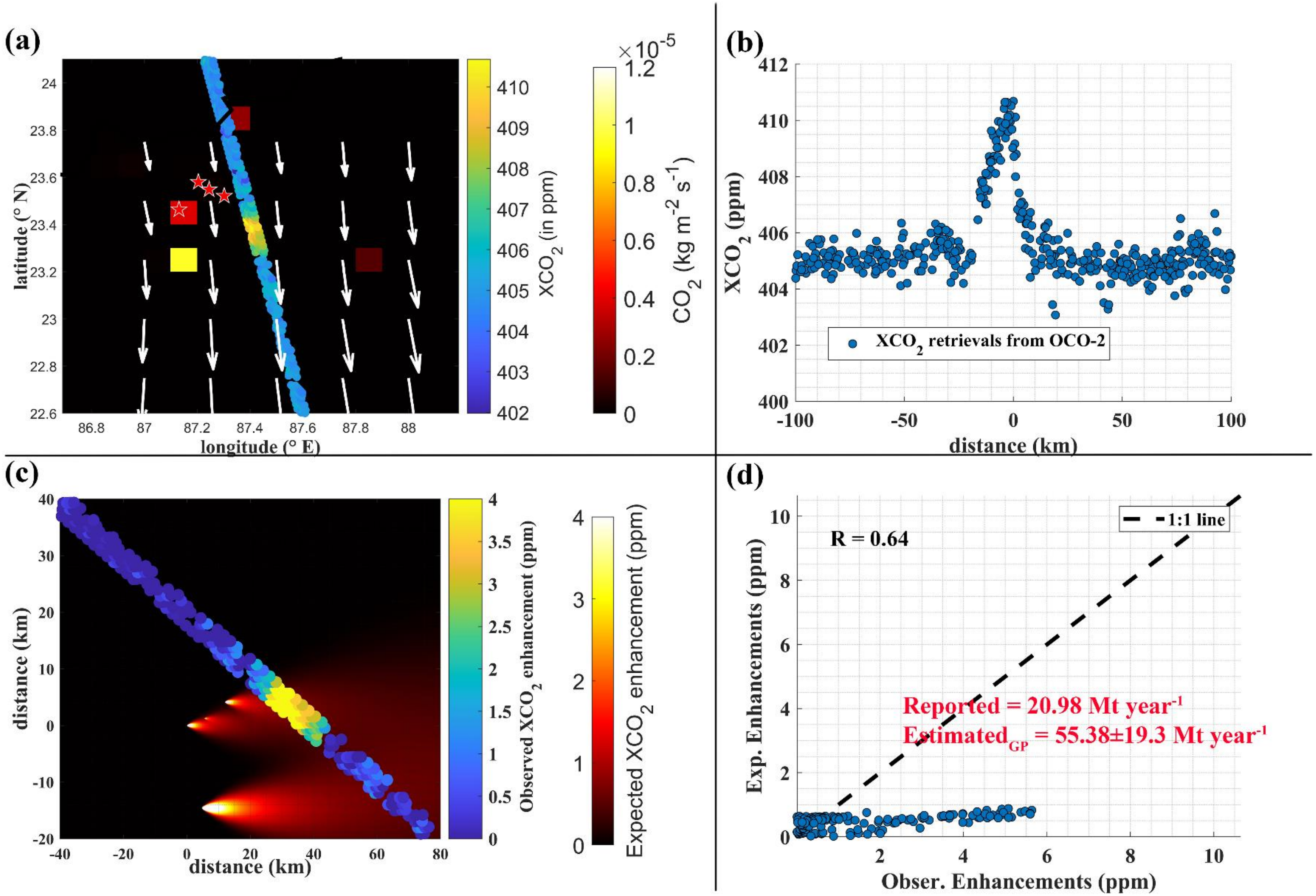

Cluster-9 on "18 January 2017"

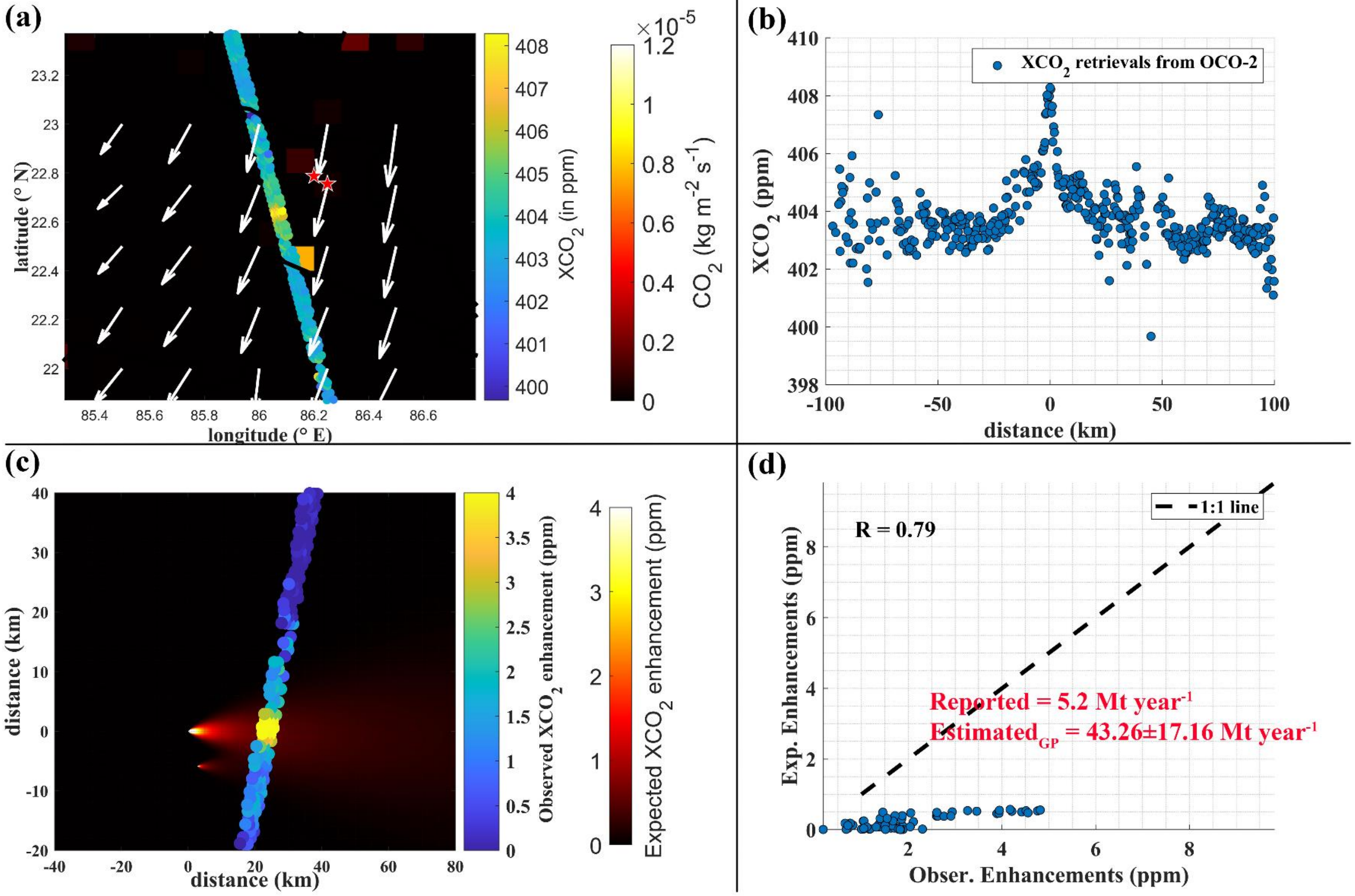

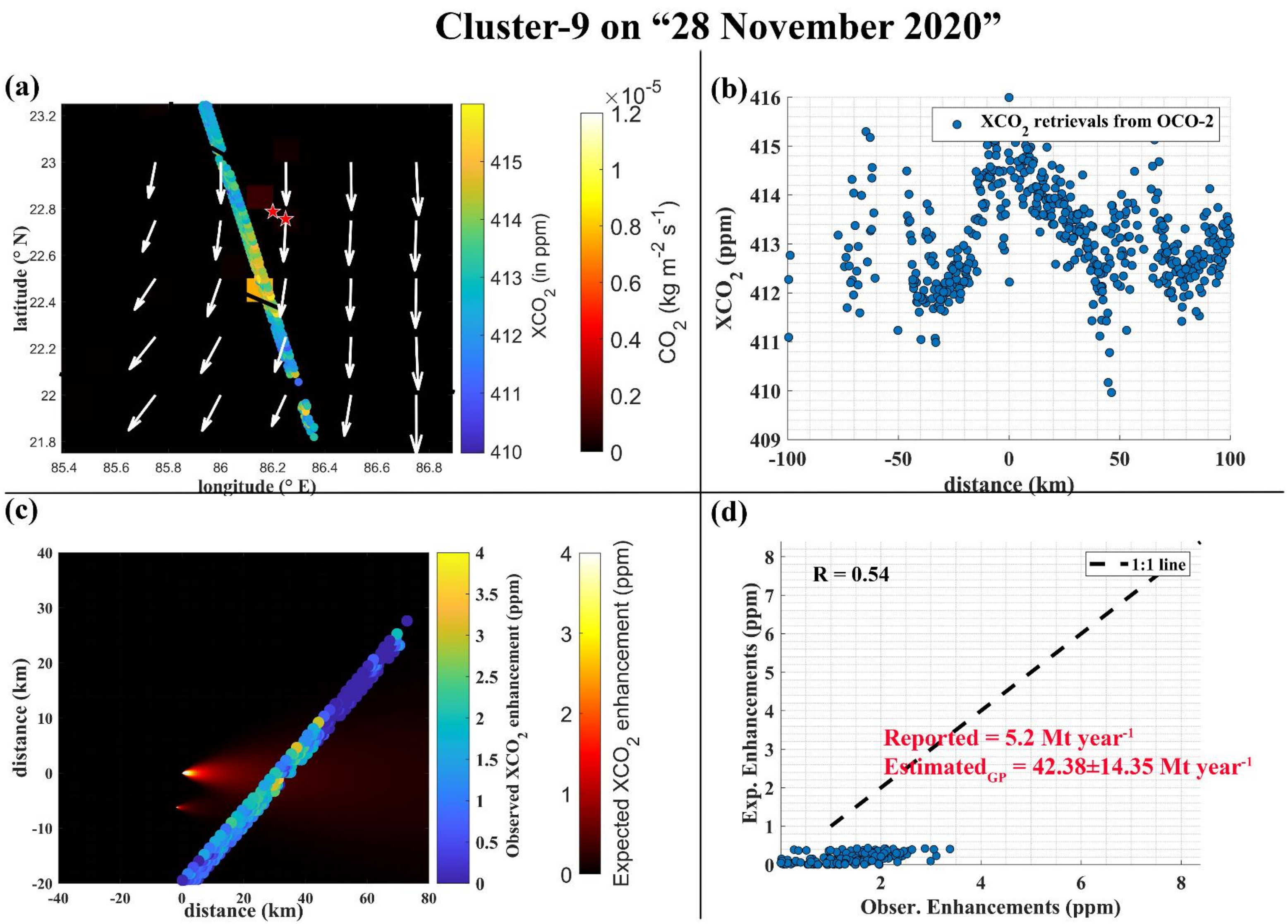
Cluster-9 on "28 November 2020"
(a)
latitude (° N)
longitude (° E)
XCO2 (in ppm)
CO2 (kg m-2 s-1)
×10-5
(b)
XCO2 retrievals from OCO-2
XCO2 (ppm)
distance (km)
(c)
distance (km)
Observed XCO2 enhancement (ppm)
Expected XCO2 enhancement (ppm)
(d)
R = 0.54
1:1 line
Reported = 5.2 Mt year-1
EstimatedGP = 42.38±14.35 Mt year-1
Exp. Enhancements (ppm)
Obser. Enhancements (ppm)

Cluster-9 on "31 October 2022"

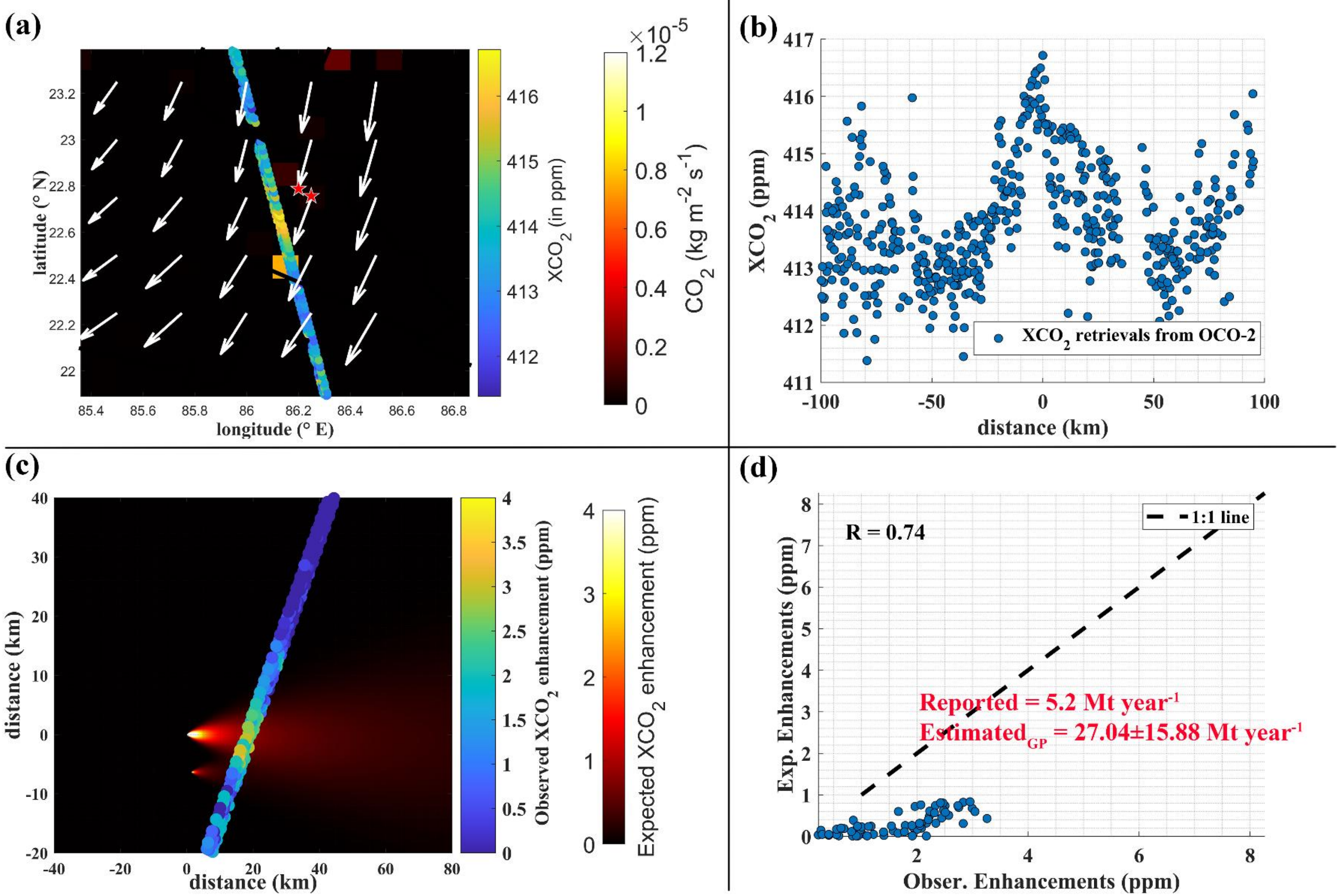

Cluster-9 on "7 January 2018"

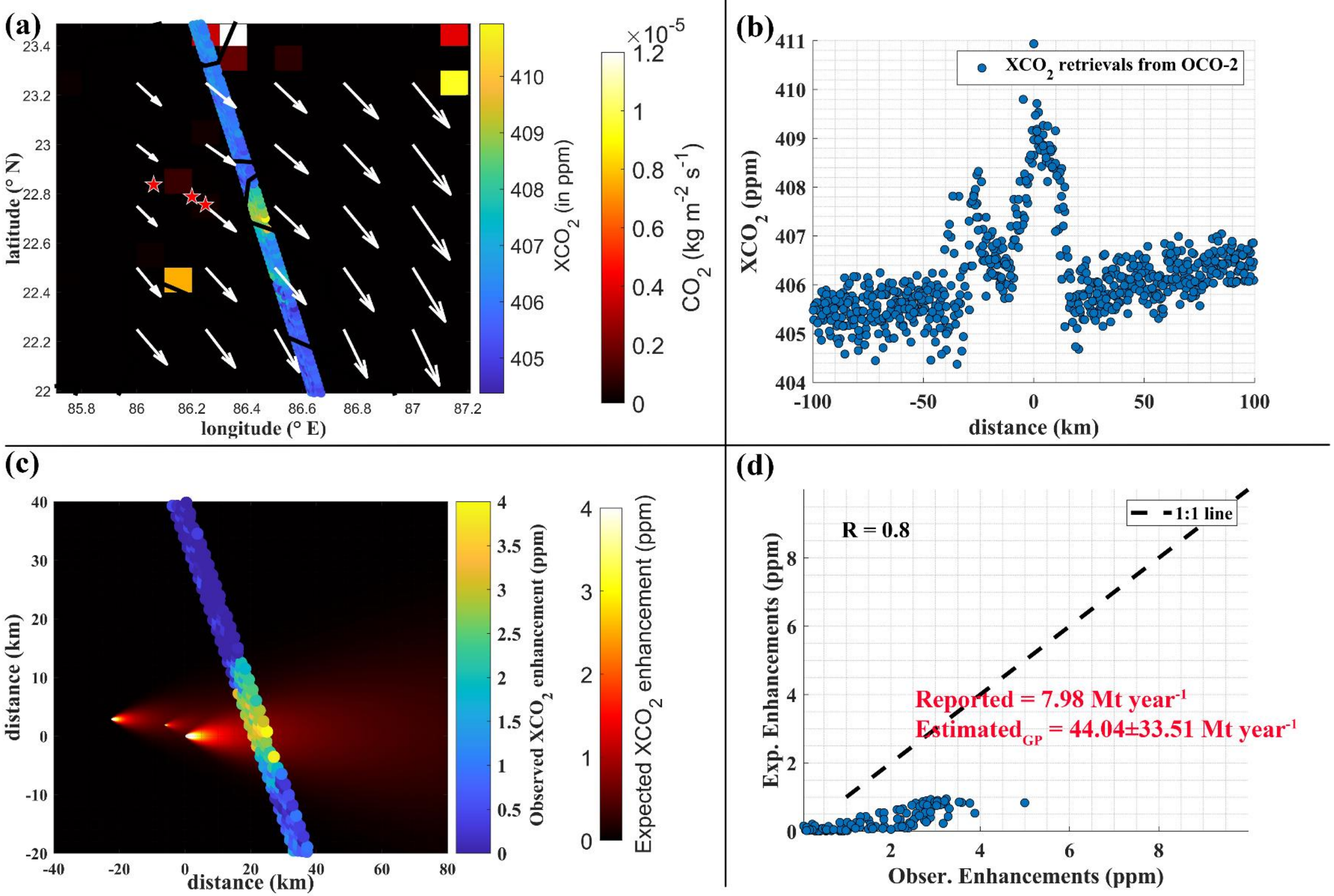

Cluster-10 on "30 December 2014"

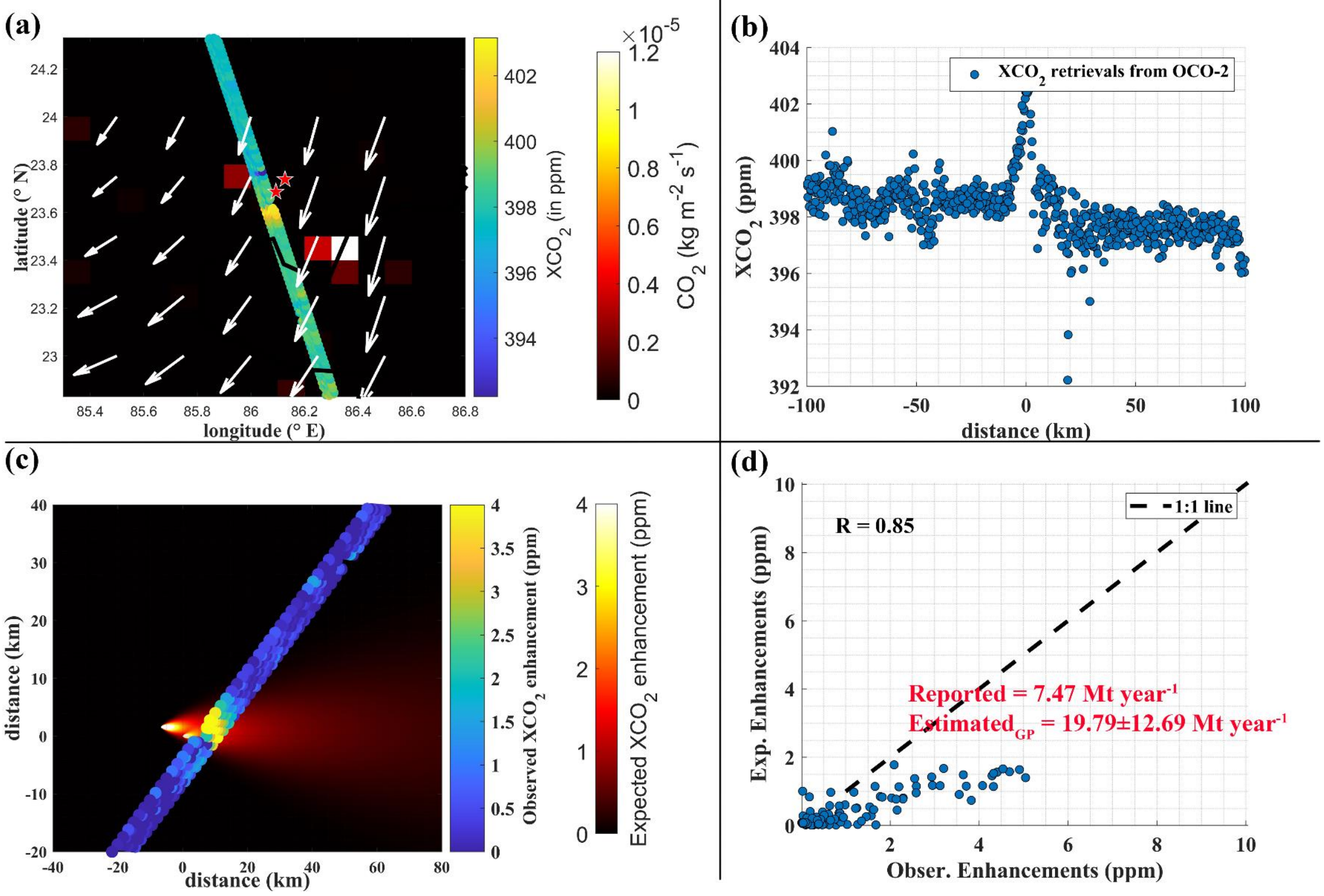

Cluster-10 on "31 January 2015"

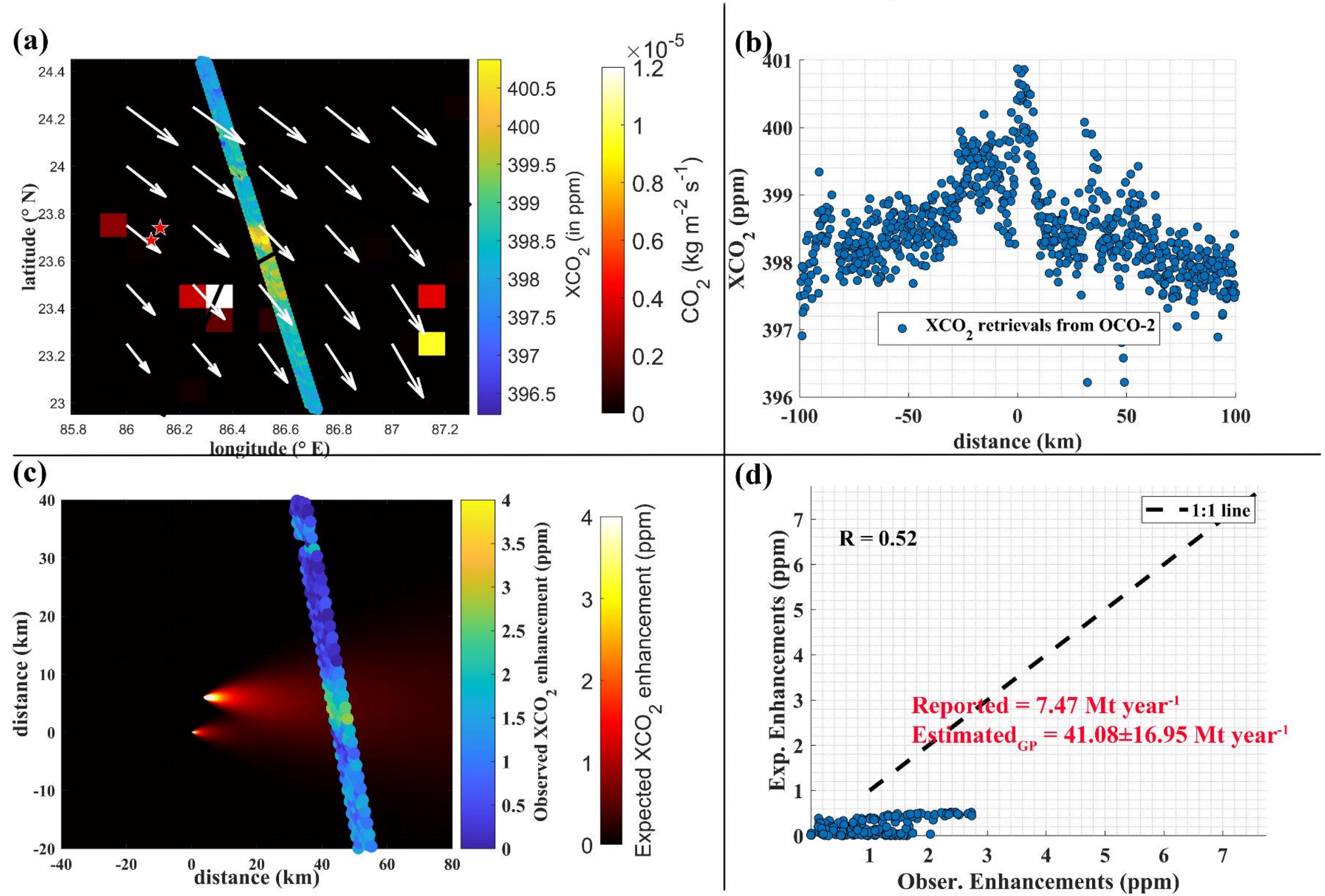

Cluster-10 on “18 January 2020”

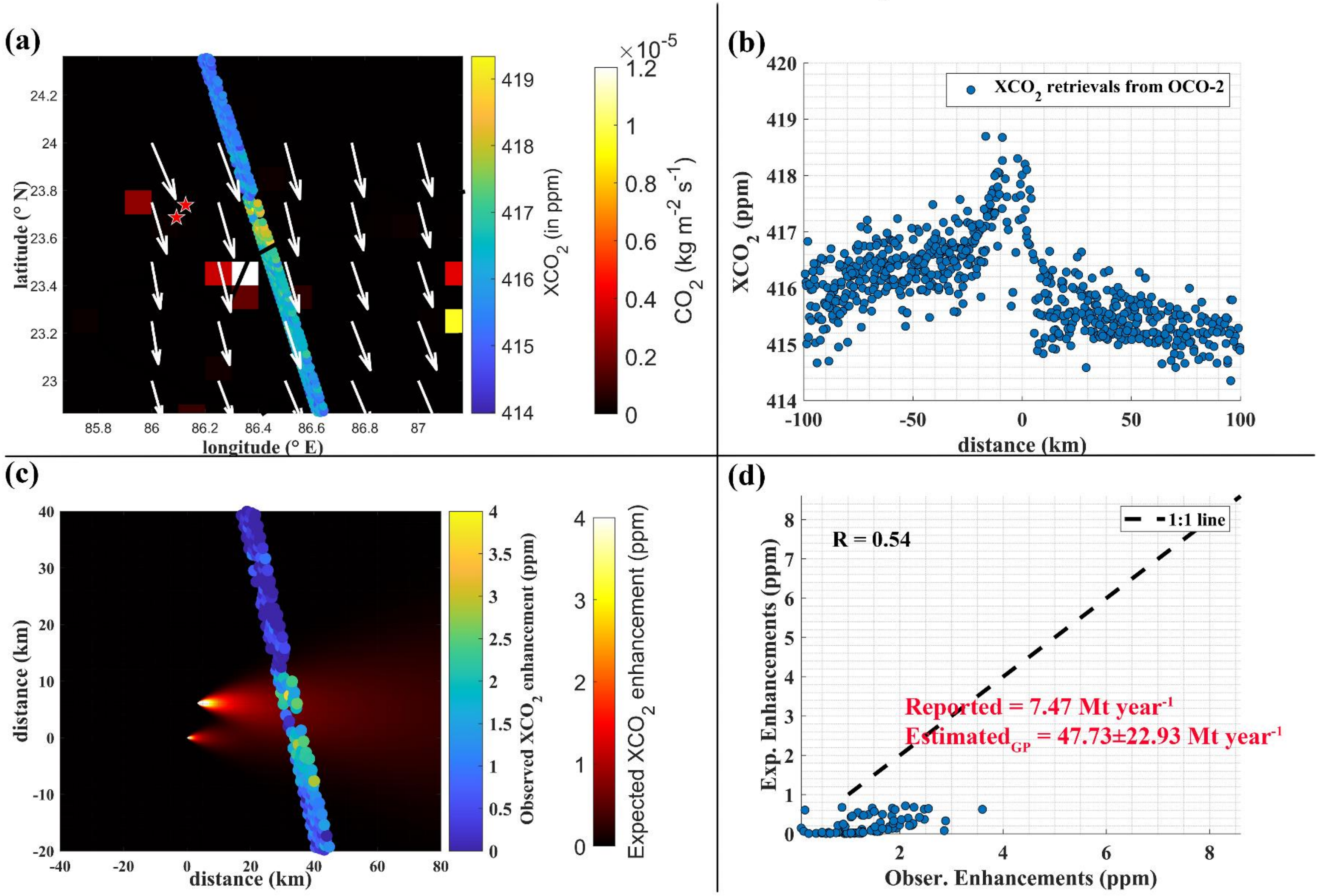

Cluster-10 on “31 January 2021”

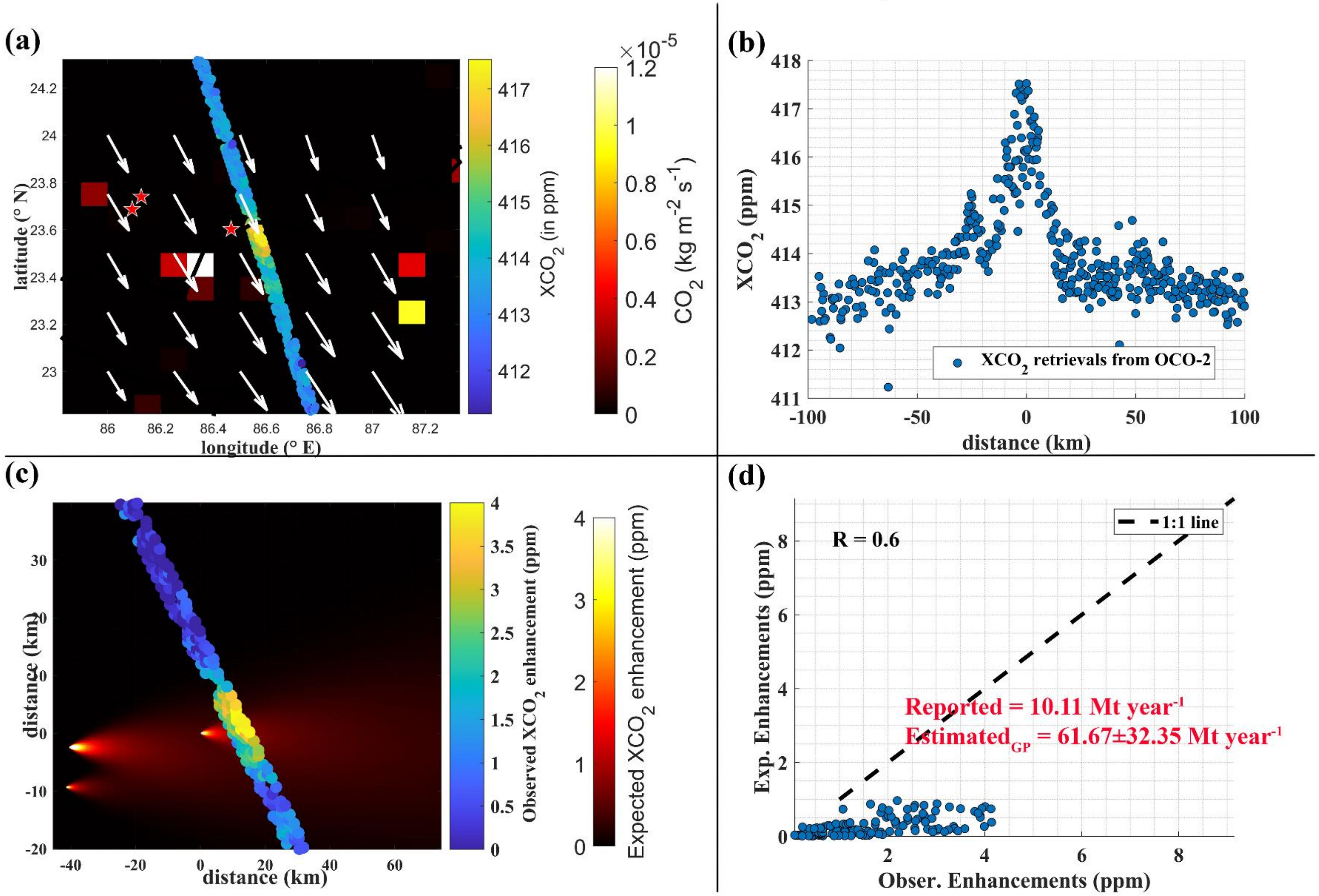

Bhilai Steel power station on "7 January 2017"

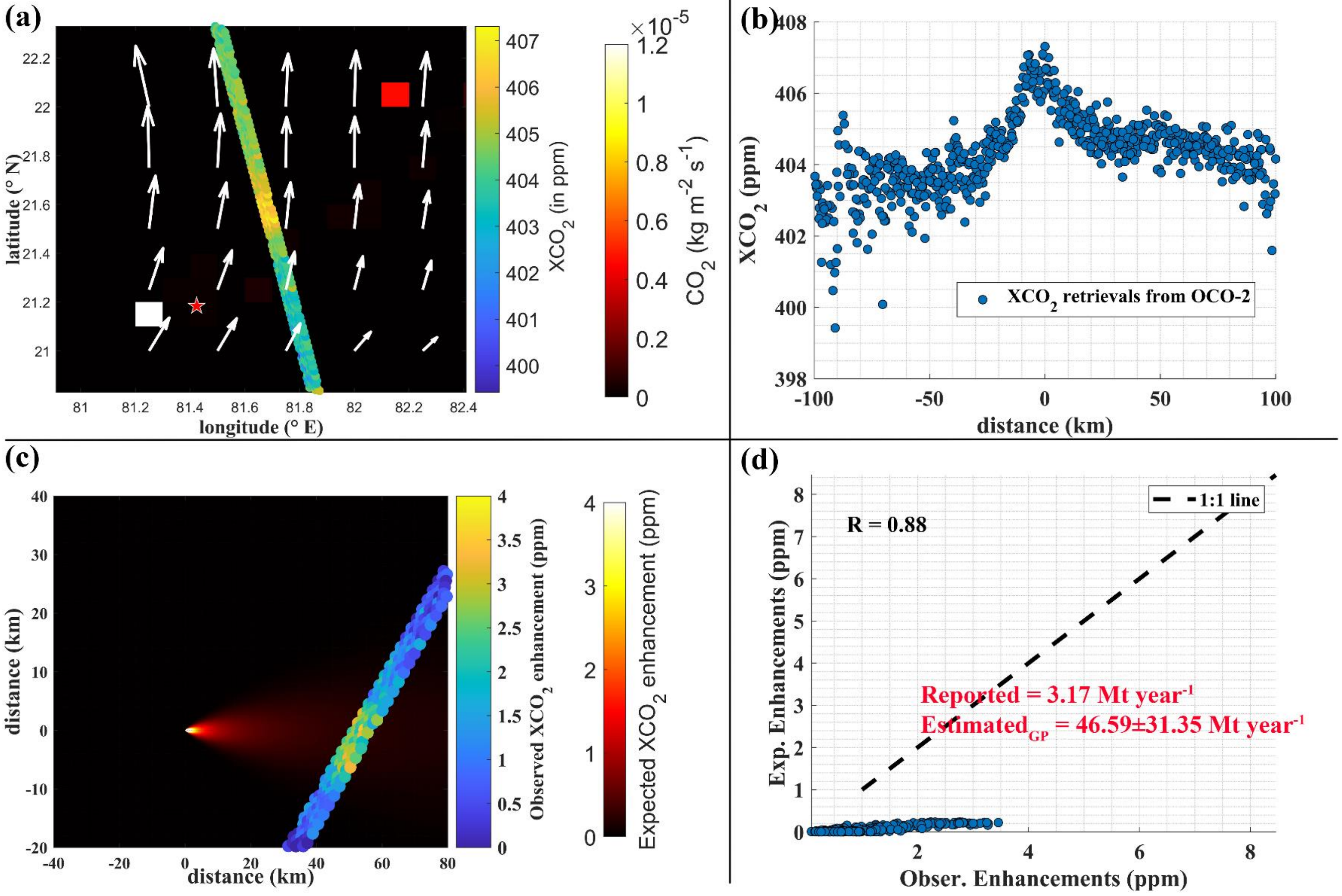

Bhilai steel power station on "8 February 2017"

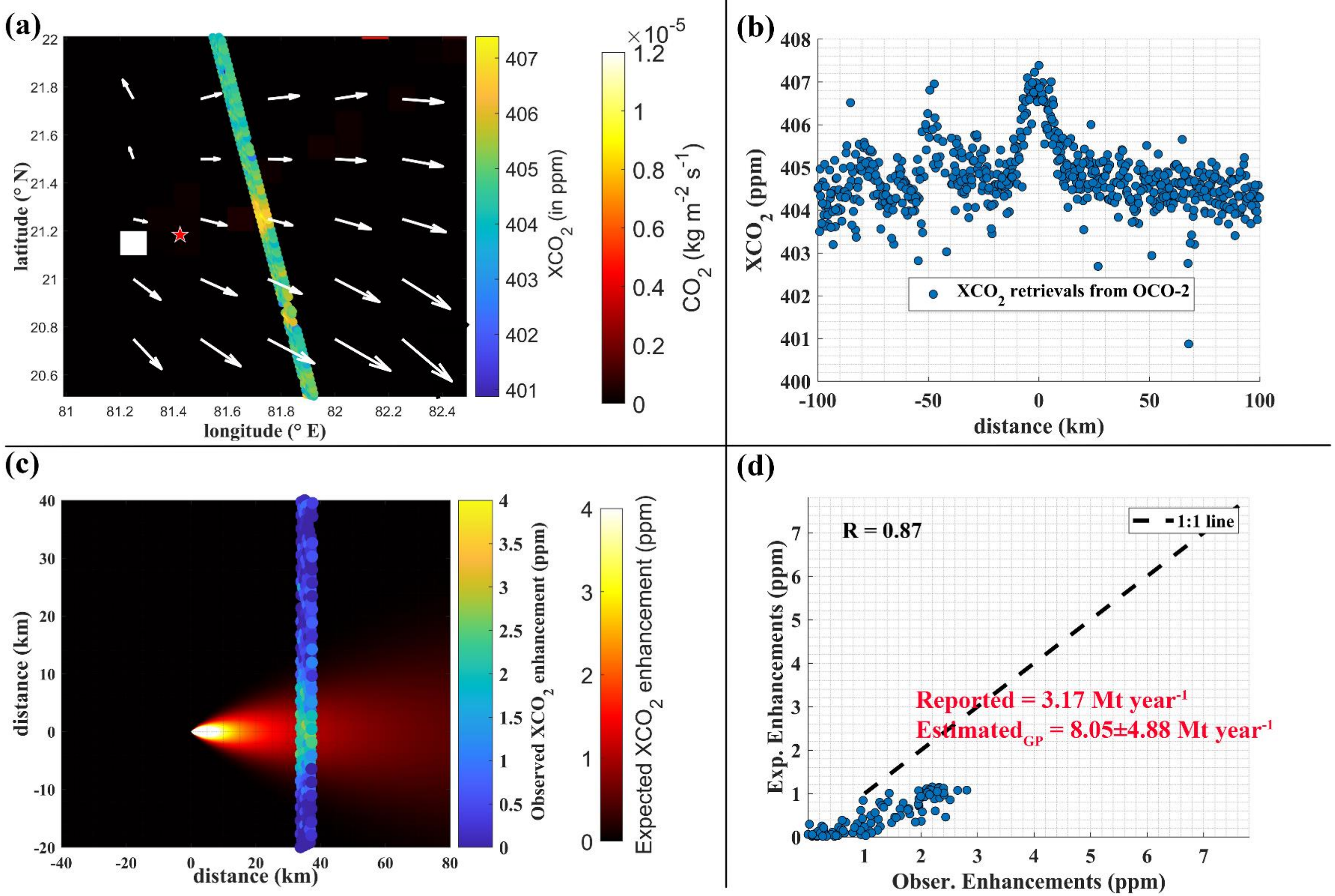

Bhilai steel power station on "14 February 2019"

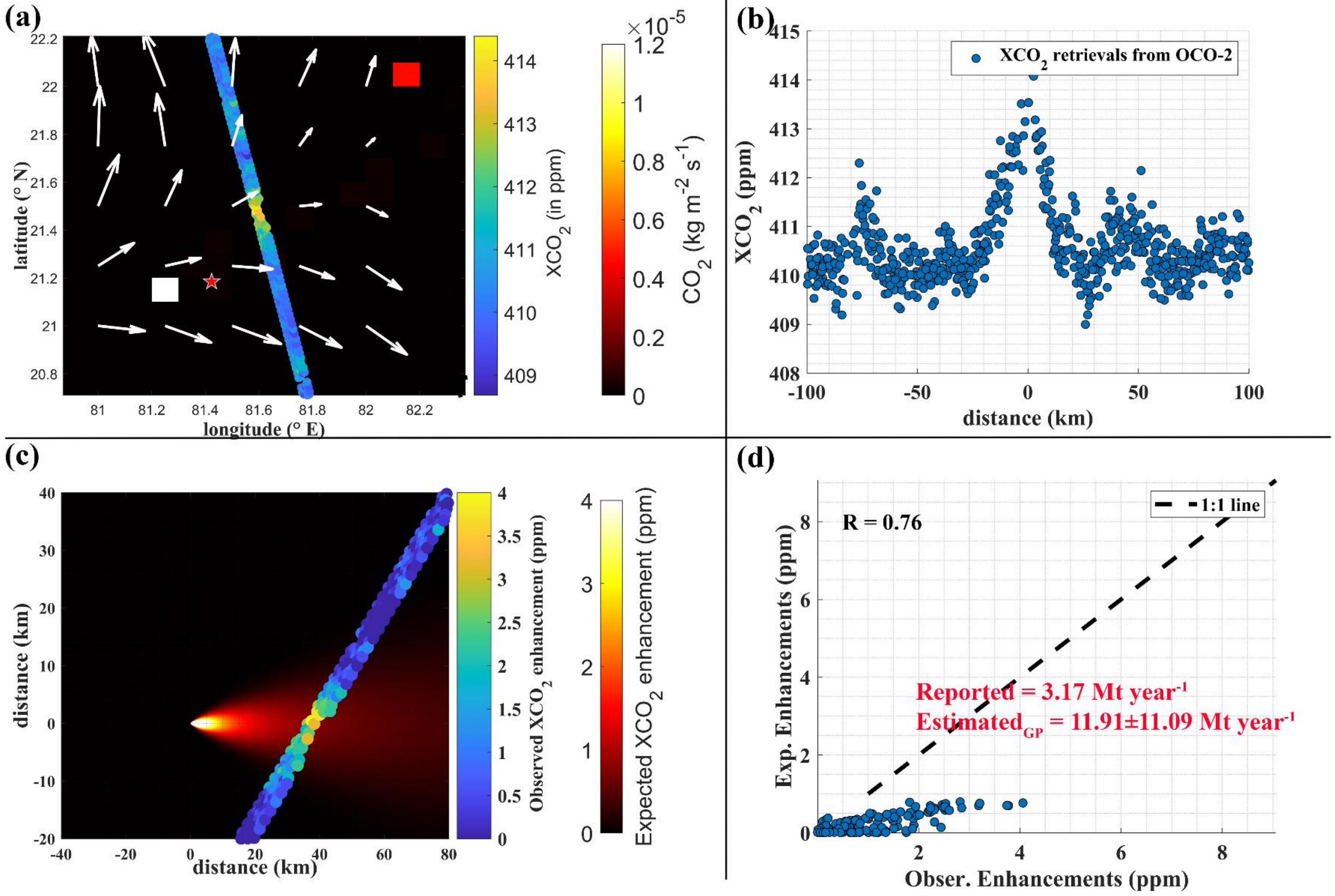

Bhilai steel power station on "10 February 2020"

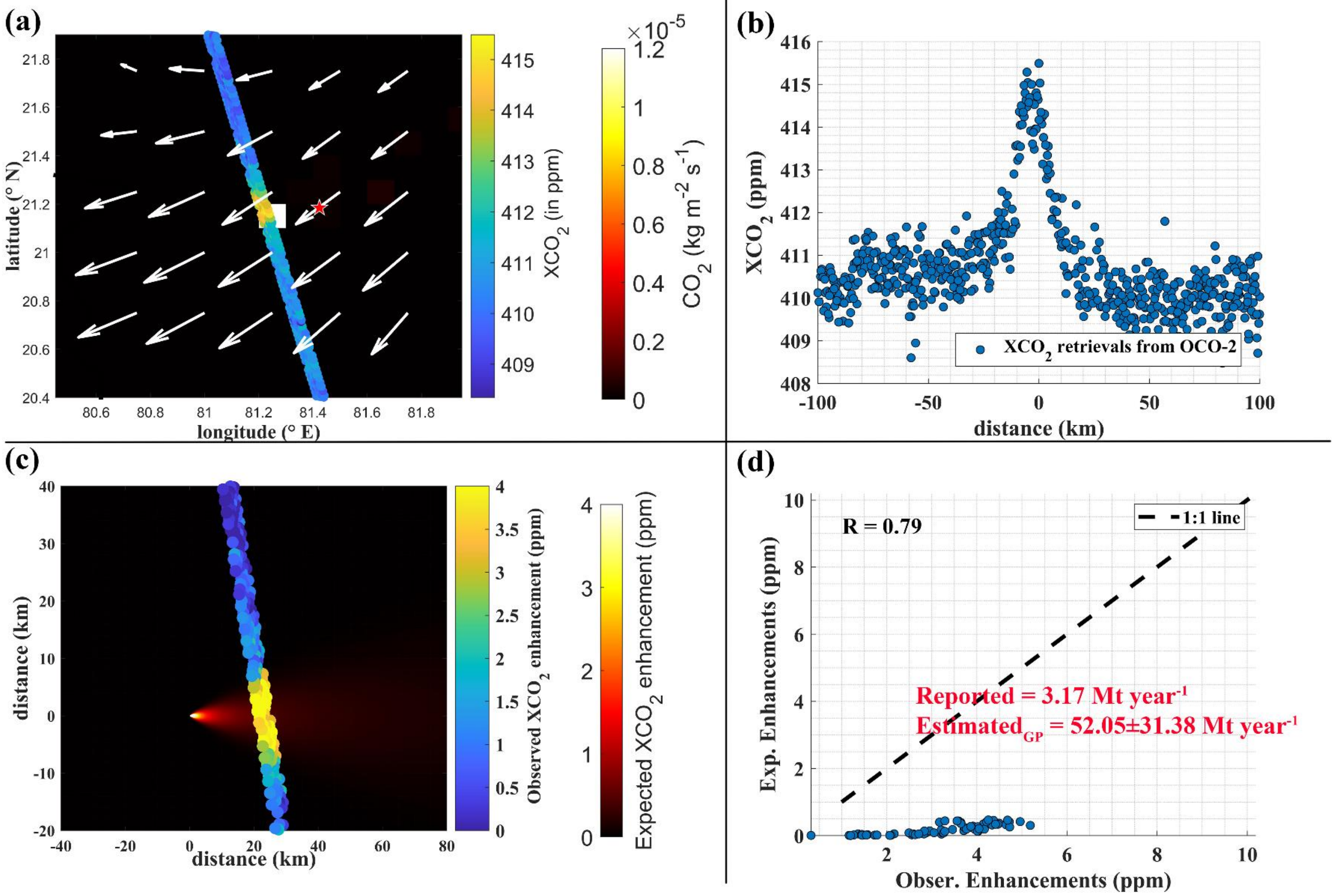

Cluster 11 on "4 March 2018`"

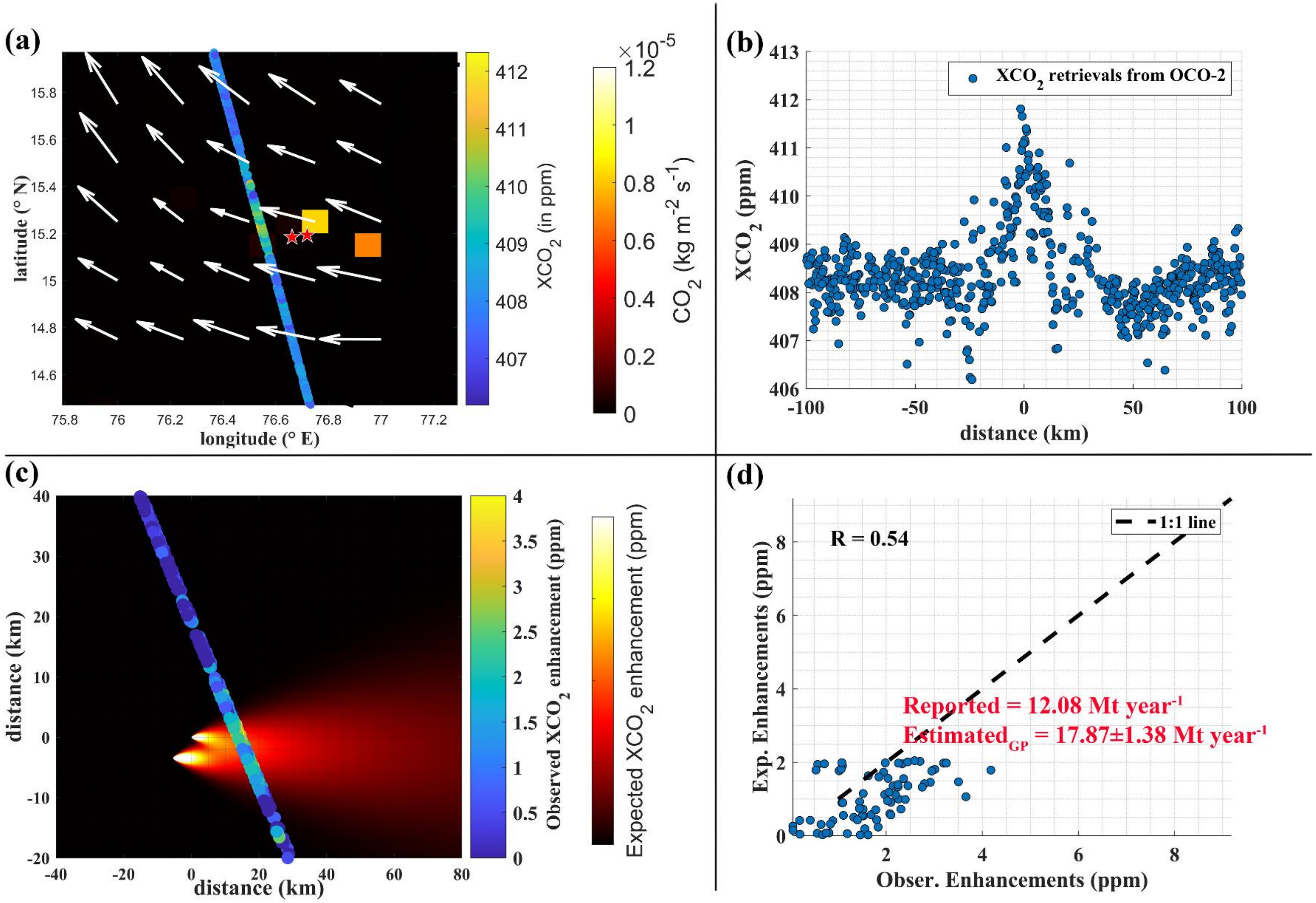

Cluster 11 on "18 January 2019"

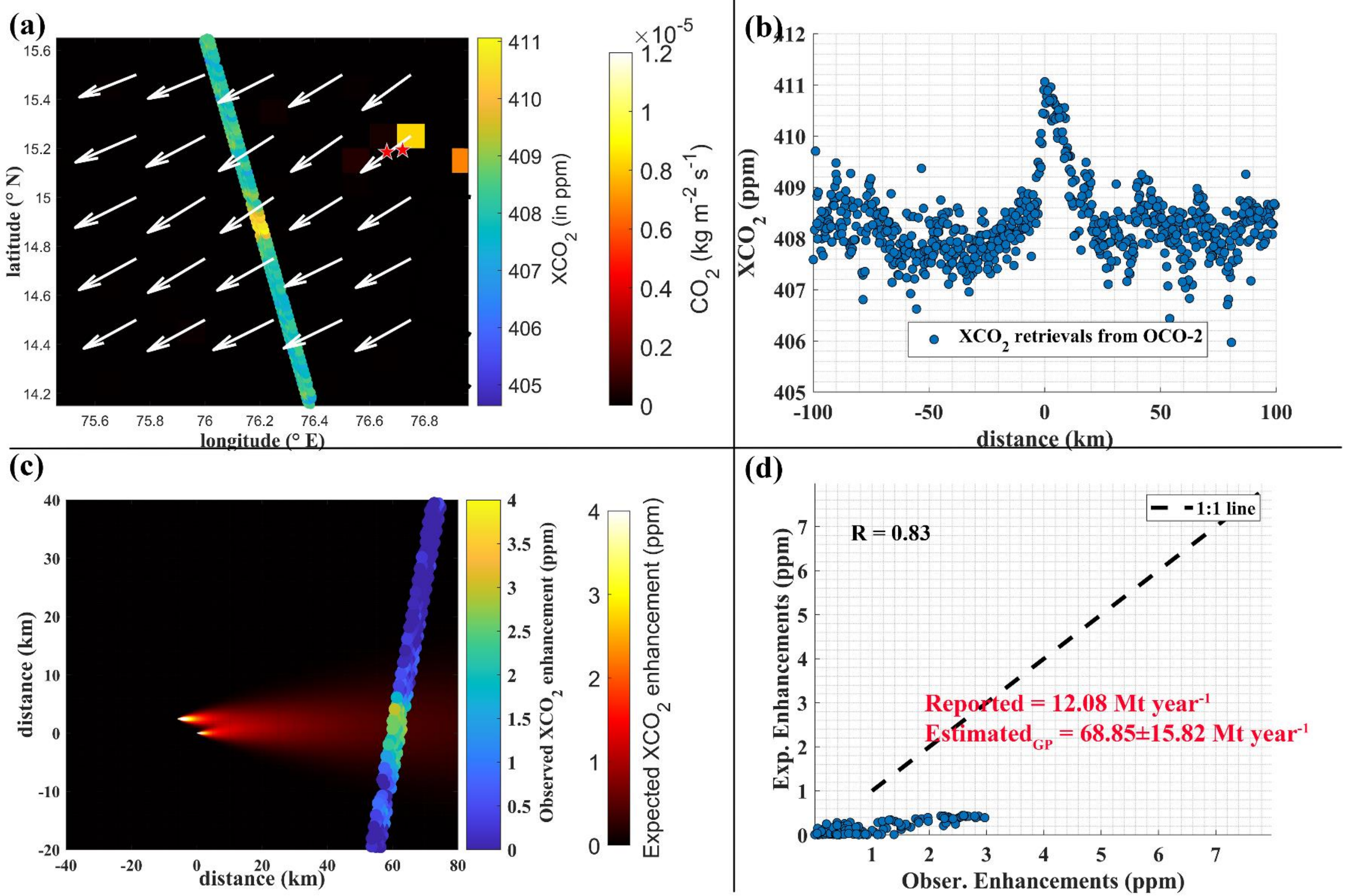

Cluster 11 on "24 February 2021"

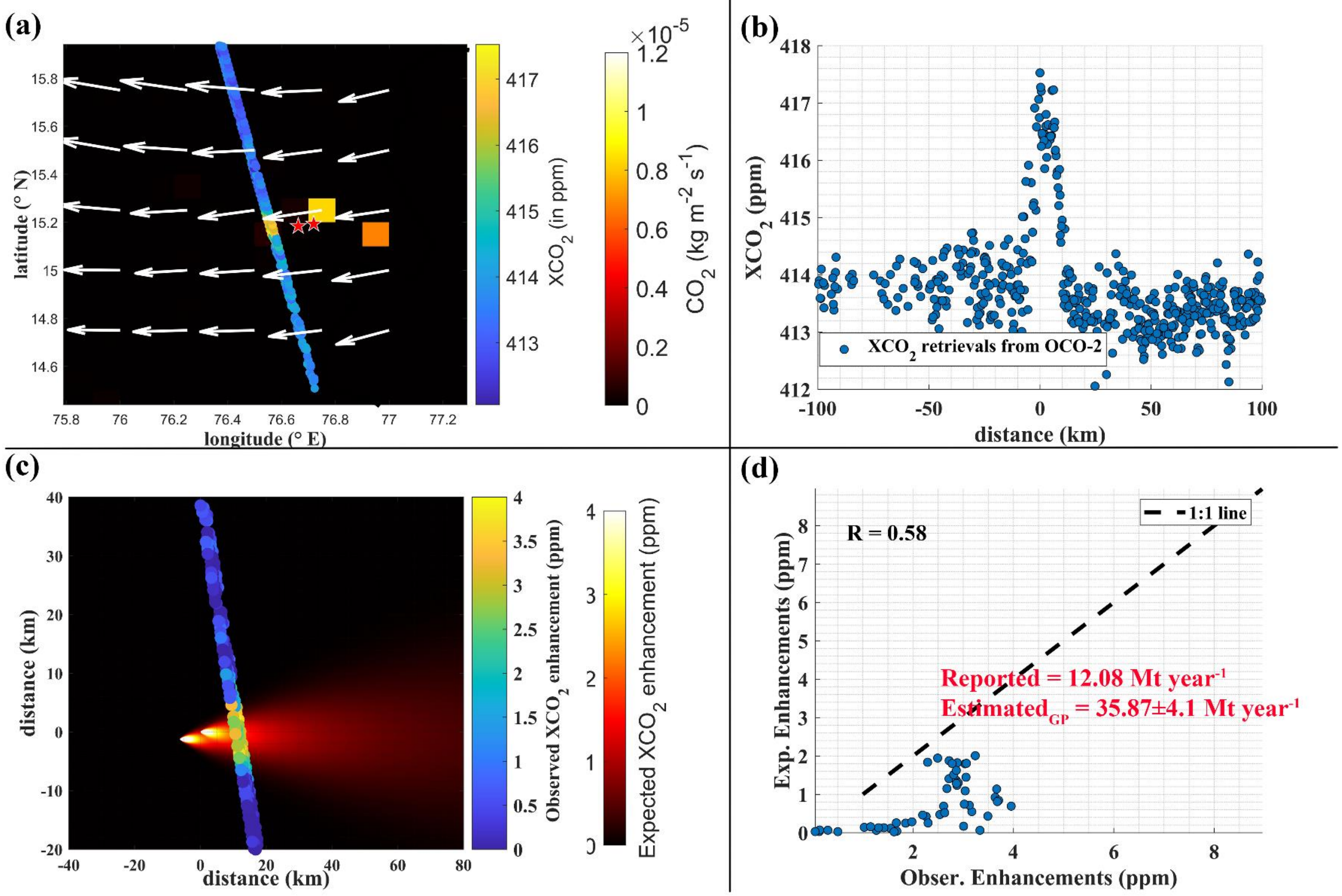

Cluster 12 on "24 February 2015"

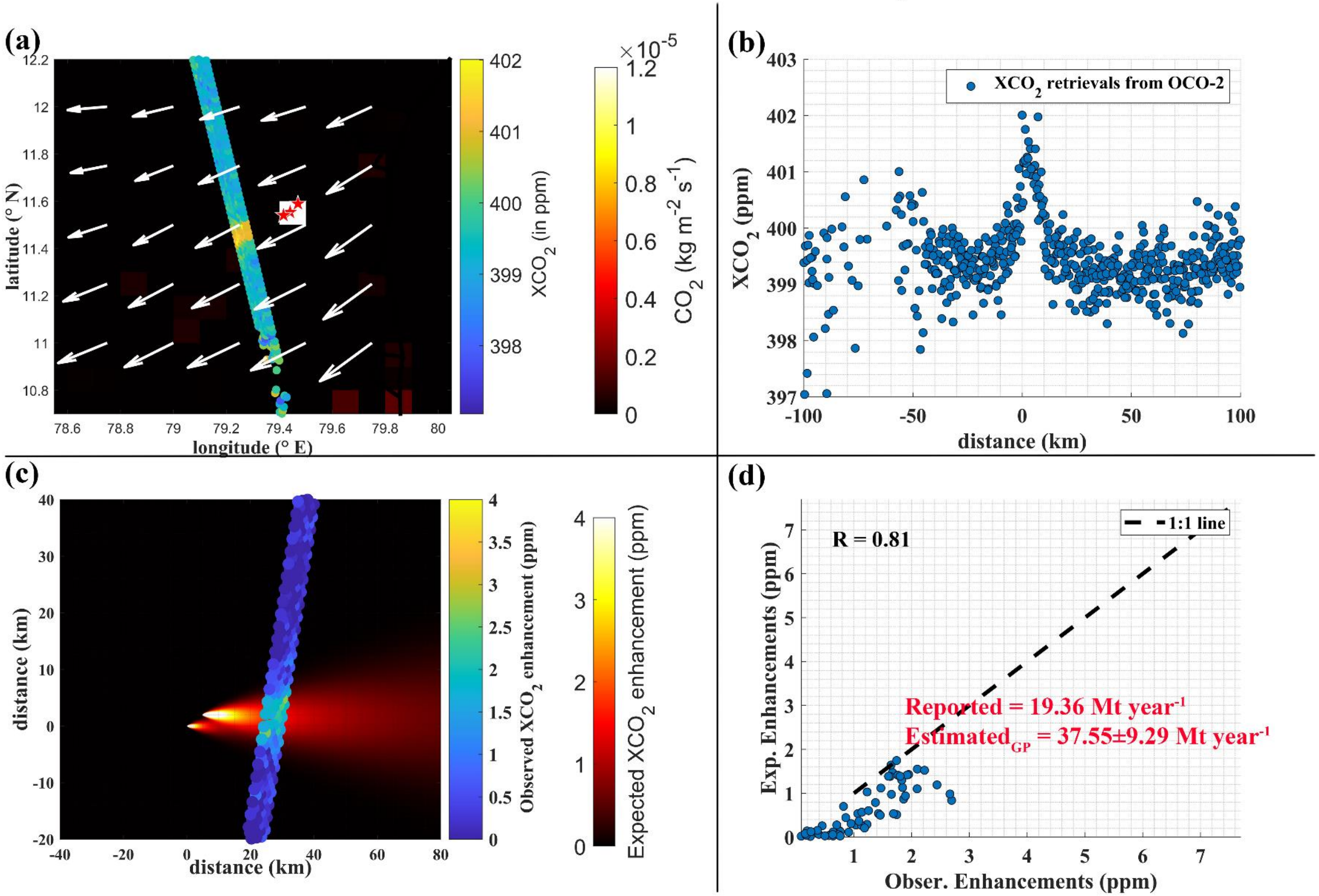

Kawai Thermal Power Project on "30 January 2017"

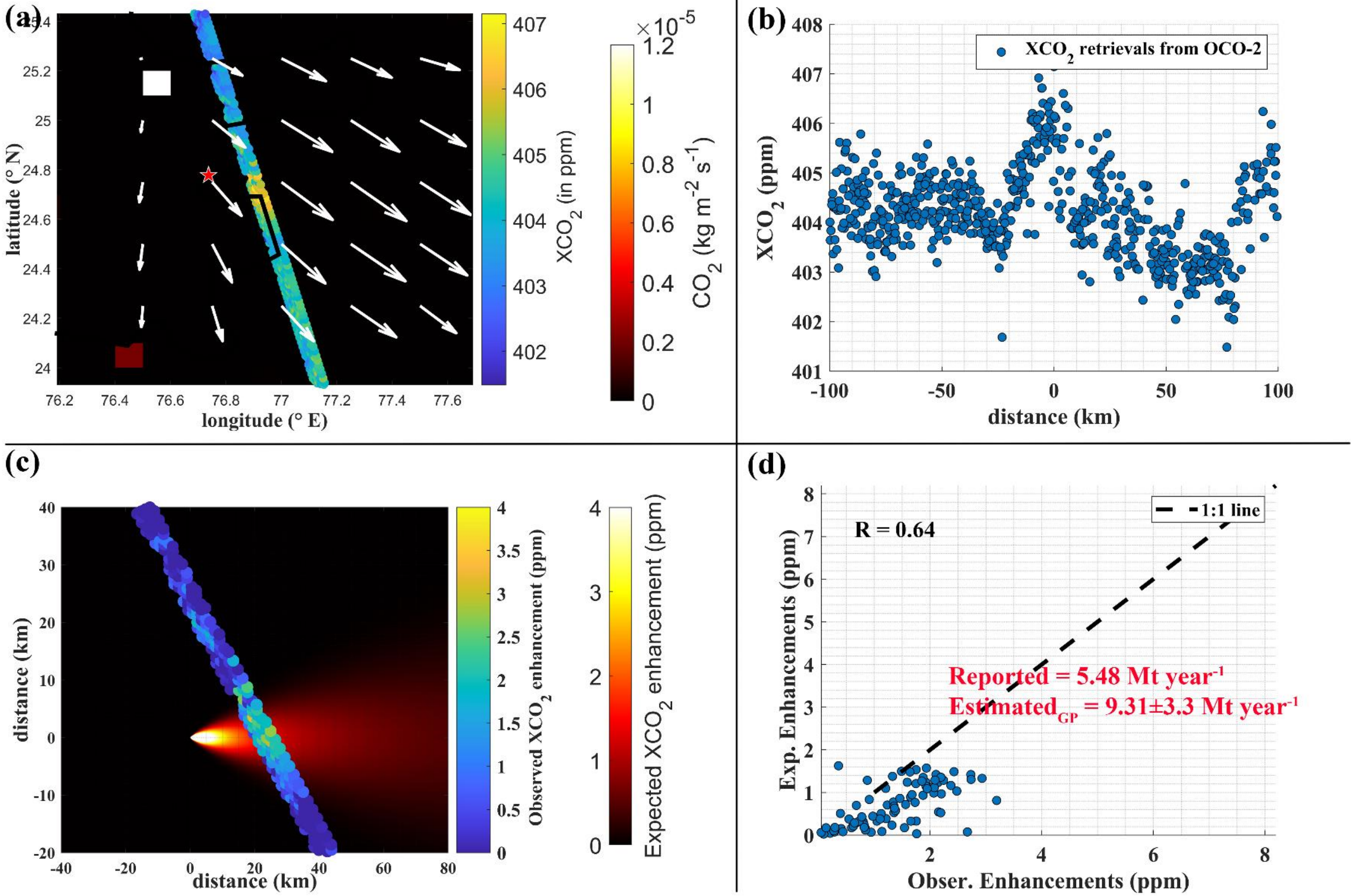

Supplement: Supplementary file 1 — Supporting Information S1 [file EFT2-12-0-s001.pdf]
